# Correlation between Higher Aging Males’ Symptoms Scores and a Higher Risk of Lower Urinary Tract Symptoms

**DOI:** 10.3390/jcm12247528

**Published:** 2023-12-06

**Authors:** Takashi Kawahara, Sahoko Ninomiya, Teppei Takeshima, Tomoki Saito, Hiroki Ito, Mitsuru Komeya, Hisashi Hasumi, Yasushi Yumura, Kazuhide Makiyama, Hiroji Uemura

**Affiliations:** 1 Departments of Urology and Renal Transplantation, Yokohama City University Medical Center, Yokohama 232-0024, Japan; sahoko.ninomiya@gmail.com (S.N.); pug_daikichi@yahoo.co.jp (H.I.); hu0428@yokohama-cu.ac.jp (H.U.); 2 Department of Urology, Yokohama City University Graduate School of Medicine, Yokohama 236-0004, Japan; komeyam@yokohama-cu.ac.jp (M.K.); hasumi@yokohama-cu.ac.jp (H.H.); makiya@yokohama-cu.ac.jp (K.M.); 3 Department of Reproduction Center, Yokohama City University Medical Center, Yokohama 232-0024, Japan; teppei_t@yokohama-cu.ac.jp (T.T.); saito.tom.kf@yokohama-cu.ac.jp (T.S.); yumura@yokohama-cu.ac.jp (Y.Y.)

**Keywords:** lower urinary tract syndrome, LOH, questionnaire

## Abstract

Background: Late-onset hypogonadism (LOH) is a condition caused by the decline of testosterone levels with aging and is associated with various symptoms, including lower urinary tract symptoms (LUTSs). Although some reports have shown that testosterone replacement treatment for LOH improves LUTSs, no large study has revealed a correlation between LUTSs and LOH. This study investigated the correlation between the severity of LOH and LUTSs in Japanese males >40 years of age using a web-based questionnaire with the Aging Males’ Symptoms (AMS) scale. Methods: We asked 2000 Japanese males to answer both the AMS and IPSS/QOL questionnaires using a web-based survey. Among these 2000 individuals, 500 individuals were assigned to each age group. Results: The IPSS total score was positively correlated with the severity of AMS (shown as median [mean ± SD]): no/little group, 2 (3.67 ± 5.36); mild group, 6 (7.98 ± 6.91); moderate group, 11 (12.49 ± 8.63); and severe group, 16 (14.83 ± 9.24) (*p* < 0.0001). Conclusions: Individuals with higher AMS values, representing cases with severe LOH symptoms, had a higher risk of experiencing nocturia and LUTSs than those with lower AMS values.

## 1. Introduction

Late-onset hypogonadism (LOH) is a condition caused by the decline in testosterone levels with aging and is associated with various symptoms, including physical, psychological, and sexual disturbances [1,2]. Unlike menopause, in which all women undergo a nearly complete cessation of gonadal estrogen production, gonadal androgen production in men decreases progressively after the age of 40 years, but androgen levels among individuals remain highly variable. Previous studies have shown that serum testosterone levels decline between 0.4% and 2.6% per year in men after 40 years of age [3,4]. The symptoms of LOH include loss of muscle mass, increased body fat, anemia, osteoporosis, depressed mood, decreased vitality, sweating, hot flashes, loss of libido, and erectile dysfunction (ED). Among these, sexual symptoms, ED, and reduced libido are the most important symptoms related to LOH [5]. Liu et al. reported that the overall prevalence of androgen deficiency was 24.1% using the criterion of a total testosterone (TT) level < 300 ng/dL and 16.6% using the criterion of both TT < 300 ng/dL and free testosterone (FT) < 5 ng/dL. The prevalence of symptomatic androgen deficiency was 12.0%. Older age, obesity, and diabetes mellitus were associated with a significantly higher risk of androgen deficiency and symptomatic androgen deficiency in the Taiwanese population [6].

LUTSs are symptoms associated with LOH. LUTSs can lead to urinary tract infection and upper urinary tract damage and can significantly decrease the quality of life [7]. LUTSs are prevalent in elderly men and women and are characterized by incomplete voiding, hesitancy, diminished stream, and storage indications such as urgency with incontinence, increased frequency, and nocturia. Significant morbidity and a potential increase in the risk of falls are observed [8,9]. Recent studies have shown that testosterone replacement therapy (TRT) can improve male LUTSs [10]. Although LOH worsens male LUTSs, most studies have investigated LUTSs in patients with LOH who present to the hospital [7,11,12]. To date, no large-scale study has investigated the correlation between LOH severity and LUTSs in patients who have not visited a hospital.

The Aging Males’ Symptoms (AMS) scale, which is also validated in Japanese individuals, is widely accepted for the assessment and monitoring of symptoms of LOH; however, patients with an AMS score of 17–26 are not diagnosed with LOH [13]. In 1999, the Aging Males’ Symptoms (AMS) scale was developed as a symptom-profiling approach in Germany [14] In the AMS, the severity of the symptoms of LOH is evaluated using the total score derived from all 17 questions, which assess parameters on a five-point scale [14,15]. The International Prostate Symptom Score (IPSS), which includes seven domains and a QOL score, is widely used to assess the symptoms of male LUTSs [16]. The first version of the IPSS was created in 1992 by the American Urological Association (AUA). The IPSS consists of seven questions related to symptoms experienced in the last month, including a feeling of incomplete bladder emptying, frequency of urination, intermittency of urine stream, urgency of urination, weak stream, straining, and waking at night to urinate [17]. The current study used the Japanese language to validate IPSS and QOL scores to assess storage and voiding symptoms [18]. The questionnaire assessed voiding and storage symptoms.

The present study investigated the correlation between LOH severity and LUTSs in Asian Japanese males aged ≥40 years using a web-based questionnaire.

## 2. Materials and Methods

We asked 2000 Japanese males to answer both the AMS and IPSS/QOL questionnaires using a web-based survey (Freeasy, ibridge Inc., Tokyo, Japan) in August 2021. This survey was approved by the Institutional Review Board of Yokohama City University (Yokohama, Japan) [F220900015]. Of these 2000 individuals, 500 were assigned to each of the following age groups: 40–49, 50–59, 60–69, and ≥70 years. We randomly selected patients from 40,000 individual panels conducted by a web-based company. The AMS and IPSS/QOL questionnaires, which were validated in Japanese, were answered on a website.

LOH symptoms were assessed using the AMS score as no/little (17–26 points), mild (27–36 points), moderate (37–49 points), or severe (≥50 points) [14]. LUTSs were assessed through IPSS, and the severity was categorized as follows: mild (0–7 points), moderate (8–19 points), and severe (20–35 points) [14]. To compare the risk of voiding and storage dysfunction according to the severity of AMS, voiding dysfunction was assessed by the sum of IPSS Q1, Q3, Q5, and Q6, whereas storage dysfunction was assessed by the sum of IPSS Q2, Q4, and Q7 [19]. Nocturia was defined as a score of ≥2 points for IPSS Question 7. We also used the QOL index to assess the quality of daytime life in individuals with LUTSs.

### Statistical Analyses

Participants’ characteristics and scores were analyzed using *t*-tests and chi-square tests. One-factor analysis of variance (ANOVA) was used to evaluate the association between AMS scores and urinary symptoms. Statistical analyses were performed using GraphPad Prism software ver.10 (GraphPad Software, La Jolla, CA, USA). Statistical significance was set at *p* < 0.05.

## 3. Results

**Figure 1:**
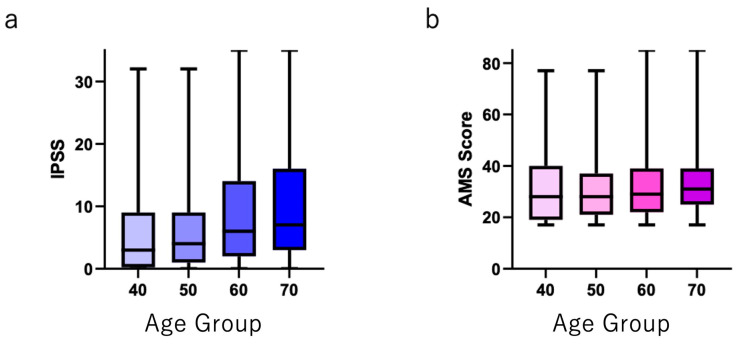
(**a**) Total IPSS and (**b**) total AMS scores in each age group.

Five hundred individuals in each age group (40–49 years, 50–59 years, 60–69 years, and ≥70 years) answered both AMS and IPSS/QOL questionnaires. The distribution of the IPSS total, QOL, and AMS scores is shown in Supplementary Figure S1. Figure 1 shows that the IPSS total score was positively correlated with age. The IPSS total scores in each age group were as follows (shown as mean ± SD): 40–49 years, 5.95 ± 7.25; 50–59 years, 6.09 ± 6.79; 60–69 years, 8.71 ± 8.31; and ≥70 years, 10.14 ± 8.90 (*p* < 0.0001). The AMS scores (shown as the mean ± SD) were almost same in each age group: 40–49 years, 31.29 ± 13.6; 50–59 years, 30.48 ± 11.96; 60–69 years, 31.26 ± 11.55; and ≥70 years, 32.59 ± 11.06 (*p* = 0.0491).

The individual IPSS scores (Q1–Q7), IPSS total score, and QOL index for each AMS score group are shown in Table 1. The IPSS total score was positively correlated with the severity of AMS (shown as median [mean ± SD]): no/little group, 2 (3.67 ± 5.36); mild group, 6 (7.98 ± 6.91); moderate group, 11 (12.49 ± 8.63); and severe group, 16 (14.83 ± 9.24) (*p* < 0.0001). The QOL index was also positively correlated with the severity of AMS (shown as median [mean ± SD]): no/little group, 2 (2.19 ± 1.40); mild group, 3 (3.00 ± 1.38); moderate group, 3.5 (3.57 ± 1.40); and severe group, 4 (3.87 ± 1.50) (*p* < 0.000). Other IPSS scores, including Q7 (nocturia), were also positively correlated with AMS severity (Table 1).

**Figure 2:**
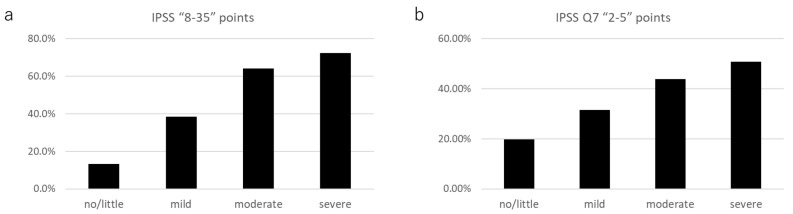
The prevalence of (**a**) IPSS ≥ 8; and (**b**) IPSS Q7 (nocturia) ≥ 2 in each AMS severity group.

The prevalence of an IPSS total score of ≥8 in the AMS severity groups was as follows: no/little group, 13.3% (111 of 834); mild group, 38.3% (225 of 587); moderate group, 64.2% (244 of 380); and severe group, 72.4% (144/199) (Figure 2a). The prevalence of nocturia in the AMS severity groups was as follows: no/little, 19.8% (165 of 834); mild, 31.5% (185 of 587); moderate, 43.9% (167 of 380); and severe, 50.8% (101 of 199) (Figure 2b).

**Figure 3:**
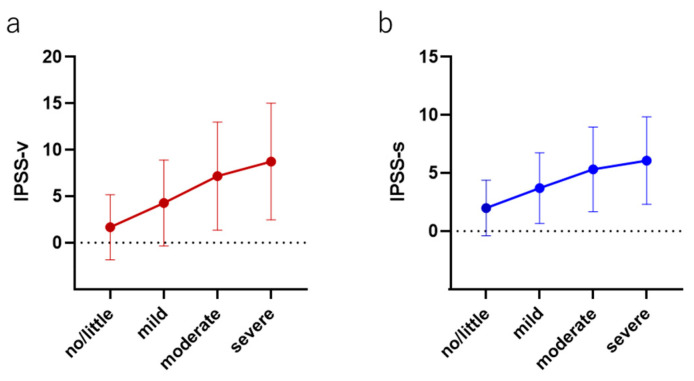
The correlation between both (**a**) voiding and (**b**) storage symptoms in each AMS severity group.

The median (mean ± SD) IPSS-v scores (voiding dysfunction) in each group were as follows: no/little group, 0 (1.67 ± 3.49); mild group, 3 (4.28 ± 4.68); moderate group, 6 (7.18 ± 5.81); and severe group, 9 (8.79 ± 6.29) (*p* > 0.0001). The median (mean ± SD) IPSS-s scores (storage dysfunction) were as follows: no/little group, 1 (2.00 ± 2.40); mild group, 3 (3.70 ± 3.04); moderate group, 5 (5.32 ± 3.64); and severe group, 6 (6.08 ± 3.77) (*p* > 0.0001). The relative scores of the IPSS-v and IPSS-s normalized by the score in the no/little group were positively correlated with the severity of AMS (IPSS-v: mild, 2.56; moderate, 4.29; and severe, 5.23; IPSS-s: mild, 1.85; moderate, 2.66; and severe, 3.04) (Figure 3).

**Figure 4:**
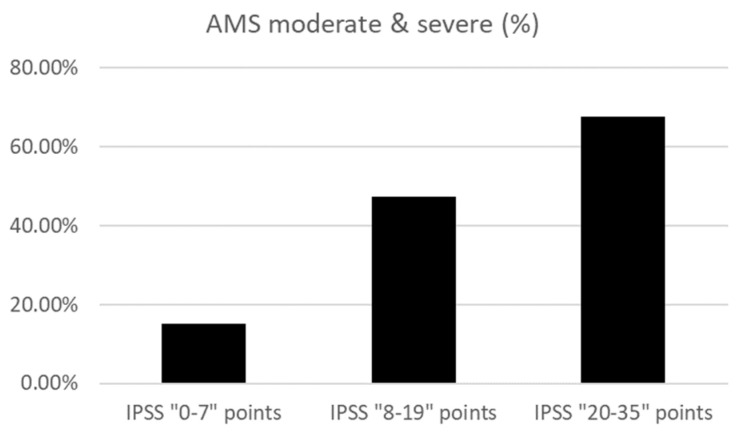
The prevalence of moderate and severe AMS in each IPSS score group.

Figure 4 shows the prevalence of moderate or severe AMS in each IPSS severity group: IPSS total score 0–7 points, 15.0% (191 of 1276); IPSS total score 8–19 points, 47.4% (236 of 499); and IPSS total score 20–35 points, 67.6% (152 of 225). Raw data, including age, having a spouse or not, having children, and household income, are shown in the Supplementary Materials.

## 4. Discussion

This study revealed that patients with higher AMS scores had a higher prevalence of LUTSs and nocturia. These results also support previous studies that have reported that LOH symptoms worsen urinary symptoms [11]. Most previous studies have examined patients with LOH symptoms who visited hospitals. Therefore, studies to date have focused on patients with symptoms or other diseases, and not on those who were asymptomatic or had no occasion to seek medical attention. The present study assessed all individuals, including asymptomatic individuals. To date, this is the largest study to assess the correlation between LUTSs and increased AMS scores. Our findings support the notion that symptoms of LOH influence LUTSs.

The detailed mechanism underlying the association between LOH and LUTSs remains unclear. However, one possibility is that testosterone regulates nitric oxide (NO) production and that decreased testosterone levels result in lower NO production. Decreasing NO levels increase smooth muscle and pelvic muscle tone, which worsens LUTSs [12,20]. Amano et al. reported that patients with LOH had LUTSs, particularly voiding symptoms [1,21]. The present study also supported the previous study in that a stronger correlation was observed between the severity of AMS and the worsening of voiding symptoms in comparison to the severity of AMS and the worsening of storage symptoms.

This study also supported previous studies, as LUTSs worsened with an increase in the severity of LOH symptoms. In daily clinical practice, urologists sometimes encounter cases of male patients with LUTSs whose symptoms are not improved by treatment, including alpha-1 blockers, 5-alpha reductase, and endourological surgery. Based on evidence to support the efficacy of testosterone replacement treatment for the improvement of LUTSs in patients with LOH symptoms, ruling out LOH symptoms using the AMS score may be useful for determining appropriate treatment strategies for male patients with LUTSs.

Another potential mechanism is that low testosterone concentration influences an elevated blood glucose level, a worsening lipid profile, increased body weight, and increased blood pressure, causing problems such as atherosclerosis [22,23,24]. Based on these mechanisms, LUTSs may be induced by LOH syndrome, and testosterone replacement treatment may restore these phenomena [22]. On the other hand, LUTSs sometimes improve after androgen deprivation therapy with 5-alpha reductase treatment due to a reduction in the prostate volume. We speculate that LUTSs worsen for a variety of reasons. LOH may be one of the factors that worsens LUTSs.

The present study was associated with some limitations. First, we evaluated LOH symptoms based on the AMS questionnaire and did not examine serum testosterone levels. Therefore, the clinical diagnosis was not determined. The AMS is widely used to assess the severity of LOH in daily clinical practice. Despite this limitation, a large number of individuals were evaluated in this short-term study, including men with no symptoms related to LOH. Second, this study did not assess other factors that can affect LUTSs, including smoking, diabetes mellitus, and hypertension [25,26]. Further studies are needed that include the evaluation of detailed information on the patients’ current state, history, and other factors.

## 5. Conclusions

Individuals with higher AMS scores, which reflect severe LOH symptoms, have a higher risk of nocturia and LUTSs.

## Figures and Tables

**Table 1:** IPSS/QOL score. The individual IPSS scores in each AMS severity group.

Median (Mean ± SD)						
	All	No/Little	Mild	Moderate	Severe	
Number (%)	2000	834 (41.7%)	587 (29.4%)	300 (15.0%)	199 (10.0%)	*p* Value
IPSS						
Q1	0 (1.09 ± 1.55)	0 (0.43 ± 1.02)	1 (1.15 ± 1.45)	1 (1.81 ± 1.72)	2 (2.33 ± 1.86)	<0.0001
Q2	1 (1.36 ± 1.55)	0 (0.69 ± 1.15)	1 (1.46 ± 1.45)	2 (2.09 ± 1.66)	2 (2.44 ± 1.72)	<0.0001
Q3	0 (0.94 ± 1.54)	0 (0.35 ± 1.02)	0 (0.91 ± 1.42)	1 (1.64 ± 1.80)	2 (2.11 ± 1.91)	<0.0001
Q4	0 (0.91 ± 1.45)	0 (0.22 ± 0.90)	0 (0.95 ± 1.41)	1 (1.58 ± 1.68)	1 (1.93 ± 1.84)	<0.0001
Q5	1 (1.40 ± 1.79)	0 (0.62 ± 1.28)	1 (1.50 ± 1.73)	2 (2.33 ± 1.93)	2 (2.62 ± 1.96)	<0.0001
Q6	0 (0.76 ± 1.43)	0 (0.27 ± 0.90)	0 (0.72 ± 1.34)	1 (1.40 ± 1.75)	1 (1.69 ± 1.83)	<0.0001
Q7	1 (1.27 ± 1.28)	1 (0.97 ± 1.24)	1 (1.29 ± 1.21)	1 (1.64 ± 1.31)	2 (1.70 ± 1.26)	<0.0001
total	5 (7.72 ± 8.05)	2 (3.67 ± 5.36)	6 (7.98 ± 6.91)	11 (12.49 ± 8.63)	16 (14.83 ± 9.24)	<0.0001
QOL index	3 (2.85 ± 1.54)	2 (2.19 ± 1.40)	3 (3.00 ± 1.38)	3.5 (3.57 ± 1.40)	4 (3.87 ± 1.50)	<0.0001

## Data Availability

We uploaded raw data in supplementary file.

## Supplementary Materials

The following supporting information can be downloaded at: https://www.mdpi.com/article/10.3390/jcm12247528/s1, Figure S1: Distribution of (a) total IPSS score, (b) QOL score, and (c) AMS score. Supplementally File S1: Raw data from web questionnaire.
