# Supplementary material for: Correlation between Higher Aging Males’ Symptoms Scores and a Higher Risk of Lower Urinary Tract Symptoms
Source: J Clin Med. 2023 Dec 6;12(24):7528. doi: 10.3390/jcm12247528 (PMC10744136; doi:10.3390/jcm12247528)
Supplement: Supplementary file 1 [file jcm-12-07528-s001.zip › jcm-2706289-Supplementally File S1.pdf]

No	Age	married/unmarried	annual household income (JPY)	child	IPSS Q1	IPSS Q2	IPSS Q3	IPSS Q4	IPSS Q5	IPSS Q6	IPSS Q7	QOL	AMS Q1	AMS Q2	AMS Q3	AMS Q4	AMS Q5	AMS Q6	AMS Q7	AMS Q8	AMS Q9	AMS Q10	AMS Q11	AMS Q12	AMS Q13	AMS Q14	AMS Q15	AMS Q16	AMS Q17
1	59	unmarried	less than 1,000,000 JPY	no	0	0	0	0	0	0	0	3	1	1	1	1	1	1	1	1	1	1	1	1	1	1	1	1	1
2	44	unmarried	3,000,000-4,000,000 JPY	no	0	0	0	0	0	0	0	0	1	1	1	1	1	1	1	1	1	1	1	1	1	1	1	1	1
3	50	unmarried	3,000,000-4,000,000 JPY	no	0	0	0	0	0	0	1	1	1	2	3	5	5	3	3	1	5	2	5	5	5	1	1	1	1
4	74	married	2,000,000-3,000,000 JPY	yes	4	4	4	4	5	4	3	6	3	4	2	4	4	2	2	2	1	4	1	1	1	1	5	5	5
5	44	married	7,000,000-8,000,000 JPY	yes	4	3	3	2	4	4	4	2	4	5	4	5	5	4	3	4	5	5	5	4	4	3	4	5	4
6	50	married	3,000,000-4,000,000 JPY	no	5	5	5	5	1	1	2	5	4	4	4	4	4	4	4	4	4	4	4	4	4	4	4	4	4
7	64	married	6,000,000-7,000,000 JPY	yes	0	4	3	0	0	0	1	3	3	1	1	1	2	1	1	1	1	1	1	1	1	1	1	1	2
8	43	married	4,000,000-5,000,000 JPY	no	1	1	1	2	3	1	1	2	2	3	2	2	3	2	2	3	3	2	2	3	2	2	3	2	2
9	59	married	8,000,000-9,000,000 JPY	yes	0	1	1	1	1	0	2	4	2	1	1	2	2	3	3	2	2	2	2	3	1	2	2	1	2
10	56	unmarried	5,000,000-6,000,000 JPY	no	0	2	0	1	1	0	1	5	2	2	2	2	1	1	1	1	1	2	1	1	1	1	2	2	2
11	46	married	2,000,000-3,000,000 JPY	no	0	0	0	0	0	0	0	0	1	1	1	1	1	2	3	3	3	3	4	4	4	3	3	2	3
12	48	married	6,000,000-7,000,000 JPY	yes	5	5	5	5	5	5	1	3	2	2	2	2	2	2	2	2	2	2	2	2	2	2	2	2	2
13	50	unmarried	3,000,000-4,000,000 JPY	no	0	0	0	0	0	0	1	1	3	2	3	5	5	4	4	1	5	2	5	5	5	1	1	1	1
14	67	married	1,000,000-2,000,000 JPY	yes	0	0	0	0	0	0	0	1	1	1	1	1	1	1	1	1	1	1	1	1	1	1	1	1	1
15	49	unmarried	3,000,000-4,000,000 JPY	no	3	3	0	2	1	0	2	2	2	2	1	4	3	3	5	5	2	2	5	4	5	1	1	1	1
16	45	married	3,000,000-4,000,000 JPY	yes	0	0	0	0	0	0	1	5	1	1	1	2	2	1	1	2	1	3	1	3	3	1	1	3	1
17	58	unmarried	less than 1,000,000 JPY	no	0	0	0	0	0	0	1	3	1	1	1	1	1	1	1	1	1	1	1	1	1	1	1	1	1
18	76	married	7,000,000-8,000,000 JPY	yes	5	5	5	5	5	0	2	6	2	3	2	3	3	2	2	2	3	3	2	4	2	2	3	3	3
19	55	married	5,000,000-6,000,000 JPY	yes	0	0	0	0	0	0	0	3	1	1	1	1	2	2	1	1	1	2	1	2	1	1	2	2	2
20	46	married	7,000,000-8,000,000 JPY	yes	1	2	2	0	0	0	3	4	2	3	4	3	3	3	3	3	3	3	3	3	3	3	3	3	3
21	70	married	8,000,000-9,000,000 JPY	yes	5	4	5	3	5	5	1	6	2	2	1	1	2	1	1	1	1	2	1	1	1	1	1	1	1
22	55	married	5,000,000-6,000,000 JPY	yes	1	1	1	1	1	0	1	3	1	3	1	1	2	1	1	1	2	3	1	3	2	2	4	4	1
23	48	unmarried	6,000,000-7,000,000 JPY	no	0	1	0	0	0	0	0	1	1	1	1	1	1	1	1	1	1	1	1	1	1	1	1	1	1
24	42	married	3,000,000-4,000,000 JPY	yes	0	0	0	0	0	0	2	3	1	1	1	2	1	2	2	1	1	1	1	1	1	1	1	1	1
25	56	unmarried	5,000,000-6,000,000 JPY	no	0	3	0	1	0	0	1	5	2	2	2	2	1	1	1	1	1	2	1	1	1	1	2	2	2
26	46	unmarried	4,000,000-5,000,000 JPY	no	1	1	1	1	1	1	1	3	1	2	1	2	2	1	2	1	2	1	2	2	1	1	2	2	2
27	45	unmarried	5,000,000-6,000,000 JPY	no	0	0	0	0	0	0	0	0	1	1	1	1	2	1	1	1	1	1	1	1	1	1	1	1	1
28	65	married	5,000,000-6,000,000 JPY	yes	0	4	5	0	3	3	3	5	2	3	1	4	3	3	2	1	1	1	1	1	1	1	3	4	1
29	50	unmarried	4,000,000-5,000,000 JPY	no	0	1	0	0	0	0	0	3	3	3	3	3	3	3	3	3	3	3	3	3	3	3	3	3	3
30	52	married	8,000,000-9,000,000 JPY	yes	4	0	4	0	4	0	0	5	1	2	1	1	1	1	1	1	1	1	1	3	1	1	2	3	1
31	60	unmarried	less than 1,000,000 JPY	no	0	1	0	0	1	0	4	3	1	1	1	1	2	1	1	1	1	2	1	2	1	1	2	2	1
32	86	unmarried	6,000,000-7,000,000 JPY	no	0	0	0	0	0	0	0	3	1	1	1	1	1	1	1	1	1	1	1	1	1	1	1	1	1
33	56	married	5,000,000-6,000,000 JPY	yes	0	1	1	0	1	0	1	3	1	1	1	1	1	1	1	1	1	2	1	2	1	1	1	1	1
34	60	unmarried	3,000,000-4,000,000 JPY	yes	0	2	0	0	0	0	1	3	1	2	1	1	1	1	1	1	1	1	1	2	1	1	1	3	3
35	61	married	4,000,000-5,000,000 JPY	yes	1	1	0	0	0	0	1	3	1	2	1	1	2	1	1	1	1	1	1	3	1	2	2	4	4
36	64	unmarried	3,000,000-4,000,000 JPY	no	0	1	1	1	5	5	3	3	2	4	1	4	3	3	3	1	2	2	1	3	1	1	3	3	3
37	68	married	2,000,000-3,000,000 JPY	yes	1	1	1	0	1	1	0	2	2	2	1	3	3	1	2	2	2	2	1	3	2	3	2	3	3
38	56	married	7,000,000-8,000,000 JPY	yes	0	1	0	0	0	0	0	1	1	1	2	1	2	1	1	1	1	1	1	2	1	1	2	2	1
39	74	married	8,000,000-9,000,000 JPY	no	2	5	0	0	5	2	2	3	3	3	1	3	4	2	2	1	1	4	1	5	1	3	5	5	5
40	67	married	12,000,000-15,000,000 JPY	yes	0	1	0	0	0	0	3	5	1	1	1	1	1	1	1	1	1	3	1	3	1	1	4	4	3
41	50	married	12,000,000-15,000,000 JPY	no	0	0	0	0	0	0	1	2	1	1	1	1	1	1	1	1	1	1	1	1	1	1	1	1	1
42	44	married	7,000,000-8,000,000 JPY	yes	0	0	0	0	0	0	1	1	1	1	1	1	1	1	1	1	1	1	1	1	1	1	1	1	1
43	57	married	5,000,000-6,000,000 JPY	yes	2	4	0	1	2	0	2	4	3	2	2	3	2	3	3	1	3	2	3	4	3	1	3	4	3
44	41	married	4,000,000-5,000,000 JPY	yes	5	1	0	1	2	2	0	3	1	2	1	1	1	1	1	1	2	2	1	4	2	1	3	3	3
45	56	married	5,000,000-6,000,000 JPY	yes	0	0	0	0	0	0	1	3	1	1	1	1	1	1	1	1	1	1	1	1	1	1	1	1	1
46	52	married	10,000,000-12,000,000 JPY	no	0	0	0	0	0	0	0	3	1	1	1	1	1	1	1	1	1	1	1	1	1	1	1	1	1
47	46	unmarried	6,000,000-7,000,000 JPY	yes	5	3	5	0	0	0	0	4	2	3	1	5	5	2	1	1	2	2	1	4	1	1	3	4	3
48	67	married	4,000,000-5,000,000 JPY	yes	1	2	2	0	1	0	2	5	2	2	1	3	2	1	1	1	1	2	1	2	1	1	2	3	3
49	55	married	7,000,000-8,000,000 JPY	yes	0	0	0	0	0	0	0	0	1	1	1	1	1	1	1	1	1	1	1	1	1	1	1	1	1
50	58	unmarried	5,000,000-6,000,000 JPY	no	2	4	5	4	4	4	1	5	2	2	2	3	3	4	4	3	3	3	4	4	3	2	3	2	2
51	71	married	5,000,000-6,000,000 JPY	yes	0	1	1	1	1	0	5	4	3	1	1	3	1	1	1	1	1	3	2	3	3	1	5	5	5
52	70	married	3,000,000-4,000,000 JPY	yes	0	0	0	0	0	0	1	1	1	1	1	2	1	2	1	1	1	2	1	3	1	1	3	1	2
53	71	married	4,000,000-5,000,000 JPY	yes	0	1	0	1	0	0	2	3	1	1	1	2	1	1	1	1	1	3	1	2	1	1	2	2	1
54	79	married	5,000,000-6,000,000 JPY	yes	1	2	5	5	5	3	3	5	2	2	1	4	2	1	4	1	3	4	4	1	1	3	5	5	5
55	72	married	1,000,000-2,000,000 JPY	yes	0	1	0	0	1	0	0	3	2	1	2	2	2	2	1	1	2	4	2	2	2	2	2	2	2
56	72	married	2,000,000-3,000,000 JPY	yes	0	0	0	0	1	0	0	1	1	2	1	1	1	2	2	1	1	2	2	2	2	2	3	4	4
57	69	married	1,000,000-2,000,000 JPY	yes	1	1	0	1	1	0	1	4	3	1	1	3	3	1	1	1	1	1	1	2	1	1	1	1	2

58	72	married	2,000,000-3,000,000 JPY	yes	3	3	0	3	3	3	2	3	2	1	1	1	1	1	1	1	1	2	1	2	1	1	3	3	3
59	75	married	3,000,000-4,000,000 JPY	yes	5	2	5	5	5	5	3	4	2	2	2	2	2	2	2	2	3	3	3	4	4	2	4	4	4
60	74	married	3,000,000-4,000,000 JPY	yes	0	0	0	0	0	0	1	3	1	1	1	1	1	1	1	1	1	1	1	1	1	1	1	1	1
61	43	married	5,000,000-6,000,000 JPY	yes	1	3	1	1	0	1	5	3	3	3	1	1	2	2	2	1	2	3	2	2	1	1	2	3	3
62	53	unmarried	1,000,000-2,000,000 JPY	no	1	1	1	0	1	0	1	4	1	3	1	1	1	1	1	1	2	1	1	2	1	1	2	2	2
63	51	married	10,000,000-12,000,000 JPY	yes	1	5	0	1	0	0	0	3	1	1	1	2	2	4	1	1	1	1	2	2	2	1	1	3	1
64	106	unmarried	2,000,000-3,000,000 JPY	no	0	0	0	0	0	1	1	2	1	1	1	1	1	1	1	3	4	4	5	5	4	4	4	4	1
65	54	married	4,000,000-5,000,000 JPY	yes	0	0	0	0	0	0	1	2	1	1	1	1	3	2	1	1	1	1	1	3	1	2	3	3	1
66	67	married	6,000,000-7,000,000 JPY	yes	0	0	1	0	2	0	2	3	2	2	1	2	2	1	2	1	1	2	2	2	1	1	1	1	1
67	81	married	4,000,000-5,000,000 JPY	yes	5	5	5	4	3	2	3	6	3	2	2	2	2	2	3	3	2	2	2	3	2	2	4	5	5
68	45	married	8,000,000-9,000,000 JPY	yes	0	0	0	0	0	0	0	0	1	1	1	1	1	1	1	1	1	1	1	1	1	1	1	1	1
69	58	married	1,000,000-2,000,000 JPY	no	0	0	0	0	0	0	4	0	1	1	1	1	1	1	1	1	1	1	1	1	1	1	1	1	1
70	60	unmarried	1,000,000-2,000,000 JPY	no	3	5	5	3	5	2	3	5	3	3	2	3	3	3	3	2	2	4	3	3	3	1	2	4	3
71	54	unmarried	less than 1,000,000 JPY	no	0	0	0	0	0	0	0	0	1	1	1	1	1	1	1	1	1	1	1	1	1	1	1	1	1
72	77	married	3,000,000-4,000,000 JPY	no	3	3	3	5	5	5	3	6	3	4	2	3	3	3	2	2	3	3	3	4	3	3	3	4	4
73	65	married	9,000,000-10,000,000 JPY	yes	1	2	0	0	1	0	1	4	1	1	1	1	1	1	1	1	1	1	1	2	1	1	2	2	2
74	51	married	10,000,000-12,000,000 JPY	no	0	0	0	0	0	0	0	1	1	1	1	1	1	1	1	1	1	1	1	2	1	1	1	1	1
75	69	married	20,000,000 or more JPY	yes	1	4	1	2	2	1	3	5	3	2	4	4	4	2	3	3	3	4	2	4	2	2	3	3	3
76	76	married	4,000,000-5,000,000 JPY	yes	0	0	0	0	0	0	0	0	1	1	1	1	1	1	1	1	1	1	1	1	1	1	2	2	3
77	73	married	4,000,000-5,000,000 JPY	no	0	1	0	0	0	0	1	3	1	2	2	1	1	1	1	1	1	1	1	1	1	1	1	1	1
78	71	married	3,000,000-4,000,000 JPY	yes	2	2	0	0	1	1	3	3	1	1	1	3	2	2	2	1	2	1	2	3	1	1	3	4	4
79	70	married	5,000,000-6,000,000 JPY	yes	5	5	5	5	5	5	5	5	3	4	1	3	3	2	2	2	2	2	2	2	2	2	5	5	5
80	74	married	7,000,000-8,000,000 JPY	yes	0	4	0	0	0	1	0	3	1	2	1	1	2	1	1	1	1	1	1	1	1	1	2	2	2
81	70	married	20,000,000 or more JPY	yes	4	3	3	1	5	3	3	5	3	1	1	1	2	1	1	1	3	3	1	3	2	1	2	3	3
82	73	married	7,000,000-8,000,000 JPY	yes	1	0	1	0	2	0	1	2	1	1	1	1	2	1	1	1	1	1	1	3	1	2	2	2	2
83	61	married	5,000,000-6,000,000 JPY	yes	0	3	0	1	0	0	2	3	1	1	1	1	1	1	1	1	1	2	1	1	1	1	1	1	1
84	49	married	10,000,000-12,000,000 JPY	yes	1	2	1	2	1	3	3	2	3	4	2	3	3	2	3	1	4	2	4	3	3	2	4	2	3
85	74	married	4,000,000-5,000,000 JPY	yes	3	4	2	0	5	1	2	4	1	2	1	3	1	1	1	1	2	2	1	3	1	2	2	1	2
86	45	married	6,000,000-7,000,000 JPY	yes	0	0	0	0	0	0	0	3	2	2	3	2	2	3	2	3	2	2	2	2	2	2	2	2	2
87	46	married	10,000,000-12,000,000 JPY	yes	0	1	0	0	0	0	5	2	1	1	2	1	3	1	1	1	1	1	1	3	1	1	1	1	1
88	52	unmarried	5,000,000-6,000,000 JPY	no	0	0	0	0	0	0	1	2	1	1	1	1	1	1	1	1	1	1	1	1	1	1	1	1	1
89	64	unmarried	2,000,000-3,000,000 JPY	yes	3	0	1	1	1	1	1	3	3	3	2	3	2	2	2	1	2	2	2	5	4	3	4	4	4
90	78	married	5,000,000-6,000,000 JPY	yes	2	2	1	2	0	0	3	4	2	2	1	2	3	2	2	2	3	3	3	4	2	2	3	3	3
91	69	married	less than 1,000,000 JPY	yes	1	1	1	1	1	1	1	3	3	2	3	3	2	3	3	2	2	3	3	2	2	3	3	2	3
92	49	married	7,000,000-8,000,000 JPY	yes	0	0	0	0	0	0	0	3	1	1	1	1	1	1	1	1	1	1	1	1	1	1	1	1	1
93	43	married	12,000,000-15,000,000 JPY	yes	3	5	4	4	3	3	2	5	2	2	1	3	3	2	2	2	3	4	3	4	2	2	3	2	4
94	73	married	3,000,000-4,000,000 JPY	yes	5	0	0	0	0	0	1	3	1	2	1	1	2	1	1	1	2	2	1	2	1	1	3	3	3
95	72	married	3,000,000-4,000,000 JPY	yes	0	1	0	0	5	0	0	2	1	3	1	1	1	1	1	1	1	2	1	3	2	1	1	3	3
96	45	unmarried	3,000,000-4,000,000 JPY	no	0	5	0	0	1	0	0	1	1	2	1	2	3	1	1	1	3	3	1	3	2	1	2	2	2
97	40	unmarried	3,000,000-4,000,000 JPY	no	0	1	0	1	1	0	1	3	2	3	1	2	2	2	1	1	3	2	2	2	1	1	1	1	1
98	71	married	5,000,000-6,000,000 JPY	yes	0	0	0	0	1	0	1	2	2	1	2	2	2	2	1	1	2	2	1	3	2	2	3	3	3
99	73	married	3,000,000-4,000,000 JPY	yes	5	3	3	5	5	4	2	5	3	3	3	2	3	4	2	1	3	4	1	4	4	3	5	5	5
100	73	married	4,000,000-5,000,000 JPY	yes	4	3	5	1	5	4	3	5	3	1	1	1	3	1	2	1	3	3	1	4	2	1	3	5	3
101	60	married	6,000,000-7,000,000 JPY	yes	5	4	4	4	5	4	1	5	3	2	1	1	2	1	1	1	1	1	1	2	1	1	1	3	1
102	79	unmarried	less than 1,000,000 JPY	no	1	4	3	2	5	1	2	3	2	2	1	2	2	1	1	1	2	4	1	3	1	2	4	4	2
103	58	married	6,000,000-7,000,000 JPY	yes	1	1	1	1	1	1	0	3	2	1	1	1	2	1	1	1	2	2	2	3	1	2	2	2	2
104	73	unmarried	12,000,000-15,000,000 JPY	no	1	1	0	1	2	0	5	5	2	1	1	2	2	1	1	1	2	2	1	2	1	1	2	3	2
105	70	married	4,000,000-5,000,000 JPY	no	1	1	0	1	1	0	0	3	2	1	1	1	2	1	1	1	1	1	1	2	2	1	1	1	1
106	45	unmarried	2,000,000-3,000,000 JPY	no	1	0	3	5	5	3	1	4	3	2	2	4	3	3	3	4	3	2	4	4	3	3	3	2	2
107	51	unmarried	less than 1,000,000 JPY	no	0	0	0	0	0	2	0	2	2	3	2	3	3	2	3	2	2	2	2	2	2	2	2	2	2
108	58	unmarried	5,000,000-6,000,000 JPY	no	2	3	1	0	2	1	0	5	2	2	1	1	2	1	1	1	2	2	1	2	1	1	2	2	1
109	61	married	6,000,000-7,000,000 JPY	yes	0	1	0	0	0	0	0	2	1	2	1	1	1	1	1	1	1	1	1	1	1	1	1	3	1
110	78	married	12,000,000-15,000,000 JPY	yes	0	0	0	0	0	0	0	2	1	1	1	1	1	1	1	1	1	2	1	2	1	1	2	2	1
111	73	married	3,000,000-4,000,000 JPY	yes	0	0	0	0	0	0	3	3	1	1	1	1	1	1	1	1	1	1	1	1	1	1	1	1	1
112	70	married	4,000,000-5,000,000 JPY	yes	0	1	1	1	1	1	1	2	1	1	1	1	1	1	1	1	1	1	1	2	1	1	2	2	2
113	54	married	6,000,000-7,000,000 JPY	yes	0	0	0	0	0	0	0	2	2	2	1	1	2	1	1	1	2	2	1	2	1	1	2	2	2
114	50	unmarried	5,000,000-6,000,000 JPY	no	1	1	0	0	0	0	1	0	3	3	2	3	2	2	4	4	1	1	4	4	1	1	4	2	2
115	47	married	12,000,000-15,000,000 JPY	yes	1	1	0	1	1	1	1	3	3	4	5	4	4	4	4	3	4	3	3	4	3	3	3	4	3

116	44	unmarried	5,000,000-6,000,000 JPY	no	0	2	0	3	0	0	1	2	3	4	1	2	2	1	1	2	2	2	3	4	2	1	1	1	1
117	63	unmarried	2,000,000-3,000,000 JPY	no	0	0	0	0	0	0	1	2	2	2	2	2	2	2	2	2	2	2	2	2	2	2	2	2	2
118	56	married	7,000,000-8,000,000 JPY	yes	1	1	1	1	1	1	0	4	2	1	1	2	2	2	2	2	2	2	2	3	1	1	2	3	2
119	61	married	3,000,000-4,000,000 JPY	yes	5	5	5	5	5	5	3	5	3	3	2	3	2	2	2	1	2	2	2	3	2	1	4	4	4
120	57	married	12,000,000-15,000,000 JPY	yes	5	5	3	3	2	3	1	5	3	3	3	3	3	3	3	3	4	4	3	4	4	2	4	2	2
121	70	married	4,000,000-5,000,000 JPY	yes	3	1	1	1	2	0	0	2	1	2	2	2	1	3	2	2	2	3	1	3	1	3	4	4	4
122	55	married	4,000,000-5,000,000 JPY	yes	0	1	1	0	1	0	1	3	4	2	2	4	3	3	4	5	4	4	4	5	4	1	3	3	3
123	47	unmarried	6,000,000-7,000,000 JPY	no	5	3	3	3	3	5	5	6	2	2	3	2	2	2	3	3	2	2	1	1	1	5	2	3	4
124	66	married	6,000,000-7,000,000 JPY	yes	1	1	1	1	2	1	1	3	2	2	1	3	3	2	1	1	3	3	3	4	2	3	4	4	2
125	106	married	less than 1,000,000 JPY	no	0	0	0	0	0	0	0	1	1	1	1	1	1	1	1	1	1	1	1	1	1	1	1	1	1
126	81	married	less than 1,000,000 JPY	yes	1	0	0	0	1	1	0	0	1	3	1	1	3	1	1	1	2	3	1	3	2	1	3	2	2
127	70	married	9,000,000-10,000,000 JPY	yes	0	0	0	0	0	0	1	1	1	3	1	1	1	1	1	1	1	2	1	1	1	1	1	1	1
128	69	married	3,000,000-4,000,000 JPY	yes	0	1	0	0	5	1	0	3	2	2	1	3	3	1	1	1	3	4	1	4	1	1	4	4	4
129	48	married	7,000,000-8,000,000 JPY	yes	3	3	3	1	5	2	3	5	4	3	3	4	4	4	4	2	3	2	3	3	2	3	4	3	4
130	72	married	1,000,000-2,000,000 JPY	no	0	1	0	0	0	0	1	2	1	2	1	1	2	1	1	1	1	2	1	2	1	1	3	3	3
131	42	unmarried	5,000,000-6,000,000 JPY	no	1	1	0	0	1	1	2	3	1	1	1	2	1	2	1	1	2	2	1	2	1	1	1	2	1
132	67	married	4,000,000-5,000,000 JPY	yes	1	0	1	0	1	0	1	2	1	2	1	1	1	1	1	1	1	1	1	3	1	1	1	1	2
133	41	unmarried	8,000,000-9,000,000 JPY	yes	1	1	1	0	1	1	0	1	1	1	1	2	1	1	1	1	1	1	1	3	1	1	1	1	1
134	71	married	2,000,000-3,000,000 JPY	yes	0	1	1	0	1	0	0	1	1	1	1	1	1	2	1	1	2	2	1	2	1	1	2	2	1
135	64	married	2,000,000-3,000,000 JPY	yes	0	0	0	0	0	0	1	2	1	1	1	1	1	1	1	1	1	1	1	1	1	1	1	1	1
136	72	married	3,000,000-4,000,000 JPY	yes	1	2	3	3	4	4	1	5	3	3	1	1	3	1	2	2	2	2	2	5	3	3	5	5	5
137	67	unmarried	1,000,000-2,000,000 JPY	no	0	5	5	5	5	5	1	4	1	1	1	1	2	1	1	1	1	1	1	2	1	1	2	5	5
138	61	married	4,000,000-5,000,000 JPY	no	1	1	1	0	1	1	0	4	2	1	1	1	1	1	1	1	2	1	1	2	1	1	3	5	4
139	45	unmarried	1,000,000-2,000,000 JPY	no	0	1	0	0	2	0	0	3	2	2	1	2	1	1	1	1	2	2	2	2	2	1	2	4	1
140	45	unmarried	3,000,000-4,000,000 JPY	no	0	1	0	0	0	0	0	2	1	1	1	2	1	1	1	1	1	1	1	2	1	1	1	1	1
141	67	unmarried	7,000,000-8,000,000 JPY	yes	1	1	0	5	0	0	1	4	2	1	1	2	1	1	1	1	1	1	1	1	1	1	1	3	2
142	80	married	3,000,000-4,000,000 JPY	yes	0	1	0	0	0	0	1	4	1	1	1	1	1	1	1	1	1	2	1	2	1	1	2	2	2
143	72	married	5,000,000-6,000,000 JPY	yes	0	1	0	0	0	0	0	3	1	2	1	1	1	1	1	1	1	1	1	2	1	2	2	2	2
144	71	married	2,000,000-3,000,000 JPY	yes	1	2	1	1	1	1	2	4	3	3	2	3	3	2	2	2	2	2	2	2	2	1	2	2	2
145	72	married	less than 1,000,000 JPY	yes	0	4	0	0	0	0	2	3	1	1	1	1	1	1	1	1	1	2	1	1	1	1	2	2	2
146	54	unmarried	6,000,000-7,000,000 JPY	no	1	1	1	0	1	0	1	3	1	3	1	2	1	2	1	1	1	2	1	1	1	1	1	1	1
147	47	unmarried	4,000,000-5,000,000 JPY	no	3	3	0	3	0	0	1	3	3	1	1	1	1	3	3	3	2	3	2	1	3	3	3	2	1
148	71	married	1,000,000-2,000,000 JPY	yes	2	2	3	3	3	1	1	3	2	2	1	2	3	2	2	1	2	2	1	2	2	1	4	4	4
149	63	married	5,000,000-6,000,000 JPY	no	2	2	5	1	0	0	2	4	3	4	3	3	4	4	3	3	4	5	4	3	2	2	4	4	4
150	45	unmarried	6,000,000-7,000,000 JPY	no	1	1	1	0	1	0	2	3	2	2	1	2	2	1	2	2	1	2	1	3	1	1	1	2	1
151	51	unmarried	6,000,000-7,000,000 JPY	no	0	0	0	0	1	0	1	2	1	1	1	2	1	1	1	1	1	1	1	2	1	1	1	1	1
152	54	unmarried	4,000,000-5,000,000 JPY	yes	0	0	0	0	0	0	1	1	3	1	1	3	2	1	1	1	2	2	1	2	1	1	2	5	5
153	76	married	2,000,000-3,000,000 JPY	no	4	3	3	4	5	5	3	6	3	1	2	2	3	3	2	2	3	3	3	3	3	3	5	5	5
154	69	married	3,000,000-4,000,000 JPY	yes	3	0	0	1	2	0	0	2	2	1	1	2	1	1	1	1	1	2	1	2	1	1	4	3	2
155	81	married	2,000,000-3,000,000 JPY	yes	0	0	0	0	1	0	4	2	1	2	1	1	1	2	1	1	1	2	1	2	1	1	2	3	3
156	60	unmarried	1,000,000-2,000,000 JPY	no	0	1	0	0	0	0	0	2	1	2	1	1	1	1	1	1	1	1	1	1	1	1	2	3	3
157	73	married	4,000,000-5,000,000 JPY	yes	1	0	0	0	0	0	2	3	2	1	1	1	1	2	2	1	2	2	2	2	2	1	2	3	3
158	61	married	4,000,000-5,000,000 JPY	yes	0	1	0	0	0	0	1	4	2	2	1	1	1	1	1	1	1	1	1	1	1	1	1	1	1
159	82	married	2,000,000-3,000,000 JPY	yes	5	4	3	2	1	0	2	6	1	2	3	4	5	5	4	3	2	1	1	2	3	4	5	5	4
160	53	unmarried	4,000,000-5,000,000 JPY	no	0	0	0	0	0	0	0	0	1	1	1	1	1	1	1	1	1	1	1	1	1	1	1	1	1
161	69	married	5,000,000-6,000,000 JPY	yes	3	4	5	5	5	4	1	4	1	2	1	2	2	2	1	1	1	2	1	2	1	1	2	3	2
162	48	married	8,000,000-9,000,000 JPY	yes	3	1	1	0	1	1	1	3	3	3	2	4	4	3	3	1	2	2	1	3	1	2	2	2	3
163	41	unmarried	5,000,000-6,000,000 JPY	yes	1	0	0	0	1	1	1	3	1	2	1	2	3	3	3	2	2	2	2	3	3	2	2	2	2
164	71	unmarried	less than 1,000,000 JPY	no	4	4	3	2	1	0	2	4	3	2	2	1	2	2	1	2	2	2	2	3	2	1	2	2	2
165	70	married	4,000,000-5,000,000 JPY	yes	2	2	2	1	1	0	1	4	1	2	3	2	2	1	1	1	1	2	1	4	1	1	3	2	4
166	45	married	10,000,000-12,000,000 JPY	yes	5	5	5	3	5	0	3	3	4	3	4	5	5	3	5	3	5	5	4	4	3	2	5	5	5
167	53	married	6,000,000-7,000,000 JPY	no	0	1	4	0	0	0	0	3	1	1	1	1	2	2	1	1	1	1	1	2	1	1	2	2	2
168	45	married	4,000,000-5,000,000 JPY	yes	0	1	2	1	0	0	1	2	2	2	1	3	2	2	1	1	2	1	1	3	1	1	1	1	1
169	44	married	9,000,000-10,000,000 JPY	yes	2	2	2	4	0	0	1	5	4	3	3	2	1	2	2	1	1	2	3	2	3	1	1	3	1
170	71	married	5,000,000-6,000,000 JPY	yes	0	0	0	0	0	0	2	2	2	3	2	4	3	2	2	4	2	2	3	3	2	2	3	3	3
171	57	married	10,000,000-12,000,000 JPY	yes	0	0	0	0	0	0	0	1	1	1	1	2	1	1	1	1	2	2	1	2	1	1	2	3	2
172	44	unmarried	7,000,000-8,000,000 JPY	no	0	0	0	0	0	0	0	1	1	2	3	1	1	2	2	1	1	1	1	1	1	1	1	1	1
173	47	married	4,000,000-5,000,000 JPY	yes	0	0	0	0	0	0	0	0	2	1	1	1	3	2	2	1	3	3	2	3	1	1	3	3	3

174	75	married	7,000,000-8,000,000 JPY	yes	0	0	0	0	0	0	0	3	3	3	3	3	3	3	3	3	3	3	3	3	3	3	3	3	3
175	40	married	4,000,000-5,000,000 JPY	yes	0	1	0	1	0	0	0	0	1	1	1	1	1	1	1	1	1	1	1	2	1	1	2	1	1
176	66	married	9,000,000-10,000,000 JPY	yes	0	0	0	0	5	0	1	5	1	3	1	1	2	2	2	1	2	2	1	2	1	1	1	1	1
177	71	married	5,000,000-6,000,000 JPY	yes	1	1	1	1	1	0	1	4	1	1	1	1	1	1	1	1	1	2	1	3	1	1	3	5	4
178	48	unmarried	5,000,000-6,000,000 JPY	no	0	0	0	0	0	0	0	0	2	3	3	1	2	1	1	1	2	2	1	1	1	1	1	1	1
179	71	married	12,000,000-15,000,000 JPY	yes	0	0	1	0	0	0	0	1	1	1	1	2	1	1	1	1	1	2	1	2	1	1	1	1	1
180	60	unmarried	less than 1,000,000 JPY	no	1	2	3	0	1	1	2	3	2	1	1	1	1	1	1	1	1	2	1	2	1	1	1	2	1
181	43	unmarried	3,000,000-4,000,000 JPY	no	3	3	5	0	5	3	1	4	4	2	3	4	4	3	3	3	4	3	4	5	4	1	3	4	3
182	59	married	3,000,000-4,000,000 JPY	yes	0	1	0	1	0	0	1	3	1	2	1	3	2	1	1	1	1	2	1	1	1	1	3	1	1
183	66	married	4,000,000-5,000,000 JPY	yes	1	5	1	2	4	0	3	4	2	3	1	3	1	1	1	1	2	2	1	3	2	2	2	3	3
184	53	unmarried	4,000,000-5,000,000 JPY	yes	1	1	1	1	1	1	1	3	2	1	1	1	2	2	2	2	2	2	2	3	1	1	1	2	1
185	41	unmarried	3,000,000-4,000,000 JPY	yes	0	0	0	1	0	0	1	1	1	1	1	1	2	1	1	1	1	1	1	3	1	1	1	4	1
186	63	married	8,000,000-9,000,000 JPY	yes	0	1	0	1	0	0	0	2	1	1	1	1	1	1	1	1	1	1	1	1	1	1	1	1	1
187	52	married	12,000,000-15,000,000 JPY	yes	1	0	0	0	0	0	0	2	1	1	1	2	1	1	1	1	1	2	1	2	1	1	2	1	2
188	48	married	5,000,000-6,000,000 JPY	no	1	1	1	3	1	1	1	3	2	2	1	1	2	2	2	2	3	3	2	4	2	4	3	3	3
189	56	married	5,000,000-6,000,000 JPY	yes	0	0	0	0	0	0	1	3	1	1	1	1	1	1	1	1	1	1	1	1	1	1	1	1	1
190	79	married	4,000,000-5,000,000 JPY	yes	4	4	4	4	4	2	2	5	4	4	1	2	2	3	3	2	3	3	4	4	4	1	2	5	4
191	78	married	3,000,000-4,000,000 JPY	yes	0	3	0	0	0	0	4	4	3	2	1	1	3	1	2	2	1	3	1	3	1	1	3	2	3
192	46	unmarried	20,000,000 or more JPY	no	0	0	0	0	1	0	1	2	1	1	1	2	2	1	1	1	1	2	1	1	1	1	1	1	1
193	50	married	2,000,000-3,000,000 JPY	yes	1	2	1	0	1	0	0	1	1	1	1	1	2	2	3	2	2	1	2	2	1	1	2	3	1
194	58	unmarried	4,000,000-5,000,000 JPY	no	2	3	0	0	0	0	1	3	3	2	2	3	3	3	3	3	2	2	2	3	2	1	3	3	3
195	52	married	20,000,000 or more JPY	yes	0	0	0	0	0	0	0	3	1	1	1	1	1	1	1	1	1	1	1	1	1	1	1	1	1
196	50	married	3,000,000-4,000,000 JPY	yes	0	0	0	0	0	0	2	2	1	2	1	1	1	1	1	1	1	1	1	2	1	1	1	1	1
197	47	unmarried	3,000,000-4,000,000 JPY	no	0	0	0	0	0	0	0	0	1	1	1	1	1	1	1	1	1	1	1	1	1	1	1	1	1
198	51	unmarried	3,000,000-4,000,000 JPY	no	0	0	0	0	0	0	0	2	1	1	1	1	1	1	1	1	1	1	1	1	1	1	1	1	1
199	62	unmarried	less than 1,000,000 JPY	no	1	2	0	1	0	0	1	5	1	1	1	2	1	1	1	1	1	1	1	1	1	1	1	1	1
200	67	married	5,000,000-6,000,000 JPY	yes	0	0	0	0	5	0	1	3	4	4	1	3	3	3	3	2	4	4	3	5	4	2	4	5	4
201	51	married	7,000,000-8,000,000 JPY	yes	0	1	0	0	0	0	1	3	1	3	1	3	2	2	1	1	1	2	1	4	3	2	4	4	3
202	61	unmarried	less than 1,000,000 JPY	no	0	5	0	2	0	0	3	3	3	2	1	1	4	2	2	2	4	5	2	3	3	1	5	5	4
203	80	married	3,000,000-4,000,000 JPY	no	1	2	1	1	1	1	2	3	2	2	1	2	2	2	1	1	3	4	1	3	1	2	5	5	4
204	46	married	7,000,000-8,000,000 JPY	yes	2	2	2	3	3	1	2	4	2	2	1	3	1	2	1	1	1	2	1	3	1	2	2	2	2
205	73	married	10,000,000-12,000,000 JPY	yes	0	0	0	0	0	0	0	2	1	2	1	1	2	1	1	1	1	2	1	2	1	1	1	1	1
206	71	married	2,000,000-3,000,000 JPY	yes	0	1	0	1	0	0	2	3	2	2	1	4	2	2	1	1	2	3	2	3	2	1	2	2	2
207	70	married	10,000,000-12,000,000 JPY	yes	0	1	0	1	1	0	1	3	1	1	1	1	2	2	1	1	2	2	1	2	1	1	1	1	2
208	70	married	7,000,000-8,000,000 JPY	yes	0	0	0	1	1	1	1	3	3	4	1	1	2	1	1	1	2	2	1	2	3	1	3	3	2
209	45	married	5,000,000-6,000,000 JPY	yes	1	1	0	0	1	0	0	3	3	3	2	2	1	2	2	2	2	2	1	4	2	1	5	5	5
210	57	unmarried	less than 1,000,000 JPY	no	1	1	1	1	1	0	1	3	3	3	3	3	3	3	3	3	3	3	3	1	3	3	3	3	3
211	59	married	9,000,000-10,000,000 JPY	yes	0	0	0	0	0	0	1	3	1	1	2	2	2	1	1	1	1	1	1	1	1	1	1	1	2
212	70	married	8,000,000-9,000,000 JPY	yes	0	0	0	1	0	0	1	3	1	1	1	2	1	1	1	1	1	1	1	3	1	1	1	2	2
213	73	married	4,000,000-5,000,000 JPY	yes	4	4	0	5	5	0	3	3	3	3	3	5	5	3	3	3	3	1	3	3	3	3	5	5	5
214	61	married	12,000,000-15,000,000 JPY	yes	0	1	0	0	0	0	5	5	1	1	1	2	2	2	2	2	2	2	2	2	2	2	3	3	3
215	59	married	6,000,000-7,000,000 JPY	yes	5	2	5	5	5	4	5	3	3	1	1	2	3	2	1	2	3	2	3	4	1	1	1	1	1
216	58	married	10,000,000-12,000,000 JPY	no	0	0	5	0	0	0	1	3	3	1	1	2	2	2	1	1	2	2	2	4	1	1	2	1	2
217	46	unmarried	3,000,000-4,000,000 JPY	no	0	0	0	0	0	0	0	0	2	2	1	2	2	2	2	1	2	2	2	3	2	1	1	1	1
218	59	married	10,000,000-12,000,000 JPY	yes	0	1	0	0	0	0	0	3	1	1	1	1	2	1	1	1	1	2	1	2	1	1	1	1	1
219	44	married	5,000,000-6,000,000 JPY	yes	0	0	0	0	0	0	0	1	1	1	1	1	1	1	1	1	1	1	1	1	1	1	1	2	1
220	41	married	8,000,000-9,000,000 JPY	yes	0	0	0	0	0	0	0	0	1	1	1	1	1	1	1	1	1	1	1	1	1	1	1	1	1
221	73	married	10,000,000-12,000,000 JPY	yes	0	0	0	0	1	0	0	2	2	1	1	1	2	1	1	1	2	2	1	2	1	2	2	1	1
222	106	unmarried	20,000,000 or more JPY	no	3	1	0	1	1	0	1	4	3	4	1	3	4	5	5	1	3	3	1	5	5	1	1	5	1
223	54	unmarried	less than 1,000,000 JPY	no	0	0	0	0	0	0	1	0	2	1	1	2	1	1	1	1	1	2	1	1	1	1	2	2	2
224	72	married	3,000,000-4,000,000 JPY	yes	0	1	0	0	0	0	2	3	1	1	1	1	1	1	1	1	1	2	1	2	1	1	2	1	2
225	68	married	3,000,000-4,000,000 JPY	yes	1	1	0	0	0	0	5	2	1	1	1	1	1	1	1	1	1	1	1	1	1	1	2	2	2
226	70	married	2,000,000-3,000,000 JPY	yes	0	2	0	0	0	0	3	4	1	1	1	1	1	1	1	1	1	2	1	1	1	1	1	1	1
227	74	married	2,000,000-3,000,000 JPY	yes	5	5	1	0	0	0	5	5	3	3	3	3	3	3	3	3	3	3	3	3	3	3	3	3	3
228	60	unmarried	7,000,000-8,000,000 JPY	no	0	0	0	0	0	0	0	3	1	1	1	1	1	1	1	1	1	1	1	1	1	1	1	1	1
229	47	married	7,000,000-8,000,000 JPY	yes	0	0	0	0	0	0	0	3	1	1	1	1	1	1	1	1	1	1	1	1	1	1	1	1	1
230	42	married	10,000,000-12,000,000 JPY	no	0	0	0	0	0	0	1	2	2	1	1	2	3	2	2	2	2	2	3	3	2	1	3	3	4
231	66	married	10,000,000-12,000,000 JPY	yes	3	1	2	0	5	0	1	4	1	2	1	2	2	1	1	1	1	3	1	2	1	1	1	2	1

232	41	married	4,000,000-5,000,000 JPY	yes	0	0	0	0	0	0	0	6	1	1	1	1	2	1	1	1	1	1	1	1	1	1	1	2	1
233	53	married	12,000,000-15,000,000 JPY	no	0	0	0	0	3	5	1	2	1	1	1	1	2	1	1	1	1	2	1	3	1	1	2	2	1
234	55	married	5,000,000-6,000,000 JPY	yes	0	1	0	1	2	0	1	3	2	3	2	1	2	2	1	1	2	3	2	4	2	1	1	3	2
235	71	married	9,000,000-10,000,000 JPY	yes	0	3	5	3	5	1	1	5	2	4	4	2	3	2	2	2	2	2	2	2	2	2	2	2	2
236	50	unmarried	5,000,000-6,000,000 JPY	no	3	1	0	0	2	0	0	4	3	1	1	3	2	1	1	1	2	2	2	3	3	2	2	3	2
237	80	married	4,000,000-5,000,000 JPY	yes	5	4	5	0	5	5	2	5	3	1	1	4	4	4	4	4	4	4	4	5	3	3	5	5	5
238	70	married	3,000,000-4,000,000 JPY	yes	2	3	0	2	2	1	2	4	2	2	1	2	3	2	3	2	2	2	2	2	1	1	2	4	2
239	46	unmarried	9,000,000-10,000,000 JPY	no	0	1	0	0	0	0	0	1	1	1	1	2	2	1	1	1	2	1	1	3	1	1	1	2	1
240	65	married	less than 1,000,000 JPY	no	0	0	0	0	0	0	2	3	3	3	3	3	3	3	3	3	3	3	3	2	3	3	3	3	3
241	53	unmarried	8,000,000-9,000,000 JPY	no	0	0	0	0	0	0	1	3	3	3	3	3	2	3	2	3	2	2	3	2	3	3	2	2	3
242	54	unmarried	3,000,000-4,000,000 JPY	no	0	0	0	0	0	0	4	2	2	2	1	2	3	2	1	1	2	1	1	2	1	1	1	2	1
243	46	unmarried	6,000,000-7,000,000 JPY	no	1	2	3	0	4	2	2	5	3	2	1	2	3	1	1	1	2	2	2	2	3	2	2	2	2
244	70	married	2,000,000-3,000,000 JPY	no	1	1	1	1	5	0	2	4	1	2	1	3	2	1	1	1	2	2	1	2	1	1	2	2	2
245	72	married	2,000,000-3,000,000 JPY	yes	3	4	4	4	4	0	1	3	2	2	2	1	2	2	2	1	2	2	2	2	2	3	2	3	3
246	67	married	8,000,000-9,000,000 JPY	no	1	1	0	0	0	0	2	2	2	2	1	1	1	1	2	1	2	1	1	1	1	1	2	3	2
247	63	married	2,000,000-3,000,000 JPY	yes	1	2	0	0	1	2	1	3	1	3	1	2	1	1	1	1	2	2	1	2	1	1	1	3	1
248	56	married	4,000,000-5,000,000 JPY	no	5	5	5	0	0	0	1	3	1	1	1	3	2	2	2	2	2	3	2	3	2	1	3	1	1
249	47	unmarried	8,000,000-9,000,000 JPY	no	1	1	0	0	1	0	1	2	2	2	2	3	2	1	1	2	2	2	2	3	2	1	2	2	1
250	61	married	less than 1,000,000 JPY	no	4	4	4	1	5	1	2	3	3	1	2	2	2	2	2	2	2	2	3	4	3	1	4	4	3
251	76	married	6,000,000-7,000,000 JPY	yes	1	2	2	2	3	3	1	4	3	2	1	2	2	1	1	1	2	3	1	4	2	1	4	4	4
252	42	married	2,000,000-3,000,000 JPY	yes	2	2	2	2	2	2	4	4	3	3	3	3	3	3	3	3	3	3	3	3	3	3	3	3	3
253	72	married	9,000,000-10,000,000 JPY	yes	0	0	0	0	0	0	1	1	1	1	1	1	1	1	1	1	1	1	1	1	1	1	1	1	1
254	52	married	8,000,000-9,000,000 JPY	yes	1	2	0	0	5	0	1	3	2	1	2	3	3	2	2	2	2	2	2	3	1	1	1	2	2
255	73	married	1,000,000-2,000,000 JPY	yes	0	2	0	4	1	0	1	3	2	3	3	2	3	2	2	1	3	3	3	3	2	3	4	4	2
256	47	married	4,000,000-5,000,000 JPY	no	0	1	0	0	0	0	0	1	1	1	2	2	2	3	2	1	2	2	2	3	2	1	2	1	1
257	50	married	4,000,000-5,000,000 JPY	yes	0	1	0	0	1	0	0	3	1	1	1	1	3	1	1	1	1	3	1	3	1	1	2	2	3
258	58	married	6,000,000-7,000,000 JPY	yes	1	1	1	0	1	0	0	3	1	3	1	2	1	1	1	1	2	2	1	2	1	1	3	3	1
259	70	married	1,000,000-2,000,000 JPY	yes	0	1	0	1	0	0	2	3	2	3	1	1	1	2	1	1	1	3	1	3	1	3	2	2	2
260	59	married	12,000,000-15,000,000 JPY	yes	0	1	0	0	3	0	2	5	1	1	1	2	1	1	1	1	1	2	2	2	1	1	2	3	2
261	49	unmarried	20,000,000 or more JPY	no	2	3	1	0	1	0	1	2	1	1	1	3	2	4	2	1	1	2	3	4	2	2	3	1	2
262	72	married	4,000,000-5,000,000 JPY	yes	0	1	1	1	2	0	0	2	2	4	1	2	2	2	1	1	4	3	2	4	2	4	4	4	4
263	71	married	3,000,000-4,000,000 JPY	yes	0	1	0	0	1	0	1	3	1	1	1	1	2	1	1	1	1	1	1	1	1	1	1	2	1
264	46	married	12,000,000-15,000,000 JPY	no	0	1	0	0	0	0	0	3	1	1	1	1	1	1	1	1	1	1	1	1	1	1	1	1	1
265	70	married	2,000,000-3,000,000 JPY	no	1	4	1	1	4	0	0	3	1	2	1	1	1	2	2	1	1	3	2	3	1	1	3	3	3
266	40	unmarried	5,000,000-6,000,000 JPY	no	0	0	0	0	0	0	0	3	3	3	3	3	3	3	3	3	3	3	3	3	3	3	3	3	3
267	70	married	4,000,000-5,000,000 JPY	yes	0	1	0	0	0	0	1	2	1	1	1	1	2	1	1	1	1	2	1	1	1	1	1	1	1
268	73	unmarried	3,000,000-4,000,000 JPY	no	0	3	0	3	0	0	2	3	2	2	1	2	2	1	1	1	2	2	2	2	1	2	3	3	3
269	53	married	9,000,000-10,000,000 JPY	yes	0	0	5	0	0	0	1	2	1	1	1	1	1	1	1	1	1	2	1	3	1	2	2	2	2
270	40	unmarried	9,000,000-10,000,000 JPY	no	0	0	0	0	0	0	5	0	1	1	1	1	1	1	1	1	1	1	1	1	1	1	1	1	1
271	66	married	10,000,000-12,000,000 JPY	yes	2	3	0	3	5	5	5	5	3	2	1	3	3	2	2	2	2	3	2	4	2	2	3	1	2
272	41	married	less than 1,000,000 JPY	yes	0	0	0	0	0	0	0	0	1	1	1	1	1	1	1	1	1	1	1	1	1	1	1	1	1
273	74	married	3,000,000-4,000,000 JPY	yes	1	2	0	3	3	0	0	5	4	4	1	4	3	3	2	1	3	3	2	3	1	1	3	4	4
274	42	unmarried	6,000,000-7,000,000 JPY	no	1	0	0	0	0	0	0	3	1	1	1	2	2	2	2	1	2	2	2	2	2	2	2	2	2
275	65	married	7,000,000-8,000,000 JPY	yes	0	1	0	1	1	1	1	5	1	2	1	2	1	1	1	1	2	2	1	3	1	1	1	1	2
276	71	unmarried	3,000,000-4,000,000 JPY	no	1	1	0	2	5	0	1	4	1	1	1	2	2	1	1	1	1	2	1	3	1	1	3	2	2
277	74	married	3,000,000-4,000,000 JPY	yes	5	5	3	3	5	5	2	4	1	1	1	1	2	2	1	1	2	2	1	3	1	1	2	3	2
278	58	married	12,000,000-15,000,000 JPY	yes	1	2	0	1	0	1	1	5	2	2	2	2	2	2	1	1	1	2	1	2	1	1	2	3	3
279	41	married	5,000,000-6,000,000 JPY	yes	0	1	0	0	0	0	1	3	1	2	1	1	1	2	2	1	1	1	1	1	1	1	1	2	1
280	71	married	2,000,000-3,000,000 JPY	yes	1	1	2	2	3	0	1	5	2	2	1	1	2	1	1	1	1	2	1	3	1	1	3	3	3
281	73	unmarried	less than 1,000,000 JPY	no	0	0	0	0	0	0	5	0	1	1	1	1	1	1	1	1	1	1	1	1	1	1	1	1	1
282	49	unmarried	3,000,000-4,000,000 JPY	no	0	0	0	0	0	0	0	0	1	1	1	1	1	1	1	1	1	1	1	2	3	1	1	1	1
283	64	married	3,000,000-4,000,000 JPY	yes	0	0	0	0	0	0	1	2	1	1	1	1	1	1	1	1	1	1	1	1	1	1	1	2	1
284	60	unmarried	9,000,000-10,000,000 JPY	no	0	1	1	1	0	0	1	2	2	2	1	2	2	2	2	1	3	3	2	4	2	1	2	2	2
285	40	married	6,000,000-7,000,000 JPY	yes	1	0	0	0	0	0	0	1	3	1	1	4	4	3	2	1	3	4	1	2	1	1	3	2	2
286	52	unmarried	less than 1,000,000 JPY	no	0	0	0	0	0	0	1	2	3	2	1	3	3	3	2	2	2	2	2	2	1	1	1	1	1
287	61	married	4,000,000-5,000,000 JPY	yes	1	1	1	0	1	1	1	2	1	2	1	1	2	1	1	1	1	2	1	2	1	1	1	1	1
288	57	unmarried	6,000,000-7,000,000 JPY	yes	0	0	0	0	0	0	0	1	1	1	1	2	3	1	2	1	1	4	1	4	1	1	4	4	4
289	56	married	10,000,000-12,000,000 JPY	yes	4	3	4	3	4	4	0	4	2	2	2	1	2	2	1	1	2	3	1	5	2	2	3	4	4

290	73	married	3,000,000-4,000,000 JPY	yes	2	1	1	0	1	1	1	4	1	3	1	1	2	2	2	1	2	3	1	4	1	2	3	3	3
291	43	married	4,000,000-5,000,000 JPY	no	0	0	0	1	0	0	1	3	2	1	2	2	2	2	2	1	2	2	1	2	1	1	1	2	1
292	70	married	10,000,000-12,000,000 JPY	yes	0	2	0	1	0	0	1	4	1	2	1	1	2	1	1	1	1	3	1	3	1	1	1	1	3
293	64	married	less than 1,000,000 JPY	yes	0	0	0	0	0	0	1	2	1	1	1	1	1	1	1	1	1	2	1	1	1	1	1	1	1
294	41	unmarried	6,000,000-7,000,000 JPY	no	1	1	1	0	0	0	1	2	1	1	1	2	2	3	2	2	2	1	4	5	3	1	1	1	1
295	52	married	15,000,000-18,000,000 JPY	no	0	0	0	0	2	0	0	4	2	2	1	2	2	2	2	2	2	2	2	2	2	2	2	2	2
296	49	married	5,000,000-6,000,000 JPY	yes	0	0	0	0	0	0	0	1	1	1	2	1	2	1	2	1	1	2	1	2	1	3	2	2	2
297	50	married	7,000,000-8,000,000 JPY	yes	5	0	1	4	4	0	2	4	1	1	1	1	2	1	1	1	2	2	2	3	1	1	4	5	3
298	50	unmarried	1,000,000-2,000,000 JPY	no	0	2	0	2	0	0	1	3	2	3	2	4	2	5	4	4	4	2	5	4	3	2	2	2	3
299	63	married	3,000,000-4,000,000 JPY	yes	5	5	5	1	5	5	1	5	3	3	2	3	4	3	2	1	2	2	2	4	4	1	5	4	4
300	42	married	7,000,000-8,000,000 JPY	yes	1	1	0	0	2	0	1	5	2	2	1	3	3	2	2	2	2	2	2	3	2	2	3	4	4
301	69	unmarried	less than 1,000,000 JPY	no	2	2	2	3	2	4	4	5	3	3	3	4	4	3	3	3	3	2	3	3	3	2	2	1	2
302	62	married	4,000,000-5,000,000 JPY	yes	0	1	0	0	0	0	1	2	3	2	1	2	3	2	2	1	2	2	1	4	2	4	3	4	1
303	60	unmarried	1,000,000-2,000,000 JPY	no	1	1	0	4	2	0	1	3	2	2	1	2	2	2	2	2	2	2	1	4	2	1	4	5	4
304	72	married	2,000,000-3,000,000 JPY	yes	0	0	0	0	0	0	3	3	1	1	1	1	1	1	1	1	1	1	1	1	1	1	1	1	1
305	74	married	2,000,000-3,000,000 JPY	yes	5	5	0	5	5	5	4	0	1	1	1	1	1	1	1	1	1	2	2	2	1	1	3	4	4
306	70	married	5,000,000-6,000,000 JPY	yes	0	3	0	0	0	0	0	2	3	3	1	1	1	2	1	1	1	2	1	3	1	1	5	5	5
307	71	married	3,000,000-4,000,000 JPY	yes	0	0	1	0	1	1	0	1	1	1	1	1	2	1	1	1	1	2	1	2	1	1	4	4	4
308	66	married	3,000,000-4,000,000 JPY	yes	0	0	0	0	0	0	0	2	1	1	1	1	1	1	1	1	1	3	1	3	1	1	3	1	1
309	52	unmarried	5,000,000-6,000,000 JPY	no	0	0	0	0	0	0	5	1	1	1	1	1	2	1	1	1	1	1	1	2	1	1	1	1	2
310	72	married	2,000,000-3,000,000 JPY	yes	0	5	5	5	5	0	2	6	3	2	2	1	2	1	1	1	1	4	2	1	2	1	1	5	1
311	56	unmarried	9,000,000-10,000,000 JPY	no	4	4	2	1	1	0	1	4	3	3	1	2	2	2	1	1	2	3	1	3	2	1	4	5	2
312	61	married	4,000,000-5,000,000 JPY	yes	1	3	1	1	1	0	1	4	2	2	2	2	2	1	1	1	1	2	1	3	1	1	3	4	2
313	69	married	5,000,000-6,000,000 JPY	yes	0	4	0	0	0	0	5	3	1	1	1	1	1	1	1	1	1	2	1	2	1	1	2	2	2
314	52	married	10,000,000-12,000,000 JPY	yes	1	0	0	0	1	1	1	1	2	3	3	2	1	2	2	1	2	2	1	1	2	2	3	2	2
315	59	married	3,000,000-4,000,000 JPY	no	0	1	0	0	0	0	1	2	3	3	1	2	2	1	1	1	2	2	1	3	2	1	4	5	5
316	69	unmarried	1,000,000-2,000,000 JPY	yes	4	1	2	0	5	0	1	5	2	3	2	3	1	2	3	1	2	3	2	4	2	1	2	2	2
317	53	unmarried	8,000,000-9,000,000 JPY	no	0	0	0	0	0	0	2	4	1	1	1	1	1	1	1	1	1	1	1	1	1	1	1	1	1
318	70	married	3,000,000-4,000,000 JPY	yes	5	5	0	5	5	5	4	5	3	1	1	2	3	4	3	1	1	2	1	3	1	1	1	1	1
319	63	married	less than 1,000,000 JPY	no	0	1	0	1	1	0	1	3	1	1	1	1	2	1	2	1	2	2	1	2	1	1	1	1	1
320	65	married	5,000,000-6,000,000 JPY	yes	1	5	1	0	5	0	2	6	4	3	1	3	3	2	2	1	2	2	1	2	2	2	3	1	5
321	62	married	5,000,000-6,000,000 JPY	no	1	3	0	2	5	0	1	5	1	1	1	3	2	2	1	1	2	2	1	2	2	1	2	3	4
322	54	married	10,000,000-12,000,000 JPY	yes	3	3	1	5	5	5	2	5	3	1	1	4	2	2	3	2	2	1	1	4	1	1	1	1	1
323	45	unmarried	3,000,000-4,000,000 JPY	no	1	1	0	0	0	0	1	3	4	3	2	3	3	1	1	2	2	1	2	1	1	3	2	2	1
324	65	married	7,000,000-8,000,000 JPY	yes	0	2	0	2	2	2	2	3	3	3	3	3	3	3	3	3	3	3	3	3	3	3	3	3	3
325	52	married	8,000,000-9,000,000 JPY	yes	1	1	0	0	0	0	2	4	3	3	1	2	3	1	1	1	1	2	1	3	3	1	3	3	3
326	53	married	4,000,000-5,000,000 JPY	yes	0	4	0	1	0	0	3	3	2	2	1	3	3	3	3	2	2	2	2	2	2	2	2	2	3
327	70	married	8,000,000-9,000,000 JPY	yes	0	0	0	0	0	0	1	3	2	3	1	2	2	3	3	3	2	2	3	4	2	2	3	2	2
328	48	unmarried	6,000,000-7,000,000 JPY	no	0	0	0	0	0	0	0	1	1	1	1	1	1	1	1	1	1	1	1	1	1	1	1	1	1
329	71	married	3,000,000-4,000,000 JPY	yes	0	0	1	1	1	1	0	2	1	2	1	3	2	1	1	1	2	2	1	3	2	1	2	2	2
330	68	married	2,000,000-3,000,000 JPY	yes	0	1	0	5	5	0	1	5	3	1	1	5	4	2	1	1	3	2	1	3	1	1	5	5	2
331	75	married	3,000,000-4,000,000 JPY	yes	5	5	4	4	5	3	2	4	3	1	1	1	2	1	2	1	3	3	2	3	2	2	4	3	3
332	69	married	1,000,000-2,000,000 JPY	yes	0	5	5	5	0	0	1	4	1	1	1	1	1	1	1	1	1	1	1	1	1	1	1	1	1
333	43	unmarried	3,000,000-4,000,000 JPY	no	2	2	2	1	0	1	2	5	3	1	5	5	5	3	4	3	5	2	3	5	2	1	2	2	1
334	63	unmarried	2,000,000-3,000,000 JPY	no	0	2	1	1	0	0	0	3	2	1	1	2	2	1	2	1	2	1	2	2	1	1	2	1	2
335	66	married	2,000,000-3,000,000 JPY	yes	5	4	5	5	5	5	2	6	3	4	3	4	4	4	4	4	4	5	5	5	4	3	5	5	5
336	50	unmarried	less than 1,000,000 JPY	no	0	1	0	1	1	0	0	3	1	1	1	1	1	1	1	1	1	2	1	1	1	1	2	2	1
337	62	married	3,000,000-4,000,000 JPY	yes	2	1	0	1	1	0	2	4	1	2	1	3	2	2	1	1	2	2	1	2	1	1	2	2	2
338	81	married	2,000,000-3,000,000 JPY	no	1	2	3	3	5	5	3	5	2	2	1	2	2	1	1	1	2	1	1	2	1	2	5	5	5
339	40	married	5,000,000-6,000,000 JPY	no	3	2	2	2	0	0	0	0	1	1	1	1	1	1	1	1	1	1	1	1	1	1	1	1	1
340	74	married	4,000,000-5,000,000 JPY	yes	0	0	0	0	5	0	0	2	1	2	1	1	1	1	1	1	2	2	1	2	1	1	3	3	1
341	71	married	3,000,000-4,000,000 JPY	yes	4	1	1	0	1	0	1	3	2	2	2	2	2	2	2	2	2	4	2	2	2	2	2	2	2
342	52	unmarried	1,000,000-2,000,000 JPY	no	1	2	2	3	2	2	0	5	1	2	2	2	1	2	2	2	3	3	2	2	3	2	2	3	2
343	48	unmarried	10,000,000-12,000,000 JPY	yes	0	1	0	0	0	0	1	3	1	1	1	2	2	1	1	1	2	2	1	2	1	2	1	1	1
344	82	married	5,000,000-6,000,000 JPY	yes	1	1	0	1	1	0	1	3	2	2	1	2	2	1	1	1	2	1	1	2	2	1	4	5	1
345	70	married	9,000,000-10,000,000 JPY	yes	0	2	0	3	3	0	2	4	2	1	1	2	2	2	2	1	2	2	1	3	1	1	3	3	3
346	66	married	20,000,000 or more JPY	yes	0	0	0	0	1	1	1	1	1	2	1	1	1	1	1	1	1	2	1	2	1	1	1	1	1
347	66	married	5,000,000-6,000,000 JPY	yes	0	0	0	0	1	0	1	3	2	2	2	2	2	1	1	1	1	2	1	2	1	2	2	2	2

348	59	unmarried	4,000,000-5,000,000 JPY	no	0	0	0	0	0	0	0	0	2	1	1	3	2	1	1	1	2	2	1	1	1	1	2	2	2
349	53	married	3,000,000-4,000,000 JPY	yes	1	3	1	3	2	0	1	5	3	4	3	3	3	4	4	3	4	4	3	5	3	2	5	3	5
350	62	married	less than 1,000,000 JPY	no	3	2	3	1	5	3	1	3	3	2	2	5	4	4	4	5	3	3	3	5	5	3	4	5	5
351	58	married	8,000,000-9,000,000 JPY	yes	1	0	2	1	2	2	2	4	1	1	1	2	2	1	1	1	1	1	1	2	1	1	2	2	2
352	43	married	9,000,000-10,000,000 JPY	yes	0	1	0	0	1	0	1	2	1	2	1	1	2	2	2	1	1	1	1	1	1	1	1	1	1
353	64	married	4,000,000-5,000,000 JPY	yes	3	3	3	4	5	5	2	4	2	1	1	3	3	1	3	1	3	3	2	3	3	1	1	2	1
354	56	married	12,000,000-15,000,000 JPY	yes	1	0	1	0	2	0	1	3	1	2	1	1	1	1	1	1	1	1	1	4	1	1	4	4	3
355	48	unmarried	2,000,000-3,000,000 JPY	no	0	1	1	0	0	0	0	3	1	1	1	1	1	1	1	1	2	2	1	3	1	1	2	2	1
356	52	unmarried	5,000,000-6,000,000 JPY	no	3	3	4	4	4	2	2	6	2	2	4	4	3	4	4	5	4	5	5	5	4	3	5	5	5
357	49	married	10,000,000-12,000,000 JPY	yes	0	1	0	3	2	0	1	3	2	3	3	3	3	4	3	1	2	2	1	4	2	2	2	2	1
358	68	married	10,000,000-12,000,000 JPY	yes	0	0	0	0	0	0	0	0	1	1	1	1	1	1	1	1	1	1	1	1	1	1	1	1	1
359	63	unmarried	1,000,000-2,000,000 JPY	no	1	1	0	1	1	0	1	3	2	2	1	1	2	2	2	1	2	2	2	3	1	2	2	2	2
360	51	married	less than 1,000,000 JPY	no	0	0	0	0	0	0	0	6	1	1	1	1	1	1	1	1	1	1	1	1	1	1	1	1	1
361	42	unmarried	2,000,000-3,000,000 JPY	no	1	0	0	0	0	0	1	2	1	2	1	2	2	1	1	1	1	1	1	1	1	1	1	2	1
362	52	married	10,000,000-12,000,000 JPY	yes	1	0	1	1	1	0	1	1	2	3	2	1	2	2	1	2	2	1	2	2	1	2	3	2	3
363	68	married	2,000,000-3,000,000 JPY	yes	0	0	0	0	0	0	1	0	1	1	1	1	1	1	1	1	1	1	1	1	1	1	1	1	1
364	71	married	2,000,000-3,000,000 JPY	yes	0	0	0	0	0	0	1	3	1	1	2	1	1	1	1	1	1	2	1	2	1	2	2	2	2
365	57	unmarried	4,000,000-5,000,000 JPY	no	0	2	0	0	1	0	1	2	2	1	1	2	2	1	1	1	2	2	1	3	1	1	1	2	2
366	72	married	5,000,000-6,000,000 JPY	yes	1	1	2	1	1	1	2	4	2	2	1	3	2	2	1	2	1	2	1	2	1	1	2	3	1
367	80	unmarried	10,000,000-12,000,000 JPY	yes	1	2	0	1	1	1	0	2	2	2	1	1	2	1	1	1	1	2	2	3	1	1	3	3	2
368	63	married	10,000,000-12,000,000 JPY	yes	1	1	1	1	1	0	1	4	2	2	1	3	3	2	2	1	2	2	2	3	2	1	3	3	3
369	54	unmarried	1,000,000-2,000,000 JPY	no	2	2	0	0	2	0	1	3	3	3	3	3	3	3	3	3	3	3	3	3	3	3	3	4	4
370	56	married	2,000,000-3,000,000 JPY	yes	1	3	3	1	1	1	0	2	2	1	1	1	2	2	1	1	1	2	1	2	1	1	2	1	1
371	68	married	4,000,000-5,000,000 JPY	yes	2	2	2	0	2	2	1	4	1	1	1	1	1	1	1	1	1	1	1	1	1	1	1	1	1
372	61	unmarried	1,000,000-2,000,000 JPY	no	1	4	4	0	5	1	2	5	2	2	1	3	2	2	2	2	2	3	1	4	3	2	2	3	3
373	72	married	3,000,000-4,000,000 JPY	yes	1	1	1	0	1	1	1	3	2	2	2	2	2	2	2	2	2	2	2	2	2	2	2	2	2
374	74	married	2,000,000-3,000,000 JPY	yes	0	0	0	0	1	0	2	2	2	2	1	3	1	1	1	1	2	2	1	3	1	1	5	5	4
375	49	married	8,000,000-9,000,000 JPY	yes	1	1	1	0	2	0	1	4	1	1	1	1	1	1	1	1	2	2	1	2	1	1	2	2	2
376	63	married	5,000,000-6,000,000 JPY	yes	0	0	2	5	2	0	1	3	1	2	3	1	3	2	1	1	3	3	2	2	1	1	2	2	2
377	72	married	2,000,000-3,000,000 JPY	yes	0	0	0	0	0	0	1	4	1	1	1	2	1	1	1	1	1	2	1	2	2	1	2	2	1
378	53	married	10,000,000-12,000,000 JPY	yes	1	2	3	3	0	0	3	6	3	4	4	4	4	4	4	4	4	4	4	4	3	3	5	5	5
379	66	married	12,000,000-15,000,000 JPY	yes	0	0	0	0	0	0	1	3	1	2	1	2	2	2	2	2	2	2	2	2	1	1	2	3	2
380	64	married	12,000,000-15,000,000 JPY	no	0	1	1	1	2	0	2	3	2	1	1	2	2	1	1	1	2	2	1	3	1	2	3	3	3
381	67	married	6,000,000-7,000,000 JPY	yes	1	5	0	1	0	1	3	3	1	1	1	2	1	1	1	1	1	2	1	2	1	1	1	1	1
382	41	unmarried	15,000,000-18,000,000 JPY	no	4	1	2	3	1	3	2	1	3	3	3	3	3	3	3	3	2	3	4	3	4	5	3	1	2
383	52	married	20,000,000 or more JPY	yes	1	4	1	1	1	1	2	4	3	3	2	3	2	1	1	2	2	2	1	4	1	1	3	3	3
384	59	married	1,000,000-2,000,000 JPY	yes	0	0	0	0	0	0	2	5	2	2	2	3	2	2	2	3	2	3	4	4	3	2	3	3	3
385	50	unmarried	1,000,000-2,000,000 JPY	no	1	2	0	0	0	0	1	2	2	2	1	2	2	2	2	1	2	2	2	3	2	2	2	2	2
386	64	married	1,000,000-2,000,000 JPY	yes	0	1	1	0	2	1	1	3	2	3	1	2	2	3	2	1	1	2	2	3	2	1	1	3	3
387	49	married	12,000,000-15,000,000 JPY	yes	4	5	1	0	1	0	0	4	1	2	1	1	2	1	1	1	3	3	1	3	1	1	2	4	2
388	50	unmarried	4,000,000-5,000,000 JPY	no	0	0	0	0	1	0	0	1	1	1	1	2	1	1	1	1	1	1	1	1	1	2	1	1	1
389	53	unmarried	2,000,000-3,000,000 JPY	no	0	0	0	0	0	0	0	0	1	1	2	1	2	1	1	1	1	2	1	2	1	1	1	1	1
390	79	married	3,000,000-4,000,000 JPY	yes	5	5	4	0	5	2	2	5	2	1	1	3	3	1	1	1	1	2	1	3	1	1	1	1	1
391	48	married	8,000,000-9,000,000 JPY	no	0	1	0	1	0	0	2	2	1	1	1	1	1	1	1	1	1	2	1	1	1	1	1	1	1
392	45	unmarried	1,000,000-2,000,000 JPY	no	0	0	0	0	0	0	0	3	1	1	1	1	1	1	1	1	1	1	1	1	1	1	1	1	1
393	50	unmarried	1,000,000-2,000,000 JPY	no	0	0	0	0	0	0	0	1	1	1	1	1	1	1	1	1	1	2	1	1	1	1	1	1	1
394	64	married	1,000,000-2,000,000 JPY	yes	0	0	0	1	0	0	0	4	2	2	1	2	2	2	2	2	2	2	2	4	1	1	2	2	2
395	54	married	7,000,000-8,000,000 JPY	yes	0	1	0	0	0	0	0	2	1	1	1	1	1	1	1	1	1	1	1	1	1	1	2	2	2
396	49	unmarried	3,000,000-4,000,000 JPY	no	5	4	5	0	2	2	2	6	3	1	1	4	5	5	5	3	3	3	5	5	3	1	5	5	5
397	67	married	8,000,000-9,000,000 JPY	yes	5	5	3	0	1	0	1	3	3	2	1	2	3	2	2	1	1	2	1	2	2	1	2	3	2
398	72	married	7,000,000-8,000,000 JPY	yes	1	1	0	1	0	0	1	5	1	2	2	2	2	1	1	1	2	2	1	2	1	1	2	1	2
399	50	unmarried	18,000,000-20,000,000 JPY	no	0	0	0	0	0	0	0	0	1	1	1	1	1	1	1	1	1	1	1	1	1	1	1	1	1
400	48	unmarried	12,000,000-15,000,000 JPY	yes	4	2	0	1	2	0	5	3	3	3	2	2	2	2	2	2	2	2	3	3	2	2	2	3	2
401	80	married	3,000,000-4,000,000 JPY	yes	4	4	5	5	5	5	2	5	2	3	1	1	3	1	1	1	1	2	1	3	1	1	3	5	3
402	70	married	4,000,000-5,000,000 JPY	yes	0	1	0	0	0	0	2	3	1	1	1	1	1	1	1	1	1	1	1	1	1	1	1	1	1
403	47	unmarried	2,000,000-3,000,000 JPY	no	0	0	0	0	0	0	1	3	1	1	1	1	1	1	1	1	1	1	1	2	1	1	1	1	1
404	51	married	6,000,000-7,000,000 JPY	yes	0	0	5	0	1	5	1	1	1	2	2	2	3	2	2	1	3	3	2	3	1	1	2	4	1
405	68	married	3,000,000-4,000,000 JPY	no	0	0	0	0	0	0	1	1	1	2	1	2	1	1	1	1	1	1	1	1	2	2	2	2	2

406	70	married	6,000,000-7,000,000 JPY	yes	0	0	0	0	1	0	2	2	2	1	1	2	1	1	1	1	2	3	1	3	1	1	2	4	4
407	58	married	8,000,000-9,000,000 JPY	yes	3	3	3	3	2	1	1	4	2	2	2	3	2	2	2	1	2	1	1	2	1	1	1	1	1
408	61	unmarried	3,000,000-4,000,000 JPY	no	1	0	0	0	0	0	0	1	3	1	1	2	2	2	3	2	3	4	3	3	2	1	4	4	4
409	42	married	5,000,000-6,000,000 JPY	yes	0	0	0	0	0	0	0	1	2	2	1	1	2	2	2	2	2	2	2	2	2	2	2	2	2
410	56	unmarried	less than 1,000,000 JPY	no	1	1	1	0	1	1	0	1	2	3	2	4	3	2	2	1	2	2	2	3	2	1	3	3	3
411	51	unmarried	2,000,000-3,000,000 JPY	no	1	0	0	0	1	0	1	3	1	1	1	2	1	1	1	1	1	1	1	1	1	1	1	1	1
412	46	married	8,000,000-9,000,000 JPY	yes	0	0	0	0	0	0	0	0	1	1	1	1	1	1	1	1	1	2	1	1	1	1	2	2	1
413	47	unmarried	4,000,000-5,000,000 JPY	no	0	0	0	0	0	0	0	0	1	1	1	1	1	1	1	1	1	1	1	1	1	1	1	1	1
414	71	married	15,000,000-18,000,000 JPY	yes	0	0	0	0	5	0	1	2	2	3	1	2	2	1	1	1	3	2	1	3	2	2	3	4	3
415	74	married	1,000,000-2,000,000 JPY	yes	1	0	1	0	1	0	1	3	2	2	2	2	2	2	2	1	2	1	1	2	1	1	2	2	2
416	71	married	less than 1,000,000 JPY	yes	1	2	2	1	2	1	3	3	2	1	1	1	2	2	2	1	2	3	1	4	2	2	4	3	3
417	69	unmarried	3,000,000-4,000,000 JPY	no	1	2	0	0	2	0	2	2	2	2	1	2	2	1	1	1	2	2	2	3	1	2	3	3	3
418	53	married	5,000,000-6,000,000 JPY	yes	0	0	0	0	0	0	1	3	3	2	2	2	3	2	2	3	2	2	2	3	2	2	2	2	3
419	73	unmarried	less than 1,000,000 JPY	no	0	0	0	0	0	0	0	1	1	1	1	1	1	1	1	1	1	2	1	2	1	1	2	2	1
420	57	married	12,000,000-15,000,000 JPY	yes	0	0	0	0	0	0	0	0	1	1	1	1	1	1	1	1	1	1	1	1	1	1	1	1	1
421	72	married	3,000,000-4,000,000 JPY	yes	0	0	0	0	0	0	1	1	1	3	1	1	2	1	1	1	1	2	1	2	1	1	3	3	1
422	70	married	less than 1,000,000 JPY	no	2	1	0	0	5	1	0	5	2	3	2	1	2	1	1	1	2	2	3	4	1	1	3	3	1
423	71	married	10,000,000-12,000,000 JPY	yes	0	1	0	1	1	0	0	1	1	2	1	1	2	1	1	1	3	2	1	1	1	1	5	5	4
424	61	unmarried	4,000,000-5,000,000 JPY	yes	1	0	0	1	1	0	1	3	2	2	1	3	2	2	2	1	2	2	1	3	1	1	1	2	2
425	50	married	5,000,000-6,000,000 JPY	yes	0	0	0	0	0	0	0	0	3	3	1	3	4	3	3	3	3	3	3	4	3	3	3	3	3
426	41	unmarried	3,000,000-4,000,000 JPY	no	0	0	0	0	0	0	1	1	1	1	1	1	1	1	1	1	1	1	1	1	1	1	1	1	1
427	79	married	4,000,000-5,000,000 JPY	yes	1	1	0	0	5	0	2	3	2	2	1	1	2	1	1	1	1	3	1	3	1	1	5	5	4
428	77	married	7,000,000-8,000,000 JPY	yes	1	1	0	1	1	0	3	4	2	2	1	2	3	1	1	1	1	2	1	3	1	1	2	4	2
429	55	married	9,000,000-10,000,000 JPY	no	0	0	0	0	0	0	0	1	1	1	1	1	2	1	1	1	1	1	1	1	1	1	1	2	1
430	58	unmarried	less than 1,000,000 JPY	no	0	0	0	0	0	0	0	2	1	1	1	1	1	1	1	1	1	1	1	3	1	1	1	1	1
431	73	married	7,000,000-8,000,000 JPY	yes	0	1	0	0	0	0	2	3	1	2	1	1	1	1	1	1	1	2	1	1	1	1	2	3	3
432	60	unmarried	less than 1,000,000 JPY	no	5	5	0	5	5	0	0	5	3	1	1	5	3	1	1	1	2	3	1	1	1	1	2	2	5
433	65	married	3,000,000-4,000,000 JPY	yes	0	1	1	0	1	1	1	3	1	1	1	1	1	1	1	1	1	2	1	1	1	1	3	3	3
434	74	married	1,000,000-2,000,000 JPY	yes	1	1	0	1	1	0	2	6	3	3	1	2	3	3	2	2	3	4	3	4	1	3	4	3	2
435	67	married	12,000,000-15,000,000 JPY	yes	3	3	3	3	5	4	2	4	3	1	1	1	2	1	1	1	2	2	1	3	1	2	2	3	3
436	57	unmarried	9,000,000-10,000,000 JPY	no	1	1	1	0	1	1	1	3	3	4	2	5	1	1	2	3	2	1	3	3	2	2	4	4	5
437	50	married	5,000,000-6,000,000 JPY	no	0	2	0	0	0	0	0	1	2	1	1	2	2	1	1	1	1	2	1	3	1	1	1	1	1
438	62	unmarried	2,000,000-3,000,000 JPY	no	0	0	0	1	1	1	1	2	1	2	1	1	1	1	1	1	2	2	1	2	1	1	2	3	2
439	50	unmarried	4,000,000-5,000,000 JPY	no	0	0	0	0	1	0	0	1	1	1	1	1	1	1	1	1	1	1	1	1	1	2	1	1	1
440	40	married	5,000,000-6,000,000 JPY	no	0	0	0	1	0	0	0	2	2	1	1	1	2	1	1	1	2	1	1	2	1	1	2	1	2
441	64	married	1,000,000-2,000,000 JPY	yes	0	0	0	0	0	0	1	1	2	2	1	2	3	2	1	1	3	3	2	3	1	1	2	2	1
442	42	unmarried	3,000,000-4,000,000 JPY	no	0	0	0	0	0	0	0	0	1	1	1	1	1	2	1	1	1	1	1	1	1	1	1	1	1
443	59	married	12,000,000-15,000,000 JPY	yes	1	1	0	0	1	0	0	2	1	2	1	2	4	2	2	2	3	2	2	3	2	1	2	4	4
444	53	unmarried	2,000,000-3,000,000 JPY	no	0	0	0	0	0	0	1	1	1	1	1	2	1	1	1	1	1	2	1	2	1	1	1	1	1
445	70	married	5,000,000-6,000,000 JPY	yes	3	0	0	0	0	0	1	3	2	4	2	2	2	1	2	2	3	3	2	3	2	2	2	2	2
446	69	married	2,000,000-3,000,000 JPY	yes	1	1	0	3	5	0	0	4	1	1	1	1	1	1	1	1	1	2	1	2	1	1	3	3	3
447	76	married	2,000,000-3,000,000 JPY	yes	4	4	4	4	5	5	2	5	3	3	2	2	2	2	2	1	2	3	2	2	2	1	2	1	2
448	74	married	3,000,000-4,000,000 JPY	yes	0	5	2	1	5	5	3	4	1	2	1	1	2	1	1	1	3	3	1	5	3	5	5	5	5
449	60	married	4,000,000-5,000,000 JPY	no	5	1	0	0	1	0	1	4	3	2	1	2	3	2	2	2	2	3	2	2	3	3	2	3	2
450	54	married	12,000,000-15,000,000 JPY	yes	1	2	4	0	2	3	0	4	2	2	1	3	3	2	2	1	3	3	1	2	1	1	3	2	2
451	72	married	2,000,000-3,000,000 JPY	no	1	1	0	0	5	0	5	5	3	2	1	2	2	3	3	3	3	3	3	3	3	1	5	5	1
452	66	married	3,000,000-4,000,000 JPY	yes	0	1	0	0	0	0	2	1	1	1	1	1	1	1	1	1	1	2	1	2	1	1	2	2	2
453	49	unmarried	3,000,000-4,000,000 JPY	no	0	0	0	0	0	0	0	2	2	2	1	2	2	2	1	1	2	1	1	1	1	1	1	1	1
454	59	married	7,000,000-8,000,000 JPY	no	0	0	0	0	0	0	0	1	1	1	1	1	1	1	1	1	1	1	1	2	1	1	2	2	2
455	73	married	9,000,000-10,000,000 JPY	yes	4	1	3	2	2	0	1	5	1	1	1	1	2	1	1	1	1	1	1	2	1	1	1	1	1
456	50	married	7,000,000-8,000,000 JPY	yes	3	5	0	0	0	0	2	4	1	1	1	3	2	1	1	1	3	3	1	5	2	1	4	4	3
457	73	married	2,000,000-3,000,000 JPY	yes	2	2	2	3	3	3	2	3	3	3	3	3	3	3	3	3	3	3	3	3	3	3	3	3	3
458	48	married	4,000,000-5,000,000 JPY	no	3	1	3	1	3	0	1	2	4	3	1	2	4	2	2	1	4	2	5	5	2	1	4	4	4
459	60	married	8,000,000-9,000,000 JPY	yes	0	0	0	0	0	0	0	1	1	2	1	1	1	1	1	1	1	1	1	1	1	1	1	1	1
460	72	married	12,000,000-15,000,000 JPY	yes	0	0	0	0	1	0	0	3	2	2	2	1	2	1	1	1	1	2	1	2	1	1	3	4	1
461	44	married	6,000,000-7,000,000 JPY	no	1	1	0	0	0	0	0	1	2	2	2	2	2	3	3	1	2	2	1	1	1	2	2	2	2
462	72	married	4,000,000-5,000,000 JPY	yes	0	3	5	1	5	5	3	6	3	3	1	3	2	1	1	1	2	2	1	3	1	1	4	4	4
463	41	married	4,000,000-5,000,000 JPY	yes	0	0	0	0	0	0	5	6	3	2	1	1	1	4	4	2	2	1	2	1	1	1	1	1	2

464	74	married	20,000,000 or more JPY	yes	2	1	1	1	2	1	1	3	3	4	3	4	2	2	3	1	2	3	2	3	2	2	3	3	3
465	60	married	9,000,000-10,000,000 JPY	yes	0	0	0	0	0	0	5	1	1	1	1	1	1	1	1	1	1	1	1	1	1	1	1	1	1
466	60	married	8,000,000-9,000,000 JPY	yes	0	0	0	0	0	0	0	3	1	1	1	1	2	1	1	1	1	2	1	3	1	1	1	1	2
467	42	married	6,000,000-7,000,000 JPY	yes	0	5	0	0	4	0	1	1	1	1	1	1	3	3	4	5	1	2	4	1	2	1	1	4	3
468	43	unmarried	4,000,000-5,000,000 JPY	no	0	0	0	0	0	0	0	1	4	1	1	4	2	3	4	4	4	2	5	4	4	2	2	2	2
469	51	married	less than 1,000,000 JPY	yes	0	0	0	0	0	0	5	2	1	1	1	1	1	1	1	1	1	2	1	1	1	1	1	1	1
470	61	married	2,000,000-3,000,000 JPY	yes	1	0	0	2	0	0	0	3	1	3	1	1	3	1	1	1	1	2	1	3	1	1	2	3	2
471	73	married	3,000,000-4,000,000 JPY	yes	2	2	2	2	2	2	3	4	3	1	1	2	2	1	1	1	1	1	1	2	1	2	2	2	2
472	68	unmarried	1,000,000-2,000,000 JPY	no	3	4	3	3	5	3	0	5	2	2	1	2	2	2	2	1	2	1	1	4	1	1	4	4	3
473	59	married	12,000,000-15,000,000 JPY	yes	3	3	2	2	2	1	2	3	2	3	1	2	3	3	3	3	3	3	4	4	2	3	3	4	3
474	71	married	1,000,000-2,000,000 JPY	yes	1	1	1	0	1	0	0	2	2	2	2	1	2	2	2	1	1	2	1	2	1	1	2	2	2
475	72	married	5,000,000-6,000,000 JPY	yes	0	3	5	0	5	0	2	3	3	3	1	1	2	3	2	2	3	3	4	4	4	3	5	5	4
476	45	married	6,000,000-7,000,000 JPY	yes	0	1	1	2	1	0	1	5	3	2	1	3	3	3	3	3	3	3	3	5	2	1	2	2	3
477	72	married	3,000,000-4,000,000 JPY	no	3	4	3	4	5	4	1	6	3	3	3	3	3	3	3	3	4	3	3	3	3	3	3	2	2
478	73	married	7,000,000-8,000,000 JPY	yes	0	0	0	0	1	0	0	2	1	2	1	1	2	1	1	1	2	2	1	2	1	1	3	3	2
479	55	married	10,000,000-12,000,000 JPY	yes	3	1	1	1	1	0	2	5	3	3	2	2	3	2	3	3	3	3	3	3	3	2	3	3	3
480	60	unmarried	5,000,000-6,000,000 JPY	yes	2	4	0	1	0	0	2	3	3	3	3	3	3	3	3	3	3	3	3	3	3	3	3	3	3
481	67	married	12,000,000-15,000,000 JPY	yes	0	0	0	0	0	0	1	5	1	1	1	1	1	1	1	1	1	1	1	1	1	1	1	1	1
482	68	married	1,000,000-2,000,000 JPY	yes	1	1	1	1	1	0	0	3	2	2	1	2	1	2	1	1	1	2	2	2	1	1	1	1	1
483	71	married	2,000,000-3,000,000 JPY	yes	1	1	1	0	1	1	0	3	2	2	2	1	2	1	1	1	2	2	1	2	1	1	2	2	2
484	62	unmarried	3,000,000-4,000,000 JPY	no	0	0	0	0	0	0	0	1	2	2	1	2	2	1	1	1	2	1	1	2	1	1	2	2	1
485	41	unmarried	5,000,000-6,000,000 JPY	yes	1	4	4	3	1	0	1	4	2	3	2	2	4	4	4	3	3	3	4	4	4	2	1	1	1
486	48	married	8,000,000-9,000,000 JPY	yes	1	5	5	0	0	0	1	3	2	1	2	1	1	1	1	1	1	1	1	3	1	3	3	4	4
487	79	married	3,000,000-4,000,000 JPY	yes	0	1	0	0	0	0	1	2	1	1	1	2	1	1	1	1	1	2	1	3	1	1	2	3	3
488	66	unmarried	less than 1,000,000 JPY	yes	5	5	5	5	0	5	4	4	3	3	1	3	2	1	1	2	2	4	2	2	1	3	3	4	1
489	51	unmarried	less than 1,000,000 JPY	no	0	0	0	0	0	0	0	0	1	1	1	1	1	1	1	2	1	1	1	1	1	1	1	1	1
490	56	married	3,000,000-4,000,000 JPY	yes	0	0	0	5	0	0	1	1	1	3	2	2	2	1	1	1	2	2	1	2	1	1	1	2	2
491	78	married	8,000,000-9,000,000 JPY	yes	3	3	2	2	5	3	3	3	2	1	1	2	2	2	1	1	2	2	1	3	1	1	2	3	2
492	62	married	6,000,000-7,000,000 JPY	yes	2	3	3	3	3	2	3	2	3	4	3	3	3	3	4	4	4	4	3	3	3	3	4	4	4
493	58	married	9,000,000-10,000,000 JPY	no	5	1	2	1	1	1	1	3	3	3	3	2	3	1	3	3	3	3	3	3	2	3	3	2	3
494	58	unmarried	less than 1,000,000 JPY	no	1	2	2	1	2	2	2	5	3	2	3	4	4	4	3	4	3	4	4	3	3	3	3	4	4
495	71	married	4,000,000-5,000,000 JPY	yes	5	1	5	0	5	0	2	6	3	2	1	2	2	1	2	1	2	3	2	3	1	1	3	4	2
496	77	married	2,000,000-3,000,000 JPY	yes	1	1	0	0	0	0	1	3	1	1	1	1	1	2	2	1	1	2	2	2	1	2	1	1	1
497	51	unmarried	8,000,000-9,000,000 JPY	no	4	5	1	2	4	3	0	4	3	1	1	2	2	1	1	1	3	1	2	4	1	1	3	3	3
498	52	unmarried	less than 1,000,000 JPY	no	0	0	0	0	0	0	1	3	1	1	1	1	1	1	1	1	1	1	1	1	1	1	1	1	1
499	40	married	5,000,000-6,000,000 JPY	yes	1	1	0	0	1	0	0	3	3	1	2	1	3	2	2	1	3	3	2	4	1	1	2	1	1
500	66	married	2,000,000-3,000,000 JPY	yes	3	3	2	4	3	0	5	5	3	2	1	2	2	2	2	2	2	2	2	2	3	3	3	3	3
501	66	married	2,000,000-3,000,000 JPY	yes	2	1	0	0	1	1	0	2	1	1	1	2	2	1	1	1	1	2	1	2	1	1	2	2	1
502	54	married	8,000,000-9,000,000 JPY	yes	0	3	0	1	2	0	2	4	2	3	2	3	2	3	3	2	2	3	3	3	3	2	4	4	4
503	53	married	4,000,000-5,000,000 JPY	yes	0	1	0	1	1	0	0	2	2	3	2	3	2	1	1	1	1	3	1	2	1	1	2	3	2
504	72	married	5,000,000-6,000,000 JPY	yes	1	4	4	4	3	3	2	5	2	3	1	2	2	1	1	1	2	2	1	2	2	2	5	5	5
505	69	married	3,000,000-4,000,000 JPY	yes	1	2	5	1	5	0	5	4	1	2	1	2	1	2	1	1	2	2	1	3	1	1	3	2	4
506	69	married	less than 1,000,000 JPY	yes	3	1	1	1	4	1	2	3	1	2	1	2	1	2	1	1	1	2	1	2	1	1	1	2	1
507	46	married	7,000,000-8,000,000 JPY	yes	1	0	0	0	1	0	0	0	2	1	1	3	2	3	1	2	1	1	2	3	2	2	5	4	4
508	45	married	9,000,000-10,000,000 JPY	yes	5	5	5	0	0	0	0	0	4	1	1	1	1	2	1	1	1	4	4	1	1	4	1	1	5
509	61	married	18,000,000-20,000,000 JPY	yes	0	0	0	0	0	0	1	1	4	1	1	1	1	2	1	1	1	2	1	2	1	1	2	2	2
510	66	married	3,000,000-4,000,000 JPY	yes	4	3	0	0	0	0	1	3	1	3	2	2	2	1	1	1	2	4	1	3	1	1	3	5	2
511	48	married	12,000,000-15,000,000 JPY	yes	0	1	0	1	1	0	1	3	1	1	1	2	1	1	1	1	2	1	2	1	1	1	2	2	2
512	46	married	5,000,000-6,000,000 JPY	no	0	1	0	1	0	0	1	4	3	1	2	2	2	1	1	1	1	2	1	2	1	1	1	1	1
513	54	married	6,000,000-7,000,000 JPY	no	2	2	0	0	0	2	0	3	2	1	2	2	3	2	1	1	2	3	1	2	1	1	2	2	1
514	42	married	3,000,000-4,000,000 JPY	yes	5	5	5	5	5	5	2	3	3	3	3	3	3	3	3	3	3	3	3	3	3	3	3	3	3
515	57	unmarried	1,000,000-2,000,000 JPY	no	0	0	0	0	0	0	0	3	1	1	1	1	1	1	1	1	1	1	1	1	1	1	1	1	1
516	64	unmarried	less than 1,000,000 JPY	no	0	0	0	0	0	0	1	1	1	1	1	1	1	1	1	1	1	1	1	1	1	1	1	1	1
517	40	married	3,000,000-4,000,000 JPY	no	1	1	0	0	0	0	1	4	3	3	2	3	3	2	1	1	1	2	1	3	1	1	1	1	1
518	71	unmarried	3,000,000-4,000,000 JPY	no	1	1	0	2	5	1	1	4	2	1	1	2	2	1	1	1	1	2	1	3	1	1	2	2	2
519	53	married	3,000,000-4,000,000 JPY	yes	0	0	0	0	0	0	0	0	1	1	1	1	1	1	1	1	1	1	1	1	1	1	1	1	1
520	40	unmarried	3,000,000-4,000,000 JPY	no	2	2	1	0	1	0	2	4	2	2	2	4	3	2	2	1	2	2	2	5	3	1	2	2	2
521	59	married	12,000,000-15,000,000 JPY	yes	2	2	2	2	2	1	2	4	2	2	1	1	3	1	2	2	2	2	2	3	3	1	4	4	3

522	76	unmarried	4,000,000-5,000,000 JPY	yes	1	1	1	1	1	1	1	1	2	2	2	2	2	2	2	2	2	2	2	2	2	2	2	3	2
523	60	married	12,000,000-15,000,000 JPY	yes	5	4	4	3	5	5	5	5	4	3	2	4	3	3	4	1	4	3	1	3	3	2	3	3	3
524	58	married	7,000,000-8,000,000 JPY	yes	0	1	1	1	1	0	2	4	3	3	1	1	4	3	3	1	2	4	2	4	1	1	1	2	2
525	54	unmarried	2,000,000-3,000,000 JPY	no	1	1	1	1	1	0	1	5	2	2	2	2	2	2	2	2	2	3	2	2	2	1	2	2	2
526	48	married	2,000,000-3,000,000 JPY	no	0	0	0	0	0	0	1	2	2	1	2	1	2	1	1	1	2	2	1	1	2	1	1	2	1
527	74	married	less than 1,000,000 JPY	yes	0	0	0	1	0	0	1	2	1	1	1	1	1	1	1	1	1	1	1	3	1	1	2	2	2
528	64	married	4,000,000-5,000,000 JPY	yes	1	1	0	1	2	0	2	4	3	1	1	1	3	1	1	1	1	2	1	2	1	1	2	2	2
529	51	unmarried	3,000,000-4,000,000 JPY	no	5	0	5	3	5	5	0	5	4	3	4	3	3	5	5	3	4	3	5	3	3	2	3	3	2
530	71	married	9,000,000-10,000,000 JPY	yes	1	1	0	0	0	0	3	3	2	2	2	2	2	1	1	2	2	2	2	1	2	2	2	2	2
531	69	married	12,000,000-15,000,000 JPY	yes	5	2	5	1	5	1	2	5	3	1	2	2	3	2	1	1	3	2	1	3	2	1	3	4	4
532	70	married	1,000,000-2,000,000 JPY	yes	1	1	2	1	2	3	3	5	3	3	2	3	3	2	2	2	5	4	3	3	3	3	3	4	3
533	64	married	5,000,000-6,000,000 JPY	yes	0	0	0	0	0	0	0	2	1	1	1	1	2	2	1	1	2	2	1	3	1	2	3	3	3
534	53	unmarried	8,000,000-9,000,000 JPY	no	0	2	0	0	0	0	1	3	1	1	1	2	1	2	3	2	1	1	2	1	1	1	2	1	2
535	48	unmarried	7,000,000-8,000,000 JPY	no	2	1	1	0	0	0	0	3	2	2	2	2	1	2	2	1	1	2	1	2	1	1	1	2	2
536	58	unmarried	6,000,000-7,000,000 JPY	no	1	1	0	1	1	0	0	3	2	2	1	2	2	2	2	1	3	3	2	3	2	1	1	1	1
537	51	unmarried	less than 1,000,000 JPY	no	0	0	0	0	0	0	0	3	1	1	1	1	1	1	1	1	1	1	1	1	1	1	1	1	1
538	54	unmarried	8,000,000-9,000,000 JPY	no	2	0	2	0	1	0	1	3	1	1	1	1	4	2	1	1	1	1	1	4	1	1	3	3	3
539	47	unmarried	1,000,000-2,000,000 JPY	no	0	0	0	0	0	0	1	3	1	1	1	1	1	1	1	1	1	1	1	2	1	1	1	1	1
540	44	married	2,000,000-3,000,000 JPY	yes	0	0	0	0	0	0	1	0	1	1	1	1	1	2	1	1	1	1	1	1	1	1	1	1	1
541	61	married	5,000,000-6,000,000 JPY	yes	4	4	3	1	3	1	4	5	3	3	2	3	4	3	4	3	3	3	3	4	3	2	2	3	3
542	60	married	8,000,000-9,000,000 JPY	no	1	1	1	0	2	0	1	4	2	2	2	3	2	1	1	1	2	3	2	3	1	1	2	2	2
543	74	married	4,000,000-5,000,000 JPY	yes	0	0	0	0	0	0	1	0	1	1	1	1	1	1	1	1	1	3	1	3	1	1	3	4	2
544	79	married	10,000,000-12,000,000 JPY	yes	0	0	0	0	0	0	0	0	1	1	1	1	1	1	1	1	1	1	1	1	1	1	1	1	1
545	73	married	12,000,000-15,000,000 JPY	yes	0	1	1	0	0	0	1	4	3	2	2	2	2	1	1	1	1	2	1	2	1	2	2	2	2
546	57	married	less than 1,000,000 JPY	no	0	0	0	0	0	0	2	3	1	1	1	1	1	1	1	1	1	1	1	1	1	1	1	1	1
547	52	married	8,000,000-9,000,000 JPY	yes	1	2	1	1	2	0	0	4	2	3	1	2	3	2	1	1	2	2	2	3	1	1	3	3	3
548	71	married	5,000,000-6,000,000 JPY	yes	5	3	5	0	5	5	1	6	4	4	2	3	3	1	3	2	3	4	2	4	2	1	5	5	5
549	59	married	10,000,000-12,000,000 JPY	yes	0	1	0	1	0	0	1	1	1	1	1	1	2	2	1	1	2	2	2	2	1	2	3	3	3
550	71	unmarried	2,000,000-3,000,000 JPY	no	0	0	0	0	1	1	1	3	1	2	1	2	2	1	1	2	2	3	2	2	1	1	2	3	3
551	47	unmarried	1,000,000-2,000,000 JPY	no	2	3	2	4	1	2	2	4	3	3	3	4	4	5	5	5	4	4	4	5	4	1	3	5	4
552	64	married	6,000,000-7,000,000 JPY	yes	2	2	3	3	3	2	2	2	3	2	3	2	3	3	2	2	2	3	2	2	2	3	3	2	2
553	70	married	2,000,000-3,000,000 JPY	yes	1	2	2	1	5	1	1	4	2	2	1	2	2	1	1	1	2	2	1	3	2	2	3	3	3
554	49	unmarried	9,000,000-10,000,000 JPY	no	0	0	0	0	0	0	0	2	1	1	1	1	2	1	1	1	1	2	1	2	1	1	2	1	1
555	40	unmarried	2,000,000-3,000,000 JPY	no	1	2	2	1	0	0	1	3	3	3	3	3	2	3	4	3	3	4	2	3	3	4	3	3	3
556	49	unmarried	4,000,000-5,000,000 JPY	no	5	3	1	1	4	1	1	4	2	1	1	2	2	1	1	1	3	2	2	4	2	1	3	1	1
557	49	married	7,000,000-8,000,000 JPY	yes	0	0	0	0	0	0	0	0	1	5	5	5	5	5	5	5	5	5	5	5	2	1	1	1	1
558	49	married	5,000,000-6,000,000 JPY	yes	0	1	0	0	0	0	0	1	1	1	1	3	2	1	1	1	2	1	1	3	1	1	1	1	1
559	69	married	3,000,000-4,000,000 JPY	yes	0	0	1	0	1	0	5	3	2	3	1	2	2	2	1	1	2	3	1	2	1	1	2	2	2
560	70	married	2,000,000-3,000,000 JPY	yes	1	1	3	4	5	4	2	3	2	1	3	2	3	1	2	1	1	2	2	3	2	2	2	3	2
561	72	married	1,000,000-2,000,000 JPY	no	0	0	0	0	0	0	1	1	2	5	1	2	2	2	1	1	1	1	1	1	1	2	3	3	3
562	74	married	9,000,000-10,000,000 JPY	yes	3	1	2	0	2	2	0	3	1	2	2	1	2	2	1	1	2	2	1	3	1	2	3	4	3
563	72	married	3,000,000-4,000,000 JPY	yes	1	1	1	2	4	1	1	3	3	3	1	2	2	2	1	1	2	3	2	4	3	2	4	4	4
564	79	married	2,000,000-3,000,000 JPY	yes	0	1	0	0	1	0	5	2	1	2	2	3	2	1	1	1	1	2	1	3	1	1	5	5	5
565	42	unmarried	less than 1,000,000 JPY	no	1	3	0	0	5	5	0	3	3	2	1	4	4	1	1	1	4	1	3	5	4	1	3	5	2
566	47	married	5,000,000-6,000,000 JPY	no	0	3	0	0	0	0	1	2	2	1	2	1	1	3	1	4	2	2	5	1	1	1	3	2	1
567	43	married	6,000,000-7,000,000 JPY	yes	2	1	3	3	3	3	1	0	3	4	2	4	1	4	3	3	4	4	1	3	2	3	5	2	1
568	61	married	2,000,000-3,000,000 JPY	yes	1	1	1	1	1	1	0	3	1	2	1	2	2	1	1	1	2	2	1	2	1	1	2	2	2
569	53	married	18,000,000-20,000,000 JPY	yes	1	1	0	1	1	0	1	3	2	1	1	2	2	1	1	1	1	1	1	2	1	2	2	2	1
570	81	married	7,000,000-8,000,000 JPY	yes	1	0	3	0	5	2	2	3	2	3	2	2	2	2	2	1	3	3	2	4	1	1	5	5	5
571	61	married	4,000,000-5,000,000 JPY	no	0	0	0	0	0	0	0	1	1	3	1	1	2	1	1	1	1	2	1	1	1	1	1	1	1
572	57	married	3,000,000-4,000,000 JPY	yes	3	0	0	1	4	4	0	2	3	3	2	3	2	2	2	2	4	2	4	3	3	3	3	2	3
573	56	married	7,000,000-8,000,000 JPY	yes	0	1	4	5	5	0	0	3	2	3	2	1	2	1	1	1	3	3	2	3	1	1	3	3	2
574	50	married	9,000,000-10,000,000 JPY	yes	0	0	0	0	0	1	2	0	1	1	1	1	1	1	2	2	1	2	1	2	1	1	5	4	5
575	47	unmarried	6,000,000-7,000,000 JPY	no	0	1	1	0	0	0	0	1	1	1	1	1	1	1	1	1	1	2	1	2	1	1	1	1	1
576	44	married	12,000,000-15,000,000 JPY	yes	0	0	0	0	0	0	0	2	1	1	1	1	1	1	1	1	1	2	1	3	2	2	3	3	3
577	58	married	7,000,000-8,000,000 JPY	yes	1	1	1	1	3	0	5	4	2	3	2	4	2	2	2	1	2	2	2	3	1	3	3	2	2
578	41	unmarried	5,000,000-6,000,000 JPY	no	0	2	0	1	4	1	0	5	2	1	1	3	2	1	1	1	1	1	1	4	1	3	4	4	1
579	63	married	2,000,000-3,000,000 JPY	yes	0	0	0	0	0	0	1	3	1	1	1	1	1	1	1	1	1	1	1	1	1	1	1	1	1

580	50	married	7,000,000-8,000,000 JPY	no	0	1	0	0	0	0	1	3	1	1	1	1	1	1	1	1	1	1	1	1	1	1	1	1	1
581	81	married	2,000,000-3,000,000 JPY	yes	0	1	1	0	1	1	1	2	2	2	1	2	2	1	1	1	1	2	1	3	1	1	3	3	3
582	59	unmarried	less than 1,000,000 JPY	yes	5	5	5	0	5	0	1	3	1	1	1	4	3	2	2	2	2	3	3	4	1	1	4	4	1
583	51	married	7,000,000-8,000,000 JPY	yes	1	1	0	0	1	0	1	3	2	3	2	3	2	2	2	2	3	3	2	2	2	1	2	3	2
584	60	married	15,000,000-18,000,000 JPY	yes	4	1	3	2	5	0	0	2	2	2	2	3	3	2	3	3	3	3	3	4	4	1	3	3	1
585	55	unmarried	2,000,000-3,000,000 JPY	no	0	1	0	3	0	0	0	3	2	3	1	3	3	3	3	3	3	2	3	3	3	2	2	2	1
586	73	married	7,000,000-8,000,000 JPY	yes	0	1	0	0	0	0	0	2	1	2	1	3	2	2	1	1	1	2	1	3	1	1	2	2	1
587	62	unmarried	3,000,000-4,000,000 JPY	no	1	3	3	4	4	3	2	4	3	2	2	3	3	2	3	2	3	3	2	4	3	4	4	4	4
588	71	married	3,000,000-4,000,000 JPY	yes	1	1	0	2	5	3	3	5	2	1	2	3	2	2	3	2	2	2	3	3	1	1	4	5	4
589	49	unmarried	3,000,000-4,000,000 JPY	yes	1	1	0	0	0	1	1	3	2	3	1	3	2	2	1	1	1	1	1	1	1	1	1	1	1
590	46	married	4,000,000-5,000,000 JPY	yes	5	4	0	3	1	4	3	4	3	4	4	3	2	2	2	3	2	3	3	4	3	3	2	3	2
591	57	married	4,000,000-5,000,000 JPY	yes	2	4	1	1	2	2	2	5	2	2	1	3	2	2	1	1	3	2	1	3	1	1	3	3	3
592	66	married	2,000,000-3,000,000 JPY	yes	1	4	0	4	5	0	5	4	2	3	2	2	2	2	1	1	1	2	1	3	2	2	2	4	2
593	72	married	2,000,000-3,000,000 JPY	no	0	2	0	0	5	0	2	5	4	5	2	4	4	3	4	4	4	5	3	5	4	3	5	5	5
594	72	married	3,000,000-4,000,000 JPY	yes	1	1	0	0	5	4	2	4	1	2	1	1	2	1	1	1	1	1	1	3	1	1	2	2	2
595	62	married	12,000,000-15,000,000 JPY	yes	0	1	0	2	1	0	1	4	1	1	1	1	2	1	2	1	2	1	2	3	1	1	2	3	2
596	76	married	4,000,000-5,000,000 JPY	no	0	0	0	0	0	0	1	3	1	1	1	1	1	1	1	1	1	1	1	1	1	1	1	1	1
597	60	married	5,000,000-6,000,000 JPY	yes	0	0	0	0	4	0	0	1	1	3	1	1	1	2	1	1	1	1	1	1	1	1	2	3	2
598	46	married	18,000,000-20,000,000 JPY	yes	0	0	0	0	0	0	0	1	3	3	3	3	3	1	1	1	3	3	1	1	1	1	1	1	1
599	67	married	9,000,000-10,000,000 JPY	yes	4	3	3	5	5	5	1	3	3	2	3	4	4	1	1	1	4	4	2	5	2	2	5	5	5
600	76	unmarried	4,000,000-5,000,000 JPY	no	0	2	0	0	0	0	1	1	1	3	2	1	2	1	2	1	2	2	1	2	1	1	1	1	1
601	106	unmarried	less than 1,000,000 JPY	yes	0	0	0	0	0	0	0	0	1	1	1	1	1	1	1	1	1	1	1	1	1	1	1	1	1
602	68	married	2,000,000-3,000,000 JPY	yes	2	2	2	2	2	2	1	2	3	3	3	3	3	3	3	3	3	3	3	3	3	3	3	3	3
603	72	married	1,000,000-2,000,000 JPY	yes	1	1	0	0	0	0	3	2	2	2	2	2	2	2	2	2	2	2	2	2	2	2	2	2	2
604	63	married	5,000,000-6,000,000 JPY	yes	0	0	0	0	0	0	0	1	1	1	1	1	1	1	1	1	1	2	1	1	1	1	2	2	1
605	45	married	7,000,000-8,000,000 JPY	yes	0	5	0	0	0	0	5	2	2	2	2	3	2	2	2	2	2	2	2	2	2	1	2	2	2
606	71	unmarried	4,000,000-5,000,000 JPY	yes	0	0	0	0	0	0	1	3	1	1	1	1	1	1	1	1	1	1	1	1	1	1	1	1	1
607	71	married	2,000,000-3,000,000 JPY	no	0	3	0	5	0	0	2	3	2	2	1	1	1	1	1	1	1	1	1	2	1	1	5	5	5
608	71	married	9,000,000-10,000,000 JPY	yes	0	1	0	0	0	0	0	3	3	3	1	1	1	2	1	1	2	3	1	1	1	2	2	2	2
609	55	unmarried	5,000,000-6,000,000 JPY	no	0	1	0	0	0	0	0	2	3	2	4	3	2	3	3	3	3	3	3	3	3	1	3	4	3
610	74	married	3,000,000-4,000,000 JPY	yes	5	5	5	5	5	5	5	6	2	3	1	3	2	2	1	1	2	3	1	5	4	2	5	5	5
611	62	married	less than 1,000,000 JPY	no	0	0	0	0	0	0	0	1	1	1	1	1	1	1	1	1	1	1	1	1	1	1	1	1	1
612	58	unmarried	1,000,000-2,000,000 JPY	no	0	0	0	0	0	0	0	1	1	1	1	1	1	1	1	1	1	1	1	1	1	1	1	1	1
613	60	married	4,000,000-5,000,000 JPY	yes	0	0	0	0	0	0	0	2	1	1	1	1	1	1	1	1	1	1	1	1	1	1	1	1	1
614	42	unmarried	less than 1,000,000 JPY	no	1	1	0	2	1	0	3	3	4	3	1	4	2	2	1	2	4	3	1	3	4	1	3	3	3
615	73	unmarried	2,000,000-3,000,000 JPY	no	1	1	0	1	1	0	3	4	3	2	1	1	1	1	1	2	2	2	2	3	3	1	5	5	4
616	75	married	1,000,000-2,000,000 JPY	no	4	3	0	4	3	1	3	6	1	2	1	1	3	2	2	1	1	1	2	2	2	2	1	2	1
617	44	unmarried	5,000,000-6,000,000 JPY	no	2	0	0	0	0	1	1	3	2	3	2	3	4	4	5	3	4	2	5	3	5	2	3	2	3
618	41	married	9,000,000-10,000,000 JPY	yes	0	0	0	0	0	0	1	1	1	2	1	1	2	1	1	1	1	1	1	2	1	1	2	2	1
619	57	unmarried	1,000,000-2,000,000 JPY	no	1	1	1	1	1	1	4	3	2	2	1	2	3	1	1	1	3	3	2	4	2	1	4	5	3
620	41	unmarried	1,000,000-2,000,000 JPY	no	0	0	0	0	0	0	0	0	1	3	1	1	2	1	1	1	1	1	1	2	1	1	1	2	1
621	54	married	5,000,000-6,000,000 JPY	yes	5	5	0	0	0	0	0	3	3	3	1	3	5	2	3	2	1	1	2	5	1	1	1	1	1
622	62	married	2,000,000-3,000,000 JPY	yes	0	0	0	0	0	0	0	1	2	2	1	1	1	1	1	1	1	1	1	2	1	1	1	2	2
623	43	married	7,000,000-8,000,000 JPY	yes	3	1	0	3	0	0	2	4	2	3	4	4	5	3	2	1	3	3	1	1	1	1	1	1	1
624	54	married	10,000,000-12,000,000 JPY	yes	1	1	2	0	1	0	0	3	1	1	1	2	2	1	2	1	1	2	2	2	1	1	2	2	1
625	58	married	6,000,000-7,000,000 JPY	yes	0	0	0	0	0	0	1	3	3	1	4	3	3	3	3	2	3	1	2	4	4	1	2	3	2
626	43	married	3,000,000-4,000,000 JPY	no	3	5	3	1	2	3	2	4	2	2	3	4	3	3	3	3	3	3	4	4	4	2	3	4	3
627	70	married	4,000,000-5,000,000 JPY	yes	1	0	0	0	0	0	2	4	1	1	1	1	1	1	1	1	1	2	1	2	1	1	2	2	2
628	45	married	6,000,000-7,000,000 JPY	yes	1	1	1	1	1	1	0	3	2	1	1	2	2	1	1	1	1	2	2	3	1	1	2	2	2
629	61	married	3,000,000-4,000,000 JPY	yes	0	0	0	0	0	0	0	0	1	1	1	1	1	1	1	1	1	1	1	1	1	1	1	1	1
630	52	married	4,000,000-5,000,000 JPY	yes	4	0	0	0	0	0	0	2	1	1	1	1	1	1	1	1	1	1	1	1	1	1	1	1	1
631	81	married	1,000,000-2,000,000 JPY	yes	5	5	5	5	5	0	4	6	4	2	1	4	3	1	1	1	2	3	1	5	4	2	3	5	3
632	82	married	5,000,000-6,000,000 JPY	yes	0	1	1	0	1	1	0	3	3	3	1	1	1	1	1	1	1	3	1	3	1	1	4	4	4
633	41	married	12,000,000-15,000,000 JPY	yes	1	1	0	0	0	0	0	2	2	2	1	3	3	2	1	1	2	2	1	2	1	1	1	1	1
634	80	married	3,000,000-4,000,000 JPY	no	3	1	3	3	4	4	1	4	2	1	1	2	1	2	2	1	2	2	1	2	1	2	2	1	2
635	63	unmarried	4,000,000-5,000,000 JPY	no	0	1	0	0	1	0	0	0	1	2	2	3	2	2	2	1	2	2	2	2	1	1	1	1	1
636	41	unmarried	2,000,000-3,000,000 JPY	no	0	3	0	1	0	0	1	3	1	1	1	2	1	1	1	1	1	2	1	1	1	1	2	1	1
637	42	married	4,000,000-5,000,000 JPY	yes	1	1	0	0	0	0	2	4	1	1	1	1	1	2	1	1	1	1	1	1	1	1	1	1	1

638	51	unmarried	20,000,000 or more JPY	no	0	0	0	0	0	0	0	0	1	1	1	1	1	1	1	1	1	1	1	1	1	1	1	1	1
639	85	married	1,000,000-2,000,000 JPY	yes	0	0	0	0	1	0	1	1	1	2	2	1	2	1	1	1	2	2	1	2	1	1	2	2	2
640	61	unmarried	7,000,000-8,000,000 JPY	no	0	0	0	0	0	0	0	6	5	5	5	5	5	5	5	5	5	5	5	5	5	5	5	5	5
641	61	unmarried	less than 1,000,000 JPY	no	1	1	1	0	1	0	1	2	1	1	1	2	1	1	1	1	1	1	1	2	1	2	4	4	5
642	46	unmarried	4,000,000-5,000,000 JPY	no	5	3	0	0	3	0	1	4	1	1	1	2	2	2	2	1	2	1	1	2	1	1	2	1	1
643	63	unmarried	20,000,000 or more JPY	yes	0	0	0	1	2	0	0	3	1	2	1	1	1	1	1	1	1	2	2	2	1	1	1	1	1
644	43	unmarried	4,000,000-5,000,000 JPY	no	0	0	0	0	0	0	0	2	3	3	4	2	3	3	3	3	2	2	3	4	4	3	3	3	2
645	47	married	4,000,000-5,000,000 JPY	no	2	1	0	0	2	1	1	4	2	2	1	3	2	1	1	1	2	2	2	2	1	1	1	1	1
646	64	married	2,000,000-3,000,000 JPY	no	0	0	0	0	0	0	5	3	1	1	1	1	1	1	1	2	1	1	1	1	1	1	1	1	1
647	70	unmarried	5,000,000-6,000,000 JPY	yes	0	0	0	0	0	0	0	1	1	1	1	1	1	1	1	1	1	1	1	1	1	1	1	1	1
648	60	married	5,000,000-6,000,000 JPY	yes	1	5	0	3	3	0	2	6	3	3	4	4	1	4	4	1	3	3	3	4	3	5	4	5	2
649	62	married	7,000,000-8,000,000 JPY	yes	0	0	0	0	0	0	0	1	1	2	1	1	1	1	1	1	1	2	1	1	1	1	2	3	2
650	68	married	5,000,000-6,000,000 JPY	yes	5	5	0	2	1	0	2	3	2	2	1	2	2	2	2	1	2	2	1	3	1	1	3	3	1
651	66	unmarried	7,000,000-8,000,000 JPY	yes	0	1	0	0	0	0	0	1	1	1	1	1	1	1	1	1	1	1	1	2	1	2	2	3	3
652	66	married	3,000,000-4,000,000 JPY	yes	5	5	0	5	5	0	3	5	2	1	1	4	4	1	1	1	2	2	1	3	1	1	4	5	1
653	67	married	4,000,000-5,000,000 JPY	yes	0	0	0	0	0	0	0	1	2	1	1	1	1	2	2	1	1	2	1	2	1	1	2	2	2
654	52	married	9,000,000-10,000,000 JPY	yes	0	1	0	0	1	0	1	2	2	1	1	2	2	1	1	1	2	3	1	4	2	1	2	4	2
655	57	married	10,000,000-12,000,000 JPY	yes	3	2	2	2	0	1	1	4	3	3	3	2	2	1	1	1	1	1	1	1	1	1	1	1	1
656	47	unmarried	6,000,000-7,000,000 JPY	no	0	2	0	1	0	0	5	2	3	2	4	2	3	3	3	3	3	3	3	4	2	1	3	3	3
657	79	married	3,000,000-4,000,000 JPY	yes	1	1	2	3	5	0	2	4	3	2	1	3	3	3	1	1	3	3	2	3	3	3	4	4	3
658	58	married	3,000,000-4,000,000 JPY	yes	0	1	1	1	4	1	2	3	1	1	1	2	4	2	1	1	2	3	1	3	1	1	3	4	2
659	54	unmarried	2,000,000-3,000,000 JPY	no	1	1	0	0	0	0	0	3	3	1	1	2	2	1	1	1	2	2	1	3	1	1	1	2	3
660	70	unmarried	2,000,000-3,000,000 JPY	no	0	2	0	0	2	2	1	2	1	1	1	2	1	1	1	1	1	1	1	3	1	1	1	1	1
661	48	married	7,000,000-8,000,000 JPY	no	0	0	0	0	0	0	0	0	1	1	1	1	1	1	1	1	1	1	1	1	1	1	1	1	1
662	46	married	5,000,000-6,000,000 JPY	yes	0	1	0	0	0	0	1	1	1	1	2	3	2	2	2	1	2	2	1	4	1	2	3	2	1
663	64	married	1,000,000-2,000,000 JPY	yes	1	1	0	0	0	0	1	4	1	1	1	2	1	2	3	2	1	2	1	3	1	1	1	4	1
664	62	married	less than 1,000,000 JPY	yes	0	0	0	0	0	0	0	3	1	2	2	2	2	2	1	1	1	2	1	3	1	1	1	2	1
665	67	married	4,000,000-5,000,000 JPY	yes	2	2	0	1	2	0	3	3	2	2	1	1	3	1	1	1	2	2	1	4	1	1	4	4	4
666	75	married	3,000,000-4,000,000 JPY	yes	0	0	0	0	0	0	1	2	1	1	1	1	2	1	1	1	1	1	2	1	1	1	2	1	1
667	62	unmarried	10,000,000-12,000,000 JPY	no	1	2	1	0	2	1	2	5	3	3	2	1	2	2	1	1	1	2	2	4	1	1	3	3	2
668	55	unmarried	3,000,000-4,000,000 JPY	no	0	0	0	0	0	0	0	1	1	2	1	1	1	2	1	1	1	2	1	2	1	1	2	2	1
669	44	unmarried	12,000,000-15,000,000 JPY	no	0	0	0	0	0	0	0	2	1	1	1	1	1	1	1	1	1	1	1	1	1	1	1	1	1
670	74	married	10,000,000-12,000,000 JPY	yes	0	0	0	0	0	0	1	1	1	1	1	1	1	1	1	1	1	1	1	2	1	1	2	2	1
671	71	married	2,000,000-3,000,000 JPY	yes	0	1	0	0	2	5	1	2	3	4	2	4	3	1	2	1	3	3	2	3	2	1	5	4	5
672	58	married	20,000,000 or more JPY	yes	2	1	5	3	5	3	1	5	3	3	1	2	2	1	1	1	2	2	3	3	2	1	5	4	5
673	71	married	1,000,000-2,000,000 JPY	yes	5	5	5	5	5	5	1	6	5	5	5	5	5	5	5	5	5	5	5	5	5	5	5	5	5
674	66	married	4,000,000-5,000,000 JPY	yes	2	3	3	4	5	0	2	6	1	1	2	1	3	1	1	1	3	2	1	2	1	1	4	3	3
675	48	married	10,000,000-12,000,000 JPY	yes	0	1	0	1	0	0	1	1	1	1	1	1	1	2	2	2	2	2	2	2	1	2	2	2	2
676	53	married	7,000,000-8,000,000 JPY	yes	1	1	0	1	4	2	1	4	3	2	3	3	2	2	2	1	3	4	2	5	3	3	5	4	4
677	41	unmarried	4,000,000-5,000,000 JPY	no	0	1	0	0	0	0	1	3	1	1	1	1	1	1	1	1	1	1	1	1	1	1	1	1	1
678	43	married	4,000,000-5,000,000 JPY	yes	1	1	1	0	1	0	1	3	1	1	1	1	2	2	2	1	2	2	1	3	2	1	2	2	1
679	46	married	10,000,000-12,000,000 JPY	yes	5	1	5	0	5	5	0	3	1	4	3	1	3	1	1	1	2	3	1	3	1	1	2	2	2
680	44	married	4,000,000-5,000,000 JPY	no	0	0	0	0	0	0	0	3	1	1	1	1	1	1	1	1	1	1	1	1	1	1	1	1	1
681	40	unmarried	less than 1,000,000 JPY	no	3	3	2	2	0	0	0	3	2	3	1	3	4	4	4	4	4	3	4	4	5	2	2	2	3
682	58	unmarried	1,000,000-2,000,000 JPY	no	0	0	0	0	0	0	0	3	1	1	1	1	1	1	1	2	1	2	1	1	1	1	1	1	1
683	51	unmarried	8,000,000-9,000,000 JPY	no	1	0	0	0	1	0	0	4	2	1	1	1	1	1	1	1	1	2	1	2	1	1	1	1	1
684	58	married	6,000,000-7,000,000 JPY	yes	1	3	4	1	5	1	1	4	2	3	2	3	3	3	3	3	2	2	2	2	2	1	2	3	2
685	72	married	3,000,000-4,000,000 JPY	yes	1	3	0	2	2	0	3	4	3	1	1	3	3	1	1	1	1	2	1	3	1	1	2	3	2
686	73	married	3,000,000-4,000,000 JPY	yes	2	2	1	1	1	1	1	3	2	2	2	2	2	1	1	2	1	2	1	2	1	1	1	2	2
687	66	married	6,000,000-7,000,000 JPY	no	0	0	0	0	0	0	5	3	1	1	1	1	1	1	1	1	1	1	1	1	1	1	2	4	2
688	73	married	20,000,000 or more JPY	yes	0	0	0	0	1	0	0	2	1	1	1	2	2	1	1	1	1	2	1	3	1	1	3	2	3
689	76	married	2,000,000-3,000,000 JPY	yes	1	1	1	0	1	0	1	3	3	2	1	1	3	2	1	1	1	2	2	1	1	1	4	4	5
690	56	married	4,000,000-5,000,000 JPY	no	0	0	0	0	0	0	0	1	1	1	2	1	2	2	2	1	1	2	1	2	1	1	2	1	3
691	71	unmarried	1,000,000-2,000,000 JPY	no	3	3	2	0	2	3	3	4	2	2	1	2	2	1	1	1	2	1	1	2	1	1	2	2	2
692	55	married	12,000,000-15,000,000 JPY	yes	0	1	0	0	0	0	0	2	1	2	1	2	2	1	1	1	1	2	1	1	1	1	1	1	1
693	71	married	5,000,000-6,000,000 JPY	yes	0	0	0	0	5	1	1	1	1	2	1	1	1	2	1	1	2	2	1	2	1	2	2	3	4
694	71	married	4,000,000-5,000,000 JPY	yes	0	0	0	0	0	0	0	0	1	1	1	1	1	1	1	1	1	2	1	1	1	1	1	1	1
695	56	unmarried	5,000,000-6,000,000 JPY	no	5	4	5	1	5	5	0	6	3	3	2	2	2	2	3	3	3	3	3	5	4	2	3	4	3

696	50	married	9,000,000-10,000,000 JPY	yes	0	0	0	0	0	0	0	0	1	1	1	1	1	1	1	1	1	1	1	1	1	1	2	2	2
697	69	unmarried	1,000,000-2,000,000 JPY	no	5	4	3	0	3	3	1	4	2	2	1	1	1	1	1	1	1	2	2	2	1	2	2	2	2
698	74	married	3,000,000-4,000,000 JPY	yes	0	0	0	1	1	1	2	5	3	3	1	1	3	1	1	1	1	2	1	2	1	1	3	3	3
699	54	married	5,000,000-6,000,000 JPY	no	0	0	0	0	0	0	2	4	1	1	1	2	1	2	1	1	1	2	1	2	1	1	2	2	2
700	80	married	6,000,000-7,000,000 JPY	yes	0	3	5	0	4	0	1	4	2	2	2	2	2	1	1	1	2	3	1	2	1	1	5	5	1
701	54	married	5,000,000-6,000,000 JPY	no	4	3	0	0	0	0	0	3	2	4	2	2	2	3	3	1	3	3	1	4	1	1	1	1	1
702	66	unmarried	less than 1,000,000 JPY	no	1	1	1	0	1	0	1	1	1	2	1	1	1	1	1	1	1	2	1	2	1	1	2	2	2
703	58	unmarried	7,000,000-8,000,000 JPY	no	0	1	0	1	0	0	1	3	2	2	1	2	2	1	1	1	2	2	1	2	2	1	2	1	1
704	83	married	1,000,000-2,000,000 JPY	no	3	3	4	4	4	4	3	4	2	3	4	4	4	4	4	4	4	4	4	4	3	3	3	3	3
705	40	unmarried	6,000,000-7,000,000 JPY	no	1	3	0	1	1	0	2	2	2	2	3	4	2	4	4	3	3	3	4	3	3	2	3	2	4
706	67	married	4,000,000-5,000,000 JPY	yes	1	2	2	2	3	3	3	3	2	3	2	1	2	1	1	1	2	3	1	2	1	1	3	4	2
707	52	unmarried	3,000,000-4,000,000 JPY	no	0	0	0	0	0	0	0	0	1	1	1	1	1	1	1	1	1	1	1	1	1	1	1	1	1
708	62	married	3,000,000-4,000,000 JPY	yes	0	0	0	0	0	0	1	3	1	2	1	1	1	1	2	1	2	2	1	2	1	1	1	1	1
709	64	married	4,000,000-5,000,000 JPY	no	1	1	1	0	1	1	1	2	2	2	1	1	2	2	2	1	1	2	1	2	1	1	3	2	3
710	59	married	5,000,000-6,000,000 JPY	yes	0	1	0	0	1	0	0	2	2	2	1	1	1	2	1	1	2	2	1	2	1	1	1	2	1
711	61	unmarried	3,000,000-4,000,000 JPY	no	1	1	0	1	1	0	1	3	1	1	1	1	1	1	1	1	1	2	1	2	1	1	1	1	1
712	71	married	3,000,000-4,000,000 JPY	yes	0	0	0	0	0	0	1	1	3	3	1	1	2	1	1	1	1	3	1	3	1	1	3	3	3
713	56	unmarried	3,000,000-4,000,000 JPY	yes	0	1	1	0	1	1	1	3	1	1	1	1	2	1	1	1	2	2	1	2	1	1	2	2	3
714	72	married	6,000,000-7,000,000 JPY	yes	1	1	0	0	0	0	1	2	1	2	1	1	2	1	1	1	2	2	1	3	1	1	2	3	2
715	72	married	2,000,000-3,000,000 JPY	yes	0	1	0	0	0	0	0	2	2	1	1	1	2	1	1	1	1	2	1	3	1	1	3	3	2
716	63	unmarried	4,000,000-5,000,000 JPY	no	0	1	1	1	4	0	4	3	3	2	2	2	2	2	1	1	2	3	1	5	1	1	5	5	4
717	106	unmarried	20,000,000 or more JPY	no	0	0	0	0	0	0	1	0	1	1	1	1	1	1	1	1	1	1	1	1	1	1	1	1	1
718	57	unmarried	4,000,000-5,000,000 JPY	no	0	0	0	0	0	0	0	1	2	2	2	3	2	2	3	2	2	2	3	5	2	1	2	2	2
719	76	married	2,000,000-3,000,000 JPY	yes	5	5	0	0	0	0	1	5	1	3	1	1	1	1	1	1	2	3	1	3	3	3	3	3	3
720	47	married	4,000,000-5,000,000 JPY	no	1	1	0	0	0	0	1	4	3	3	1	1	1	3	1	1	2	2	2	3	1	1	4	2	4
721	60	married	3,000,000-4,000,000 JPY	yes	1	3	1	1	1	0	1	5	1	1	1	1	1	1	1	1	1	3	1	4	1	1	3	3	3
722	46	unmarried	3,000,000-4,000,000 JPY	no	1	1	0	0	1	0	1	3	1	1	1	2	3	1	1	1	1	1	1	3	2	1	2	3	1
723	73	married	2,000,000-3,000,000 JPY	no	0	0	0	0	0	0	0	2	1	3	1	1	1	1	1	1	1	4	1	1	1	1	3	3	1
724	63	married	6,000,000-7,000,000 JPY	no	5	5	5	1	5	5	1	5	2	3	1	1	1	1	1	1	3	3	1	4	3	2	5	3	5
725	71	unmarried	20,000,000 or more JPY	no	0	5	4	5	5	0	5	3	2	1	1	3	2	1	1	1	3	3	1	3	1	3	3	3	2
726	70	married	4,000,000-5,000,000 JPY	yes	0	0	0	0	0	0	0	0	1	1	1	1	1	1	1	1	1	1	1	1	1	1	1	1	1
727	90	married	3,000,000-4,000,000 JPY	yes	2	2	5	0	0	0	3	6	1	1	1	1	3	1	1	1	1	1	1	1	1	1	1	1	1
728	70	married	3,000,000-4,000,000 JPY	yes	1	1	1	1	1	1	0	3	2	2	1	1	2	2	1	1	1	2	1	2	1	1	1	1	1
729	45	unmarried	2,000,000-3,000,000 JPY	no	0	1	0	0	0	0	2	1	2	1	1	2	1	1	1	1	1	1	1	3	1	1	1	1	1
730	86	married	4,000,000-5,000,000 JPY	yes	5	5	3	0	2	0	4	6	1	2	1	5	2	3	2	1	2	2	1	3	2	1	5	5	5
731	43	married	4,000,000-5,000,000 JPY	yes	0	0	0	0	0	0	0	2	1	2	1	1	2	1	1	1	1	2	2	2	1	1	2	2	2
732	41	unmarried	2,000,000-3,000,000 JPY	no	0	0	0	0	0	0	0	1	2	2	1	4	2	2	5	4	2	2	4	1	2	1	1	2	2
733	53	married	3,000,000-4,000,000 JPY	yes	0	3	0	3	0	0	1	3	2	2	3	1	3	3	2	2	2	3	2	3	2	1	1	2	1
734	74	married	3,000,000-4,000,000 JPY	yes	0	1	0	1	0	0	0	1	1	2	1	1	1	1	1	1	1	1	1	1	1	1	1	1	1
735	54	unmarried	less than 1,000,000 JPY	no	5	5	0	5	1	1	3	4	2	5	1	5	4	4	1	1	1	4	1	4	3	1	1	4	1
736	56	married	7,000,000-8,000,000 JPY	yes	1	1	0	1	1	0	2	4	2	3	2	2	2	1	2	2	2	2	2	2	1	1	2	3	2
737	80	married	3,000,000-4,000,000 JPY	yes	0	1	0	1	5	0	2	3	1	2	1	2	2	1	1	1	2	2	1	3	1	4	4	5	3
738	55	unmarried	3,000,000-4,000,000 JPY	no	1	1	0	1	0	2	0	1	2	2	1	2	4	3	3	1	2	3	3	4	2	2	4	2	3
739	60	married	5,000,000-6,000,000 JPY	no	0	1	0	0	0	0	2	2	1	1	1	1	2	2	1	1	2	2	1	2	1	1	1	1	1
740	80	married	20,000,000 or more JPY	no	5	5	5	5	5	5	3	3	1	1	1	1	1	1	1	1	1	2	1	1	1	1	2	1	1
741	73	married	6,000,000-7,000,000 JPY	yes	2	4	1	2	2	4	2	4	3	2	1	2	2	1	1	1	2	4	2	4	2	2	4	3	3
742	73	married	5,000,000-6,000,000 JPY	yes	1	3	0	1	1	0	1	3	2	1	1	2	2	1	1	1	3	3	2	4	2	3	3	2	2
743	77	married	7,000,000-8,000,000 JPY	yes	0	5	0	0	0	0	1	5	1	2	1	1	1	1	1	1	1	2	1	3	1	1	1	1	3
744	46	married	7,000,000-8,000,000 JPY	yes	0	0	0	0	0	0	1	2	2	1	1	2	4	2	1	2	2	3	4	4	3	1	1	1	1
745	57	married	less than 1,000,000 JPY	no	0	0	0	0	0	0	5	4	1	1	1	1	1	2	1	1	1	1	1	1	1	1	1	1	1
746	69	married	20,000,000 or more JPY	yes	0	0	0	0	0	0	0	0	1	1	1	1	1	1	1	1	1	1	1	1	1	1	1	1	1
747	57	married	7,000,000-8,000,000 JPY	yes	4	4	3	3	3	3	2	4	3	3	3	3	3	3	3	3	3	3	3	3	3	3	3	3	3
748	58	married	3,000,000-4,000,000 JPY	yes	1	4	0	3	0	0	2	4	1	2	1	2	2	1	2	2	2	2	2	4	1	1	1	1	2
749	47	married	12,000,000-15,000,000 JPY	yes	0	1	0	0	0	0	4	3	1	2	2	2	3	2	2	1	2	2	1	1	1	1	1	1	1
750	43	married	9,000,000-10,000,000 JPY	yes	1	5	0	5	0	0	1	3	2	1	1	1	1	2	2	4	2	4	4	4	1	1	3	2	1
751	59	married	7,000,000-8,000,000 JPY	yes	1	2	0	1	0	1	1	2	2	2	2	2	1	1	1	1	1	1	1	2	1	1	1	4	4
752	46	married	5,000,000-6,000,000 JPY	no	0	0	0	0	0	0	1	3	1	1	1	1	1	1	1	1	1	2	1	1	1	1	1	1	1
753	80	married	2,000,000-3,000,000 JPY	yes	0	1	0	0	0	0	1	2	1	1	1	1	1	2	1	1	1	2	1	3	1	1	3	4	3

754	52	married	4,000,000-5,000,000 JPY	yes	5	5	5	5	5	0	1	5	4	5	1	5	4	4	1	1	4	4	3	4	1	1	3	3	2
755	61	married	6,000,000-7,000,000 JPY	yes	0	1	0	1	1	0	0	2	1	1	1	1	2	1	1	1	1	2	1	1	1	1	2	2	2
756	62	married	less than 1,000,000 JPY	yes	0	0	0	0	0	0	0	1	1	1	1	1	1	1	1	1	1	1	1	1	1	1	1	1	1
757	73	married	5,000,000-6,000,000 JPY	no	3	3	0	0	2	0	2	4	4	3	3	3	4	4	4	2	3	4	4	4	3	3	3	4	4
758	51	married	8,000,000-9,000,000 JPY	yes	0	0	0	0	0	0	0	0	1	1	1	1	1	1	1	1	1	1	1	1	1	2	2	1	1
759	63	married	3,000,000-4,000,000 JPY	no	0	0	0	0	0	0	0	2	1	1	1	1	1	1	1	1	1	1	1	1	1	1	1	1	1
760	70	married	4,000,000-5,000,000 JPY	yes	1	1	0	1	1	1	1	3	2	2	1	1	2	1	1	1	2	1	1	3	1	1	3	4	2
761	63	married	5,000,000-6,000,000 JPY	yes	0	0	0	0	0	0	0	1	1	1	1	1	1	1	1	1	1	1	1	1	1	1	1	1	1
762	60	unmarried	less than 1,000,000 JPY	no	4	4	4	4	5	4	2	6	3	3	2	3	3	3	4	4	4	4	4	5	4	3	5	5	5
763	54	unmarried	10,000,000-12,000,000 JPY	no	0	0	0	0	0	0	1	3	1	1	1	1	1	1	1	1	1	1	1	1	1	1	1	1	1
764	48	married	10,000,000-12,000,000 JPY	no	1	1	2	0	0	0	1	3	1	2	1	1	1	1	1	1	1	1	1	2	1	1	2	2	2
765	67	married	4,000,000-5,000,000 JPY	yes	0	0	0	0	0	0	2	2	1	1	1	1	1	1	1	1	1	1	1	1	1	1	5	5	5
766	73	married	2,000,000-3,000,000 JPY	yes	0	1	0	0	0	0	0	1	1	1	1	2	1	1	1	1	1	1	1	2	1	1	2	2	1
767	43	unmarried	3,000,000-4,000,000 JPY	no	0	0	0	0	0	0	1	3	1	2	1	1	1	1	1	1	1	1	1	1	1	1	1	1	1
768	70	married	4,000,000-5,000,000 JPY	yes	1	1	1	1	5	3	0	4	2	2	1	1	2	2	2	1	3	3	1	4	1	1	4	5	5
769	42	married	4,000,000-5,000,000 JPY	yes	1	1	0	1	0	0	1	4	4	2	2	3	5	5	5	3	5	5	4	5	5	2	4	3	1
770	41	married	1,000,000-2,000,000 JPY	yes	0	2	4	4	4	1	2	3	3	4	4	2	3	5	4	4	4	5	3	4	4	4	3	1	4
771	60	married	7,000,000-8,000,000 JPY	yes	4	0	0	0	4	3	0	4	3	1	1	1	1	1	1	1	1	3	1	5	1	1	3	2	4
772	60	unmarried	4,000,000-5,000,000 JPY	no	0	0	0	0	0	0	0	1	3	4	2	1	2	1	1	2	2	3	2	3	2	3	1	1	1
773	55	married	3,000,000-4,000,000 JPY	no	0	0	0	0	0	0	2	3	1	1	1	1	1	1	1	1	1	1	1	1	1	1	1	1	1
774	50	married	5,000,000-6,000,000 JPY	yes	1	1	0	0	2	0	1	4	2	3	2	3	3	2	4	4	2	3	4	4	1	1	2	2	1
775	50	married	7,000,000-8,000,000 JPY	yes	0	0	0	0	0	0	0	1	1	1	1	1	1	1	1	1	1	2	1	2	1	1	2	1	1
776	46	unmarried	2,000,000-3,000,000 JPY	no	1	1	0	0	0	0	3	3	2	2	2	3	3	3	2	2	2	2	2	2	2	2	2	2	2
777	78	married	3,000,000-4,000,000 JPY	yes	1	1	1	0	5	0	2	6	1	1	1	1	1	1	1	1	1	2	1	3	2	1	3	3	1
778	72	unmarried	1,000,000-2,000,000 JPY	no	1	1	2	0	1	0	5	3	3	3	1	3	3	3	2	2	3	3	3	3	3	1	2	2	1
779	65	married	3,000,000-4,000,000 JPY	yes	1	1	0	0	4	1	0	2	2	3	2	2	3	3	3	2	2	3	2	4	2	2	4	4	5
780	53	married	8,000,000-9,000,000 JPY	yes	1	2	0	0	0	0	1	2	1	3	1	2	2	1	1	1	2	1	1	2	1	1	2	3	2
781	72	married	3,000,000-4,000,000 JPY	no	4	3	3	5	5	1	1	4	3	3	1	1	3	1	1	1	1	3	1	4	3	1	4	4	4
782	57	married	6,000,000-7,000,000 JPY	yes	0	1	0	0	0	0	1	3	1	1	1	1	1	1	1	1	1	1	1	2	1	1	1	1	1
783	56	married	4,000,000-5,000,000 JPY	yes	1	0	0	0	1	0	1	2	2	1	1	1	1	1	1	1	1	1	1	2	2	1	2	1	1
784	68	married	2,000,000-3,000,000 JPY	yes	3	0	3	1	3	2	2	3	1	1	1	2	2	1	1	1	2	1	1	3	1	1	2	2	2
785	48	married	6,000,000-7,000,000 JPY	no	0	0	0	0	0	0	2	0	1	4	1	2	2	3	1	1	1	1	1	2	1	1	1	3	1
786	71	married	2,000,000-3,000,000 JPY	yes	0	0	0	0	0	0	1	1	3	1	1	1	1	1	1	1	1	1	1	1	1	1	1	1	1
787	73	married	10,000,000-12,000,000 JPY	yes	1	0	1	1	5	0	0	4	1	1	1	1	1	1	1	1	1	1	1	2	1	1	2	2	1
788	70	unmarried	4,000,000-5,000,000 JPY	no	2	3	2	2	3	3	1	3	3	2	3	3	2	4	3	3	3	3	4	4	4	3	3	3	3
789	54	married	1,000,000-2,000,000 JPY	no	1	0	0	0	5	0	1	4	4	4	1	4	4	3	4	3	4	4	4	5	4	3	4	4	4
790	75	married	3,000,000-4,000,000 JPY	yes	2	0	1	0	5	0	1	4	3	2	1	2	2	2	1	1	2	2	1	3	1	1	3	3	3
791	41	married	5,000,000-6,000,000 JPY	yes	0	2	0	0	0	0	1	2	1	1	1	1	1	1	1	1	1	1	1	1	1	1	1	1	1
792	68	married	10,000,000-12,000,000 JPY	yes	0	1	1	2	2	3	1	3	3	3	2	2	1	1	1	1	1	3	1	3	3	2	3	2	3
793	70	married	3,000,000-4,000,000 JPY	yes	0	0	0	0	5	0	1	2	3	3	2	1	2	2	2	1	2	3	2	3	2	3	3	3	3
794	55	unmarried	8,000,000-9,000,000 JPY	yes	2	2	1	1	2	1	2	5	3	3	3	4	2	3	4	2	3	3	3	4	4	2	3	3	3
795	60	married	3,000,000-4,000,000 JPY	yes	4	5	5	5	5	4	2	3	2	5	2	3	5	1	1	1	5	1	1	1	3	1	1	5	5
796	58	married	6,000,000-7,000,000 JPY	yes	1	1	0	0	0	0	0	2	2	2	1	3	2	2	1	2	2	3	2	3	1	1	1	1	1
797	50	unmarried	4,000,000-5,000,000 JPY	yes	1	0	0	0	0	0	1	3	2	3	3	4	3	3	3	1	2	2	1	3	1	1	3	3	2
798	56	married	9,000,000-10,000,000 JPY	yes	0	0	0	0	0	0	0	1	1	3	1	1	1	1	1	1	1	2	1	4	2	1	4	4	4
799	64	married	4,000,000-5,000,000 JPY	yes	0	1	0	0	0	0	0	0	1	1	1	2	2	1	1	1	2	3	1	3	2	1	2	2	1
800	61	married	1,000,000-2,000,000 JPY	no	0	0	0	0	0	0	0	0	1	1	1	1	1	1	1	1	1	3	1	3	1	1	3	3	1
801	79	married	3,000,000-4,000,000 JPY	yes	5	3	3	1	5	2	3	6	3	4	1	3	4	2	1	1	4	5	3	5	4	3	5	5	5
802	73	married	5,000,000-6,000,000 JPY	yes	3	1	2	1	1	4	1	5	3	3	1	2	2	1	2	2	3	3	1	4	2	1	4	4	4
803	64	married	4,000,000-5,000,000 JPY	yes	0	0	0	0	0	0	0	1	1	1	1	1	1	1	1	1	1	1	1	1	1	1	4	4	4
804	42	married	10,000,000-12,000,000 JPY	yes	0	0	0	0	0	0	1	3	1	1	1	1	1	1	1	1	1	1	1	1	1	1	1	1	1
805	51	married	7,000,000-8,000,000 JPY	yes	0	1	0	0	0	0	1	4	1	1	1	1	1	1	1	1	1	2	1	4	4	1	1	4	4
806	50	married	7,000,000-8,000,000 JPY	no	0	2	0	1	0	0	1	2	3	3	3	2	2	2	2	2	2	2	2	3	2	1	2	4	3
807	50	unmarried	3,000,000-4,000,000 JPY	no	3	3	0	1	1	1	1	3	2	2	1	2	2	2	2	1	2	2	3	2	1	2	2	2	2
808	53	married	less than 1,000,000 JPY	yes	0	0	0	0	0	0	0	1	1	1	1	1	2	1	2	2	1	2	2	2	1	1	1	1	1
809	81	married	5,000,000-6,000,000 JPY	yes	1	2	4	1	0	0	1	2	1	1	1	2	2	1	1	1	3	3	1	3	1	1	4	4	4
810	51	unmarried	6,000,000-7,000,000 JPY	no	0	0	0	0	0	0	0	1	1	1	1	1	4	1	1	1	4	4	1	5	1	1	5	5	4
811	69	married	7,000,000-8,000,000 JPY	yes	1	0	1	0	2	0	1	4	1	1	1	2	2	1	1	1	2	2	1	2	1	1	1	1	1

812	65	married	3,000,000-4,000,000 JPY	yes	2	1	0	1	0	0	1	4	3	2	1	1	1	1	1	1	2	2	1	2	1	1	1	1	1
813	52	married	4,000,000-5,000,000 JPY	yes	0	0	0	0	5	0	1	4	1	2	1	2	2	1	1	1	2	3	2	3	2	1	3	3	3
814	67	married	3,000,000-4,000,000 JPY	yes	0	1	0	2	1	0	1	3	3	1	1	2	2	1	2	1	2	1	1	2	2	2	3	3	2
815	70	married	4,000,000-5,000,000 JPY	yes	0	1	0	1	1	0	2	2	4	4	1	3	3	3	3	2	3	3	3	3	3	3	5	5	4
816	51	married	7,000,000-8,000,000 JPY	yes	0	1	1	0	2	0	1	2	1	1	1	2	2	1	1	1	1	1	1	2	1	1	2	1	1
817	76	married	2,000,000-3,000,000 JPY	yes	1	2	1	1	1	1	2	5	2	2	2	2	2	2	2	1	2	3	2	4	2	2	4	4	4
818	47	married	5,000,000-6,000,000 JPY	no	0	0	0	0	0	0	1	1	1	1	1	1	1	2	2	1	1	2	1	2	1	1	2	1	2
819	71	married	2,000,000-3,000,000 JPY	yes	5	4	5	4	5	5	1	5	3	2	2	3	3	3	4	4	4	4	5	5	4	2	5	4	5
820	50	married	6,000,000-7,000,000 JPY	yes	0	1	0	0	0	0	1	1	1	1	1	1	1	1	1	1	1	1	1	1	1	1	1	1	1
821	62	married	1,000,000-2,000,000 JPY	no	2	3	0	0	0	0	2	3	2	2	1	2	2	2	1	1	1	2	1	2	1	1	1	1	1
822	74	married	20,000,000 or more JPY	yes	1	1	1	0	2	0	1	3	2	2	2	2	3	2	1	1	1	3	2	4	2	1	4	5	3
823	76	married	2,000,000-3,000,000 JPY	yes	5	5	5	5	5	0	3	6	2	1	1	2	1	1	1	1	1	2	1	2	1	1	2	2	2
824	60	unmarried	6,000,000-7,000,000 JPY	no	5	5	5	2	2	0	0	4	3	2	1	2	3	1	2	1	4	3	2	2	2	1	2	3	2
825	51	unmarried	6,000,000-7,000,000 JPY	no	0	0	0	0	0	0	0	1	2	1	1	1	4	1	1	1	4	4	1	5	1	1	4	4	4
826	41	married	5,000,000-6,000,000 JPY	no	0	2	0	5	0	0	1	6	3	3	4	4	4	4	3	4	4	3	4	4	4	1	4	4	3
827	71	unmarried	1,000,000-2,000,000 JPY	no	1	4	1	1	3	3	2	4	2	2	1	1	1	1	2	1	1	2	1	1	1	1	1	3	3
828	63	married	5,000,000-6,000,000 JPY	yes	0	1	0	0	1	0	1	3	2	2	2	3	2	3	2	2	3	3	2	4	1	1	3	4	2
829	51	unmarried	15,000,000-18,000,000 JPY	no	1	1	0	0	0	0	1	1	2	2	1	2	2	2	1	1	2	3	2	3	1	1	2	2	2
830	75	married	5,000,000-6,000,000 JPY	yes	1	3	3	3	5	5	2	5	3	2	1	2	3	2	1	1	2	3	2	3	3	1	4	5	5
831	55	married	12,000,000-15,000,000 JPY	yes	0	0	0	0	0	0	0	1	1	1	1	1	1	1	1	1	1	1	1	1	1	1	1	1	1
832	69	married	7,000,000-8,000,000 JPY	yes	1	1	5	5	5	5	0	3	3	2	1	2	2	2	2	1	2	3	1	4	3	1	4	4	4
833	42	unmarried	1,000,000-2,000,000 JPY	no	1	1	0	0	0	0	0	3	1	2	1	2	2	1	1	1	2	2	1	2	1	1	1	2	1
834	74	married	4,000,000-5,000,000 JPY	yes	5	3	5	3	5	5	5	5	5	5	4	4	4	4	3	3	5	5	4	5	5	1	3	4	4
835	52	married	18,000,000-20,000,000 JPY	yes	1	3	0	0	0	0	1	3	2	2	2	2	2	2	2	2	2	2	2	2	2	3	2	2	2
836	62	unmarried	7,000,000-8,000,000 JPY	no	0	0	0	0	0	0	0	0	1	2	1	1	1	1	1	1	1	1	1	2	1	1	1	1	1
837	52	unmarried	1,000,000-2,000,000 JPY	no	0	1	0	0	1	0	2	4	1	1	1	2	1	1	1	1	1	1	1	1	1	1	2	2	2
838	67	married	9,000,000-10,000,000 JPY	yes	0	1	0	0	0	0	1	2	1	1	3	2	1	1	1	1	1	1	1	3	1	1	4	5	5
839	67	unmarried	less than 1,000,000 JPY	no	1	5	2	5	4	3	1	2	3	2	1	2	1	3	3	1	1	1	1	1	2	4	1	2	1
840	56	unmarried	3,000,000-4,000,000 JPY	no	0	0	0	0	0	0	1	3	1	1	1	1	1	1	1	1	1	1	1	1	1	1	1	1	1
841	40	unmarried	8,000,000-9,000,000 JPY	no	0	0	0	0	0	0	0	3	1	1	1	1	1	1	1	1	1	1	1	1	1	1	1	1	1
842	40	married	15,000,000-18,000,000 JPY	yes	0	0	0	0	0	0	0	0	1	1	1	1	1	1	1	1	1	1	1	1	1	1	1	1	1
843	74	married	4,000,000-5,000,000 JPY	yes	1	1	1	1	1	0	5	4	2	3	1	2	1	1	1	1	1	2	1	2	2	1	2	3	1
844	47	married	3,000,000-4,000,000 JPY	no	0	0	0	0	0	0	0	4	3	3	3	3	3	3	3	3	3	3	3	3	3	3	3	3	3
845	45	married	4,000,000-5,000,000 JPY	yes	1	0	0	0	0	0	1	2	2	3	3	3	5	3	3	3	3	3	3	3	3	3	4	3	3
846	73	married	2,000,000-3,000,000 JPY	yes	5	3	3	3	3	3	2	4	3	3	2	2	2	3	2	2	2	2	2	2	2	2	2	2	2
847	69	married	4,000,000-5,000,000 JPY	yes	0	0	1	0	1	0	1	2	1	1	1	2	1	2	1	1	2	1	2	2	1	1	1	2	1
848	73	married	1,000,000-2,000,000 JPY	yes	1	0	0	1	2	0	1	3	1	2	1	1	1	2	1	1	1	2	1	2	1	1	2	4	4
849	43	unmarried	less than 1,000,000 JPY	no	3	3	3	3	3	3	3	2	1	1	1	1	1	1	1	1	1	1	1	1	1	1	1	1	1
850	71	married	2,000,000-3,000,000 JPY	no	0	0	0	0	0	0	0	0	1	1	1	1	1	1	1	1	3	3	3	5	2	1	1	5	5
851	48	married	7,000,000-8,000,000 JPY	yes	0	0	0	0	0	0	0	2	1	1	1	1	1	1	1	1	1	1	1	1	1	1	1	1	1
852	63	married	3,000,000-4,000,000 JPY	yes	1	2	1	1	4	1	5	5	1	1	1	2	2	1	2	1	2	2	1	3	1	1	2	4	2
853	80	married	3,000,000-4,000,000 JPY	no	1	1	0	1	1	0	2	3	2	2	1	2	1	1	1	1	1	3	1	3	1	1	4	4	4
854	68	married	4,000,000-5,000,000 JPY	yes	1	1	1	1	1	0	0	2	2	1	1	4	3	2	3	2	2	2	2	2	2	2	4	4	2
855	63	married	10,000,000-12,000,000 JPY	yes	1	1	0	0	1	0	1	2	2	2	1	1	2	1	1	1	1	2	1	2	1	1	3	2	1
856	50	married	4,000,000-5,000,000 JPY	yes	1	1	0	0	0	0	1	1	1	1	1	1	1	1	1	1	1	1	1	1	1	1	1	1	1
857	56	married	6,000,000-7,000,000 JPY	yes	1	3	0	0	1	0	0	3	3	3	1	2	1	2	1	1	2	2	1	3	1	1	1	1	1
858	57	married	3,000,000-4,000,000 JPY	yes	1	1	0	0	1	0	0	2	2	3	1	2	2	1	1	1	2	3	1	3	1	1	3	3	3
859	72	married	2,000,000-3,000,000 JPY	yes	0	0	0	1	1	0	1	1	3	4	1	3	3	1	1	1	1	1	1	2	1	1	2	1	1
860	74	unmarried	1,000,000-2,000,000 JPY	no	5	1	4	0	5	0	0	5	3	4	4	3	3	2	2	2	4	4	3	5	4	4	5	5	5
861	51	unmarried	2,000,000-3,000,000 JPY	no	0	0	0	0	0	0	0	1	1	2	1	1	1	1	1	1	2	2	1	2	1	1	1	1	1
862	63	married	3,000,000-4,000,000 JPY	yes	1	0	0	0	0	0	1	2	2	2	1	3	1	1	1	1	1	2	1	2	1	1	3	4	2
863	54	unmarried	less than 1,000,000 JPY	no	5	0	0	2	0	0	0	2	1	2	1	2	2	1	1	1	2	2	1	2	1	1	2	2	1
864	70	married	5,000,000-6,000,000 JPY	yes	0	0	0	0	1	0	1	3	1	1	1	1	1	1	1	1	1	2	1	2	1	2	2	2	2
865	53	married	10,000,000-12,000,000 JPY	yes	0	0	0	0	4	0	1	1	2	2	1	1	1	1	1	1	2	2	1	3	1	1	3	3	2
866	49	married	2,000,000-3,000,000 JPY	yes	0	0	0	0	0	0	0	0	1	2	1	2	2	1	1	1	1	3	2	1	1	1	2	2	2
867	72	married	4,000,000-5,000,000 JPY	yes	3	3	5	4	5	4	2	5	3	2	2	4	4	2	2	2	3	3	2	2	2	2	3	3	3
868	47	married	8,000,000-9,000,000 JPY	yes	0	1	0	1	0	0	5	2	2	1	1	1	2	1	1	1	1	1	1	3	1	1	1	2	1
869	40	unmarried	7,000,000-8,000,000 JPY	no	0	0	0	0	0	0	0	3	1	1	1	1	1	1	1	1	1	1	1	1	1	1	1	1	1

870	91	unmarried	1,000,000-2,000,000 JPY	no	1	1	1	1	1	1	5	4	2	2	2	2	2	2	2	2	2	2	2	2	2	2	2	2	2
871	71	married	2,000,000-3,000,000 JPY	yes	0	1	3	1	5	0	1	6	1	1	1	1	2	2	1	1	2	2	1	3	1	1	4	3	1
872	62	married	7,000,000-8,000,000 JPY	yes	3	3	5	2	5	4	2	4	2	2	1	3	3	2	1	2	4	3	2	3	2	2	4	4	2
873	54	married	8,000,000-9,000,000 JPY	yes	0	0	0	0	0	0	0	1	1	1	1	1	1	1	1	1	1	1	1	1	1	1	1	1	1
874	52	unmarried	10,000,000-12,000,000 JPY	no	1	0	0	0	1	5	0	3	1	1	1	1	2	1	1	1	2	2	1	2	1	1	2	1	4
875	44	married	8,000,000-9,000,000 JPY	no	3	3	1	2	1	3	3	4	3	1	3	3	4	4	4	3	3	4	3	3	4	3	3	3	3
876	63	married	9,000,000-10,000,000 JPY	yes	3	3	3	3	2	2	1	4	3	3	3	3	3	3	3	3	3	4	3	3	3	2	3	4	3
877	53	married	5,000,000-6,000,000 JPY	yes	0	0	0	0	0	0	3	1	1	1	1	1	1	1	1	1	1	1	1	1	1	1	1	1	1
878	53	married	18,000,000-20,000,000 JPY	yes	0	0	0	0	0	0	1	4	1	1	1	1	1	1	1	1	1	1	1	1	1	1	1	1	2
879	60	married	6,000,000-7,000,000 JPY	yes	0	1	0	0	3	0	0	1	4	2	1	1	3	2	2	1	2	4	2	4	2	1	3	5	1
880	47	unmarried	5,000,000-6,000,000 JPY	no	0	0	0	4	0	0	0	0	3	1	1	1	1	1	1	1	1	2	2	3	2	1	4	3	4
881	41	unmarried	less than 1,000,000 JPY	no	0	5	0	0	0	0	1	3	1	1	1	1	1	1	1	1	1	1	1	1	1	1	1	1	1
882	69	unmarried	1,000,000-2,000,000 JPY	yes	0	1	1	0	1	1	0	2	3	2	1	1	2	2	1	1	3	2	2	3	1	2	2	2	2
883	70	married	6,000,000-7,000,000 JPY	yes	3	5	5	0	5	0	1	4	3	2	2	3	3	2	2	1	3	3	2	3	1	3	3	3	2
884	62	married	4,000,000-5,000,000 JPY	yes	1	2	0	0	0	0	2	3	1	1	1	1	1	1	1	1	1	1	1	1	1	1	1	1	1
885	71	married	2,000,000-3,000,000 JPY	yes	0	1	0	1	0	0	2	2	2	2	1	1	1	1	1	1	1	2	1	2	1	1	2	2	2
886	51	unmarried	1,000,000-2,000,000 JPY	no	1	2	1	0	1	0	3	5	2	2	1	2	2	1	1	1	1	1	1	3	1	1	1	1	1
887	72	married	3,000,000-4,000,000 JPY	yes	5	5	5	4	5	5	5	6	5	4	5	5	3	2	1	1	2	2	1	4	3	3	5	5	5
888	46	married	3,000,000-4,000,000 JPY	yes	1	1	1	1	0	0	1	3	2	1	1	2	2	1	1	1	1	1	1	2	1	1	1	1	1
889	68	married	3,000,000-4,000,000 JPY	yes	0	0	0	0	2	0	1	4	2	3	2	2	2	1	1	1	2	2	1	1	1	1	3	4	4
890	64	married	10,000,000-12,000,000 JPY	yes	2	1	0	0	4	0	1	4	1	1	1	1	1	1	1	1	1	1	1	1	1	1	1	1	1
891	51	married	5,000,000-6,000,000 JPY	yes	0	0	0	0	0	0	0	0	1	1	1	1	1	1	1	1	1	1	1	1	1	1	1	1	1
892	60	unmarried	7,000,000-8,000,000 JPY	yes	0	1	0	1	1	1	3	4	3	3	3	3	3	2	2	1	2	3	2	3	1	1	2	3	1
893	56	married	5,000,000-6,000,000 JPY	yes	0	0	0	1	1	0	1	1	3	3	1	1	1	1	1	1	1	2	1	4	1	1	1	2	1
894	66	married	3,000,000-4,000,000 JPY	no	3	5	5	2	5	4	1	4	2	2	1	2	1	1	1	1	3	3	1	2	1	1	1	1	1
895	44	unmarried	2,000,000-3,000,000 JPY	no	5	5	5	5	5	5	1	3	1	1	1	1	3	3	3	3	3	3	3	3	3	2	3	3	3
896	62	married	15,000,000-18,000,000 JPY	yes	0	0	0	0	0	0	0	1	1	1	1	1	1	1	1	1	1	2	1	1	1	1	1	1	1
897	50	married	8,000,000-9,000,000 JPY	yes	0	0	0	0	0	0	0	2	1	1	1	1	1	1	1	1	1	1	1	1	1	1	1	1	1
898	71	married	1,000,000-2,000,000 JPY	no	0	0	0	0	0	0	0	0	1	2	1	1	1	1	1	1	2	2	2	3	3	1	1	5	5
899	41	unmarried	9,000,000-10,000,000 JPY	no	1	1	1	0	0	0	0	3	2	1	1	2	2	2	2	1	1	2	1	3	2	1	2	2	2
900	76	married	2,000,000-3,000,000 JPY	yes	0	0	0	0	1	0	1	2	1	2	1	2	1	1	1	1	2	2	1	2	1	1	2	2	2
901	52	married	20,000,000 or more JPY	yes	0	5	0	0	5	0	1	3	1	2	5	3	3	3	3	3	3	3	3	3	2	3	3	4	3
902	62	unmarried	less than 1,000,000 JPY	no	1	0	0	0	0	0	2	3	2	2	1	2	1	2	2	2	2	2	2	3	1	2	3	3	3
903	51	married	7,000,000-8,000,000 JPY	yes	5	5	5	5	5	5	1	5	2	2	2	2	2	2	2	2	2	2	3	2	3	3	3	3	3
904	49	married	9,000,000-10,000,000 JPY	yes	2	1	4	0	5	4	1	5	2	1	2	2	1	3	3	2	4	2	1	5	2	1	5	5	5
905	52	married	9,000,000-10,000,000 JPY	yes	1	0	1	0	5	0	4	5	3	3	3	3	3	3	3	3	3	3	3	3	3	3	3	3	3
906	71	married	3,000,000-4,000,000 JPY	yes	3	3	0	0	3	0	5	3	1	2	1	1	2	1	1	1	1	2	1	2	1	1	2	1	2
907	60	married	2,000,000-3,000,000 JPY	yes	5	2	0	3	4	2	1	4	2	3	1	2	2	1	1	2	3	4	3	5	2	3	3	4	1
908	63	married	3,000,000-4,000,000 JPY	yes	2	4	2	3	5	5	5	3	3	1	1	3	2	1	1	1	1	2	1	5	1	2	4	5	4
909	41	unmarried	9,000,000-10,000,000 JPY	no	1	1	2	0	0	0	1	4	1	1	1	2	1	2	2	1	1	1	1	2	1	1	2	2	2
910	71	married	2,000,000-3,000,000 JPY	yes	1	2	2	1	2	1	1	3	2	1	1	2	2	1	2	2	2	2	2	2	2	2	2	2	2
911	61	unmarried	less than 1,000,000 JPY	no	0	0	0	0	0	0	1	2	1	2	1	3	2	1	1	1	1	2	1	3	1	1	1	2	1
912	41	married	6,000,000-7,000,000 JPY	yes	1	1	0	0	0	0	0	2	1	2	2	1	3	2	2	1	1	3	1	3	1	1	1	1	1
913	52	married	5,000,000-6,000,000 JPY	yes	0	1	0	0	1	0	0	1	1	3	3	1	1	2	2	1	3	2	1	2	1	1	3	1	2
914	75	married	3,000,000-4,000,000 JPY	yes	1	3	3	0	3	0	2	4	1	1	1	1	1	1	1	1	1	1	1	2	1	1	3	4	4
915	66	married	1,000,000-2,000,000 JPY	no	5	5	5	5	5	5	1	3	1	1	1	1	1	1	1	1	1	1	1	1	1	1	1	1	1
916	51	unmarried	10,000,000-12,000,000 JPY	yes	0	4	0	4	4	0	1	3	1	1	1	1	1	1	1	1	1	2	1	4	2	1	2	2	1
917	53	unmarried	2,000,000-3,000,000 JPY	no	0	1	0	0	0	0	1	0	1	1	1	1	1	1	1	1	1	1	1	1	1	1	1	1	1
918	66	married	2,000,000-3,000,000 JPY	yes	2	0	2	1	2	0	1	2	1	2	1	3	2	1	1	1	2	2	1	2	1	1	2	2	1
919	61	married	9,000,000-10,000,000 JPY	yes	0	0	0	0	0	0	0	1	1	3	1	1	1	1	1	1	1	1	1	1	1	1	1	1	1
920	69	unmarried	2,000,000-3,000,000 JPY	no	0	0	0	0	0	0	1	2	1	1	1	1	1	1	1	1	1	1	1	1	1	1	1	1	1
921	66	married	3,000,000-4,000,000 JPY	no	2	5	5	2	5	3	1	5	2	2	1	2	1	1	1	1	2	3	1	2	1	1	1	1	1
922	58	unmarried	4,000,000-5,000,000 JPY	no	0	0	0	0	0	0	1	2	3	4	1	3	2	2	2	2	2	2	3	3	2	2	2	2	2
923	72	married	2,000,000-3,000,000 JPY	yes	0	0	0	0	0	0	0	1	1	1	1	1	1	1	1	1	1	2	1	2	1	1	1	2	1
924	66	married	3,000,000-4,000,000 JPY	yes	1	1	1	1	1	0	0	3	1	2	1	2	1	1	1	1	1	2	1	2	1	1	2	1	1
925	63	married	4,000,000-5,000,000 JPY	no	0	0	0	0	0	0	0	0	1	1	1	1	1	1	1	1	1	1	1	1	1	1	1	1	1
926	64	married	10,000,000-12,000,000 JPY	yes	0	1	0	1	1	0	3	4	3	3	1	3	3	2	2	1	3	3	3	4	1	3	3	3	3
927	71	married	3,000,000-4,000,000 JPY	yes	0	1	0	0	1	0	1	1	1	1	1	1	2	1	1	1	1	1	1	2	1	1	2	2	2

928	71	married	3,000,000-4,000,000 JPY	yes	0	1	1	0	2	2	1	3	1	1	1	1	1	1	1	1	1	1	1	1	1	1	1	1	1
929	60	unmarried	1,000,000-2,000,000 JPY	no	0	1	1	1	1	1	0	4	1	1	1	1	1	1	1	1	1	1	1	3	1	1	1	3	1
930	48	unmarried	less than 1,000,000 JPY	yes	0	0	1	0	1	0	0	3	1	2	2	1	1	2	2	1	2	3	1	3	2	1	2	3	2
931	44	married	7,000,000-8,000,000 JPY	yes	0	0	0	0	0	0	0	1	1	1	2	3	3	3	1	1	2	1	1	1	1	1	1	1	1
932	73	married	1,000,000-2,000,000 JPY	no	0	0	0	0	0	0	5	1	1	1	1	1	1	1	1	1	2	2	2	2	4	4	4	4	4
933	55	married	7,000,000-8,000,000 JPY	yes	1	1	0	0	0	0	5	3	1	1	1	1	1	1	1	1	1	1	1	1	1	1	1	1	1
934	44	unmarried	5,000,000-6,000,000 JPY	no	0	0	0	0	0	0	0	1	1	1	1	2	2	1	1	1	1	1	1	2	1	1	1	1	1
935	45	married	12,000,000-15,000,000 JPY	yes	0	1	0	0	1	0	1	2	1	1	2	2	3	3	2	1	2	2	2	3	1	1	2	2	2
936	70	married	9,000,000-10,000,000 JPY	yes	0	1	5	0	1	1	0	3	3	2	1	3	2	1	1	1	1	1	1	2	1	1	2	2	1
937	77	married	3,000,000-4,000,000 JPY	yes	5	2	5	1	5	4	3	5	3	1	1	2	1	1	1	1	1	1	2	2	1	3	5	5	5
938	55	married	18,000,000-20,000,000 JPY	yes	1	0	0	0	0	0	1	5	2	1	1	2	2	2	1	1	1	1	1	2	1	1	5	5	3
939	57	married	7,000,000-8,000,000 JPY	yes	5	5	0	0	5	0	1	4	3	3	2	3	3	3	3	3	3	3	3	3	3	2	5	5	5
940	106	married	less than 1,000,000 JPY	no	0	0	0	0	0	0	0	0	1	1	1	1	1	1	1	1	1	1	1	1	1	1	1	1	1
941	69	married	3,000,000-4,000,000 JPY	yes	1	3	1	1	1	0	2	4	2	1	1	1	1	1	1	1	1	1	1	1	1	1	2	2	2
942	41	married	2,000,000-3,000,000 JPY	no	0	0	1	1	1	3	1	2	2	3	2	2	2	2	2	1	1	2	1	2	2	2	2	2	2
943	42	married	5,000,000-6,000,000 JPY	yes	3	1	5	1	5	2	1	5	3	3	3	3	5	5	5	5	5	5	5	5	5	1	5	2	5
944	74	married	less than 1,000,000 JPY	yes	0	1	0	0	4	1	2	4	1	1	1	2	2	1	1	1	1	2	1	1	1	2	1	1	1
945	47	unmarried	8,000,000-9,000,000 JPY	no	0	0	0	0	0	0	0	3	1	1	1	1	1	1	1	1	1	1	1	1	1	1	1	1	1
946	49	married	6,000,000-7,000,000 JPY	yes	3	3	2	0	1	0	0	2	1	1	1	1	3	2	1	1	3	3	1	3	1	1	2	2	2
947	106	unmarried	18,000,000-20,000,000 JPY	yes	5	5	0	0	0	2	3	1	2	4	1	1	3	3	3	3	3	2	3	3	3	2	2	2	3
948	58	unmarried	5,000,000-6,000,000 JPY	no	2	2	2	0	0	0	0	3	2	1	1	2	2	1	1	1	2	2	2	2	1	2	3	3	3
949	73	married	1,000,000-2,000,000 JPY	yes	1	0	3	0	5	4	1	5	2	2	1	1	1	3	2	2	2	3	2	2	2	3	5	5	5
950	51	married	4,000,000-5,000,000 JPY	yes	0	0	4	0	1	0	0	0	1	1	1	1	1	1	1	1	1	2	1	2	1	1	1	2	1
951	66	unmarried	less than 1,000,000 JPY	no	1	1	4	1	4	5	1	5	3	3	2	2	2	1	1	1	2	2	1	1	1	1	1	1	1
952	44	unmarried	2,000,000-3,000,000 JPY	no	5	3	5	2	5	3	1	4	3	5	5	5	4	3	5	3	4	3	4	5	4	3	5	3	4
953	59	married	10,000,000-12,000,000 JPY	yes	1	3	3	0	2	2	0	3	2	2	1	2	3	1	1	1	1	2	1	3	1	1	4	5	2
954	48	married	7,000,000-8,000,000 JPY	yes	0	1	0	0	0	0	0	0	1	1	1	1	1	1	1	1	1	2	1	3	1	1	1	1	1
955	52	married	1,000,000-2,000,000 JPY	no	0	0	0	0	0	0	0	1	1	1	1	1	1	1	1	1	1	1	1	1	1	1	1	1	1
956	79	married	2,000,000-3,000,000 JPY	yes	0	0	0	1	1	2	1	3	2	3	1	2	2	2	1	1	2	3	2	3	2	1	4	4	4
957	50	married	6,000,000-7,000,000 JPY	no	0	1	0	1	0	0	1	3	1	2	1	1	2	1	1	1	1	2	1	2	1	1	1	2	2
958	64	unmarried	1,000,000-2,000,000 JPY	yes	1	1	1	1	1	1	2	3	1	1	1	1	1	2	1	1	1	1	1	1	1	1	1	1	1
959	49	unmarried	5,000,000-6,000,000 JPY	no	0	4	0	0	0	0	3	3	2	1	1	2	2	2	1	1	1	1	1	1	1	1	1	1	1
960	63	married	2,000,000-3,000,000 JPY	yes	1	1	0	0	0	0	1	2	2	1	1	3	3	2	2	2	1	2	3	4	3	4	4	4	4
961	59	married	18,000,000-20,000,000 JPY	yes	3	4	3	1	2	1	0	4	1	2	1	1	1	1	1	1	1	3	1	4	1	1	1	1	1
962	40	unmarried	6,000,000-7,000,000 JPY	no	0	0	0	0	0	0	0	0	5	3	3	5	5	5	5	5	5	5	5	5	5	2	1	1	1
963	70	married	3,000,000-4,000,000 JPY	yes	0	1	1	0	1	0	1	3	1	1	1	1	1	1	1	1	2	2	1	3	1	1	2	2	2
964	56	married	3,000,000-4,000,000 JPY	yes	3	3	3	5	3	0	1	4	3	3	3	3	3	3	3	3	3	3	3	3	2	2	2	2	2
965	56	unmarried	3,000,000-4,000,000 JPY	no	5	5	5	5	5	5	2	2	2	1	3	1	1	1	1	1	1	1	1	1	1	1	1	1	1
966	45	unmarried	5,000,000-6,000,000 JPY	no	0	0	0	0	0	0	0	3	1	1	1	1	1	1	1	1	1	1	1	3	1	1	1	1	1
967	87	married	2,000,000-3,000,000 JPY	no	3	4	0	3	4	2	2	3	3	3	1	2	2	1	1	1	2	2	2	2	2	2	3	3	3
968	79	married	2,000,000-3,000,000 JPY	yes	3	1	1	2	5	4	1	4	2	1	1	3	3	3	2	1	2	3	3	3	1	2	2	2	1
969	52	married	7,000,000-8,000,000 JPY	yes	1	4	1	0	5	1	2	4	2	2	1	3	3	2	2	1	2	2	2	2	2	1	2	2	1
970	57	married	7,000,000-8,000,000 JPY	no	0	0	0	0	0	0	0	3	1	1	1	1	1	1	1	1	1	4	1	1	1	1	1	1	1
971	60	married	12,000,000-15,000,000 JPY	yes	0	0	0	0	0	0	1	3	1	1	1	1	1	1	1	1	1	1	1	1	1	1	1	1	1
972	50	married	9,000,000-10,000,000 JPY	yes	0	0	3	0	5	3	1	3	1	1	1	2	2	2	2	2	3	2	4	4	4	1	5	5	5
973	77	married	4,000,000-5,000,000 JPY	yes	3	3	2	4	3	4	2	6	3	3	2	2	2	1	2	1	4	2	2	4	1	2	4	4	4
974	66	unmarried	less than 1,000,000 JPY	no	1	1	1	1	1	0	2	3	1	1	1	2	1	1	1	1	1	1	1	3	1	1	4	4	4
975	106	married	20,000,000 or more JPY	no	0	0	0	0	0	0	5	0	1	1	1	1	1	1	1	1	1	1	1	1	1	1	1	1	1
976	105	unmarried	20,000,000 or more JPY	no	0	0	0	0	0	0	0	0	1	1	1	1	1	1	1	1	1	1	1	1	1	1	1	1	1
977	42	unmarried	5,000,000-6,000,000 JPY	yes	1	3	0	0	2	1	0	3	3	4	4	3	4	4	3	3	3	4	4	3	3	3	4	2	2
978	45	married	3,000,000-4,000,000 JPY	yes	3	4	3	2	3	3	2	5	3	3	3	3	2	3	3	3	3	2	2	3	3	2	4	4	4
979	41	married	18,000,000-20,000,000 JPY	yes	0	0	0	0	0	0	0	3	1	1	1	1	1	1	1	1	1	1	1	2	1	1	3	1	3
980	75	married	1,000,000-2,000,000 JPY	yes	1	4	2	3	3	3	1	4	2	2	1	1	2	2	2	2	1	2	2	2	3	3	4	3	2
981	50	unmarried	2,000,000-3,000,000 JPY	no	5	3	5	5	5	5	2	3	2	2	2	2	2	1	1	1	1	1	1	1	1	1	1	1	1
982	46	unmarried	1,000,000-2,000,000 JPY	no	5	5	5	5	5	5	0	3	1	1	1	1	1	1	1	1	1	1	1	1	1	1	1	1	1
983	71	unmarried	3,000,000-4,000,000 JPY	yes	1	2	0	1	1	1	1	4	3	3	3	3	3	3	3	3	3	3	3	3	2	2	2	2	2
984	49	married	5,000,000-6,000,000 JPY	yes	1	1	1	0	1	2	1	2	3	2	2	2	4	3	4	2	2	4	3	4	3	3	4	4	4
985	70	married	18,000,000-20,000,000 JPY	yes	0	0	0	0	0	0	1	0	1	1	1	1	1	1	1	1	1	1	1	1	1	1	1	1	1

986	52	married	10,000,000-12,000,000 JPY	yes	0	0	0	0	0	0	0	3	5	5	5	5	5	5	5	1	5	5	5	5	1	1	5	5	5
987	50	married	20,000,000 or more JPY	yes	0	0	0	0	0	5	0	1	1	1	1	3	3	3	1	1	1	2	1	4	1	1	3	3	3
988	74	unmarried	2,000,000-3,000,000 JPY	yes	0	3	0	1	4	1	4	5	1	1	1	2	1	1	1	2	1	2	1	3	1	1	4	4	4
989	46	married	4,000,000-5,000,000 JPY	yes	0	0	0	0	0	0	1	2	1	1	1	1	1	1	1	1	1	1	1	2	1	1	1	1	1
990	61	married	4,000,000-5,000,000 JPY	no	0	0	0	0	0	0	0	3	1	1	1	1	2	2	1	1	1	1	1	1	1	1	1	1	1
991	45	married	9,000,000-10,000,000 JPY	yes	4	4	4	4	3	0	2	5	3	3	3	3	3	3	4	4	4	3	4	4	4	1	5	3	5
992	57	married	6,000,000-7,000,000 JPY	yes	0	1	0	1	1	0	3	4	1	1	1	2	1	1	1	1	1	2	1	2	1	1	3	2	3
993	72	married	2,000,000-3,000,000 JPY	yes	3	5	1	1	5	2	1	3	2	2	1	3	3	2	2	1	2	3	1	3	1	1	3	4	4
994	78	married	4,000,000-5,000,000 JPY	yes	1	5	5	5	5	5	3	6	3	5	3	3	3	3	3	3	4	4	3	5	3	5	5	3	5
995	61	married	2,000,000-3,000,000 JPY	yes	1	1	1	0	2	0	1	2	2	2	1	2	2	2	1	1	2	3	2	2	1	2	3	3	3
996	67	married	8,000,000-9,000,000 JPY	yes	1	5	0	1	5	3	2	5	2	2	1	5	5	1	1	1	1	3	4	4	1	1	5	5	5
997	61	married	1,000,000-2,000,000 JPY	yes	0	0	0	0	0	0	0	3	1	1	1	1	1	1	1	1	1	1	1	1	1	1	1	1	1
998	60	unmarried	5,000,000-6,000,000 JPY	no	2	1	1	1	1	1	1	3	1	2	1	1	2	1	1	1	2	2	1	3	1	1	3	3	3
999	66	married	4,000,000-5,000,000 JPY	yes	0	1	1	2	5	0	5	2	1	1	1	3	3	1	1	1	1	2	1	2	1	1	2	2	1
1000	45	married	7,000,000-8,000,000 JPY	yes	0	0	0	0	0	0	1	2	1	2	1	3	3	2	1	1	2	3	1	4	3	1	3	3	3
1001	52	married	5,000,000-6,000,000 JPY	yes	1	3	2	1	3	3	3	5	3	2	3	4	3	2	3	3	3	4	3	3	3	2	2	3	4
1002	58	married	5,000,000-6,000,000 JPY	yes	0	0	1	0	0	0	1	2	2	1	1	1	1	2	1	1	1	1	1	1	1	1	1	1	1
1003	70	married	3,000,000-4,000,000 JPY	no	5	5	4	1	5	2	2	5	3	3	1	4	2	1	1	1	1	2	2	3	1	1	1	1	1
1004	56	married	2,000,000-3,000,000 JPY	yes	0	0	0	0	0	0	0	0	1	1	1	1	1	1	1	1	1	1	1	1	1	1	4	4	1
1005	54	unmarried	3,000,000-4,000,000 JPY	no	0	0	0	0	0	0	1	3	2	2	1	2	2	2	1	1	2	1	1	3	1	1	2	2	1
1006	57	married	7,000,000-8,000,000 JPY	yes	1	1	4	1	5	1	1	5	2	2	1	2	3	3	2	2	3	4	2	5	2	1	4	2	3
1007	69	married	3,000,000-4,000,000 JPY	no	5	5	4	4	5	3	2	5	1	2	1	2	2	1	1	1	1	1	1	2	1	1	3	2	2
1008	55	married	7,000,000-8,000,000 JPY	no	2	1	1	1	1	0	0	5	2	3	1	2	1	2	2	2	1	2	2	4	1	1	2	2	1
1009	74	married	2,000,000-3,000,000 JPY	yes	1	1	0	1	1	0	1	4	2	2	1	1	2	1	1	1	2	2	1	3	1	1	2	3	2
1010	45	unmarried	4,000,000-5,000,000 JPY	no	4	1	1	1	0	1	2	3	1	3	1	2	4	1	1	1	4	3	1	5	1	1	1	3	1
1011	59	married	4,000,000-5,000,000 JPY	yes	0	0	0	0	0	0	0	3	1	1	1	1	1	1	1	1	1	1	2	1	1	1	1	1	2
1012	41	unmarried	10,000,000-12,000,000 JPY	no	1	0	0	0	0	0	0	1	2	2	4	2	3	2	2	1	3	2	2	2	2	1	3	2	2
1013	79	married	3,000,000-4,000,000 JPY	yes	3	3	3	3	3	1	2	4	2	2	2	1	1	1	1	1	2	2	1	2	2	2	3	3	3
1014	41	unmarried	3,000,000-4,000,000 JPY	no	0	0	0	0	0	0	0	1	1	1	1	1	1	1	1	1	1	1	1	1	1	1	1	1	1
1015	72	married	5,000,000-6,000,000 JPY	no	1	1	0	1	5	1	2	3	1	1	1	1	1	1	1	1	1	1	1	2	1	1	2	2	2
1016	70	married	8,000,000-9,000,000 JPY	yes	0	1	2	3	2	0	2	4	1	2	1	2	2	1	1	1	2	2	2	2	1	1	3	3	2
1017	44	married	6,000,000-7,000,000 JPY	yes	5	5	5	5	5	5	2	3	3	3	3	3	3	3	3	3	3	3	3	3	3	3	3	3	3
1018	67	married	1,000,000-2,000,000 JPY	yes	1	2	0	1	0	0	2	3	3	2	2	5	4	1	4	1	2	3	1	5	3	1	1	1	1
1019	71	married	less than 1,000,000 JPY	yes	1	1	0	1	1	0	2	3	2	1	2	3	2	1	3	2	2	3	2	3	1	1	2	2	1
1020	61	married	3,000,000-4,000,000 JPY	yes	0	0	0	0	0	0	1	3	1	1	1	1	1	1	1	1	1	2	1	2	1	1	2	2	2
1021	73	married	6,000,000-7,000,000 JPY	yes	0	0	0	0	0	0	1	3	3	1	1	1	1	1	1	1	1	1	1	1	1	1	1	1	1
1022	73	married	12,000,000-15,000,000 JPY	yes	0	0	0	0	1	0	0	1	1	1	1	1	1	1	1	1	2	2	1	3	1	1	2	2	2
1023	70	married	5,000,000-6,000,000 JPY	yes	2	3	0	1	4	3	2	5	3	4	1	3	3	2	1	1	3	2	1	2	1	2	3	3	4
1024	45	unmarried	3,000,000-4,000,000 JPY	no	5	5	0	0	5	0	2	3	3	3	3	3	3	1	2	2	3	4	1	4	2	3	5	5	5
1025	65	married	2,000,000-3,000,000 JPY	yes	5	5	5	5	5	5	3	5	2	3	1	1	3	1	1	1	1	2	1	5	1	1	5	5	1
1026	67	unmarried	3,000,000-4,000,000 JPY	yes	0	1	3	1	3	1	0	2	2	3	3	4	2	2	1	1	2	3	2	3	3	1	4	1	3
1027	60	married	1,000,000-2,000,000 JPY	yes	0	1	0	0	1	0	2	2	2	2	1	2	2	2	2	1	1	2	1	2	1	1	2	3	3
1028	102	unmarried	10,000,000-12,000,000 JPY	no	0	1	0	0	2	0	1	3	3	1	2	5	3	3	1	3	3	1	5	3	4	1	1	1	1
1029	54	married	5,000,000-6,000,000 JPY	yes	1	1	0	1	1	1	3	3	1	2	1	3	2	2	2	1	2	3	2	3	1	1	2	2	2
1030	45	married	4,000,000-5,000,000 JPY	no	1	0	0	0	0	0	3	1	1	1	1	1	2	2	1	1	3	2	1	2	2	2	4	3	4
1031	74	married	2,000,000-3,000,000 JPY	yes	1	1	1	4	4	4	2	4	2	2	1	4	3	3	2	2	2	1	3	3	2	3	3	3	3
1032	66	married	2,000,000-3,000,000 JPY	yes	1	1	0	1	3	0	2	4	3	3	1	1	2	1	1	1	1	3	3	3	1	1	2	4	1
1033	52	married	6,000,000-7,000,000 JPY	yes	0	1	0	0	0	0	0	3	1	1	1	3	3	1	1	1	1	2	1	2	1	1	1	1	1
1034	62	married	less than 1,000,000 JPY	yes	0	1	0	0	1	0	0	0	3	3	1	3	3	4	3	5	3	2	5	5	5	1	3	3	3
1035	62	married	7,000,000-8,000,000 JPY	yes	0	0	1	0	1	0	1	3	1	1	1	2	2	1	1	1	1	2	1	2	1	1	2	2	2
1036	66	married	4,000,000-5,000,000 JPY	yes	0	0	2	0	5	0	2	3	3	4	1	2	3	3	2	2	3	4	2	5	2	2	5	5	5
1037	59	unmarried	10,000,000-12,000,000 JPY	no	0	0	0	0	0	0	2	5	1	2	1	2	2	1	1	1	2	2	1	2	1	1	3	3	3
1038	71	married	2,000,000-3,000,000 JPY	yes	1	1	2	1	4	0	3	5	3	3	1	3	2	1	2	1	2	3	2	4	1	1	5	5	4
1039	71	married	3,000,000-4,000,000 JPY	yes	4	4	5	5	5	4	1	5	3	2	1	1	3	3	2	1	3	3	3	4	3	3	3	3	3
1040	54	married	15,000,000-18,000,000 JPY	yes	1	1	1	1	4	0	1	3	1	1	1	1	1	1	1	1	1	1	1	1	1	1	1	1	1
1041	71	married	1,000,000-2,000,000 JPY	no	1	1	5	2	5	2	1	4	2	3	5	4	1	1	2	2	2	2	2	2	2	2	5	5	5
1042	45	unmarried	12,000,000-15,000,000 JPY	no	0	5	4	0	3	4	1	4	1	5	2	5	3	2	2	2	5	2	1	2	1	1	2	2	5
1043	54	unmarried	7,000,000-8,000,000 JPY	no	0	0	0	0	0	0	0	1	1	1	1	1	1	1	1	1	1	2	2	2	1	1	3	3	3

1044	52	unmarried	1,000,000-2,000,000 JPY	no	1	1	0	0	1	0	1	5	2	1	1	1	1	1	1	1	2	2	1	2	1	1	2	5	2
1045	49	married	9,000,000-10,000,000 JPY	yes	0	1	0	4	1	0	1	4	2	2	2	3	3	3	3	2	3	2	2	3	2	1	3	3	3
1046	43	unmarried	3,000,000-4,000,000 JPY	no	0	0	0	0	0	0	0	3	3	1	3	1	1	1	1	1	1	2	1	3	3	1	1	1	1
1047	73	married	5,000,000-6,000,000 JPY	yes	5	5	5	3	5	5	2	5	2	3	1	1	3	2	1	1	2	3	1	2	1	1	1	1	1
1048	48	married	15,000,000-18,000,000 JPY	yes	3	2	2	2	3	3	2	2	3	3	3	2	4	3	3	3	3	3	3	4	3	4	3	4	3
1049	74	married	6,000,000-7,000,000 JPY	yes	1	1	0	1	0	0	1	3	2	3	1	2	1	2	2	1	2	2	1	2	1	1	5	5	3
1050	71	married	4,000,000-5,000,000 JPY	yes	0	0	0	0	0	0	0	1	1	1	1	1	1	1	1	1	1	1	1	1	1	1	1	1	1
1051	62	married	4,000,000-5,000,000 JPY	no	1	0	1	0	1	0	1	2	2	2	1	2	2	1	1	1	2	2	1	2	1	1	3	3	3
1052	47	married	9,000,000-10,000,000 JPY	no	0	0	0	0	0	0	0	0	5	4	5	3	5	5	5	4	4	5	5	4	5	5	4	4	5
1053	46	married	9,000,000-10,000,000 JPY	yes	0	0	0	0	0	0	4	2	1	1	1	2	2	2	2	2	2	2	2	2	1	1	1	1	1
1054	54	married	6,000,000-7,000,000 JPY	yes	2	2	2	0	4	3	0	2	2	1	1	3	2	2	3	2	2	2	3	3	3	2	3	3	5
1055	78	married	2,000,000-3,000,000 JPY	yes	1	1	0	0	0	0	2	6	1	1	1	3	2	1	1	1	1	1	1	2	1	1	4	4	4
1056	49	married	3,000,000-4,000,000 JPY	no	0	1	0	0	0	0	1	1	1	1	3	1	1	1	2	2	1	1	1	2	1	1	2	2	2
1057	70	unmarried	1,000,000-2,000,000 JPY	no	5	5	5	5	5	0	4	5	2	1	2	1	2	1	1	1	2	2	2	2	1	2	2	2	2
1058	55	married	10,000,000-12,000,000 JPY	yes	0	0	0	0	0	0	0	1	1	1	1	1	1	1	1	1	1	1	1	1	1	1	1	1	1
1059	49	unmarried	5,000,000-6,000,000 JPY	no	0	1	0	0	1	0	1	2	2	2	1	2	1	1	1	1	1	1	1	2	1	1	1	1	1
1060	73	married	7,000,000-8,000,000 JPY	yes	1	1	0	0	1	0	1	2	1	1	1	2	2	1	1	1	1	1	1	1	1	1	2	2	2
1061	65	unmarried	less than 1,000,000 JPY	no	1	1	1	0	1	0	0	3	1	3	3	1	2	1	1	1	2	1	1	1	1	1	1	1	1
1062	72	married	1,000,000-2,000,000 JPY	yes	5	5	5	5	5	5	2	6	4	5	4	3	5	4	4	3	4	5	4	5	4	3	5	5	5
1063	41	married	6,000,000-7,000,000 JPY	yes	0	0	0	0	0	0	2	1	1	1	1	2	1	3	2	1	2	2	1	2	1	1	1	1	2
1064	70	married	less than 1,000,000 JPY	no	1	2	3	1	5	5	3	5	3	2	2	3	3	3	3	2	3	2	2	2	3	3	4	3	3
1065	81	unmarried	2,000,000-3,000,000 JPY	yes	0	0	2	0	3	0	0	3	1	1	1	1	1	1	1	1	2	3	1	3	1	1	2	3	3
1066	40	unmarried	3,000,000-4,000,000 JPY	no	0	1	0	0	0	0	0	3	1	1	1	2	1	1	1	1	1	1	1	2	1	1	1	1	1
1067	60	unmarried	2,000,000-3,000,000 JPY	no	4	5	3	3	4	1	2	6	2	1	1	2	2	3	2	3	2	3	2	3	3	1	3	1	2
1068	59	unmarried	less than 1,000,000 JPY	no	2	2	0	1	0	0	1	4	3	1	1	3	1	2	2	2	2	1	3	3	2	1	3	3	3
1069	53	married	3,000,000-4,000,000 JPY	yes	0	0	0	0	0	0	1	1	1	1	1	1	1	1	1	1	2	2	1	3	1	1	3	2	3
1070	58	married	15,000,000-18,000,000 JPY	yes	1	1	0	0	0	0	2	2	1	1	1	3	2	1	1	1	1	1	1	1	1	1	1	1	1
1071	45	married	10,000,000-12,000,000 JPY	no	1	1	0	1	1	1	1	5	3	2	1	2	3	3	1	1	2	3	1	3	1	1	3	4	3
1072	49	married	9,000,000-10,000,000 JPY	yes	5	0	5	0	1	0	0	2	1	2	1	3	2	1	1	1	3	3	1	4	1	1	4	5	2
1073	71	married	4,000,000-5,000,000 JPY	yes	3	4	3	4	3	3	3	2	3	4	2	3	3	4	4	3	4	3	4	4	3	2	3	4	3
1074	67	married	4,000,000-5,000,000 JPY	no	1	1	1	0	2	1	3	4	2	2	1	1	2	1	1	1	2	2	1	3	1	1	2	2	3
1075	58	married	10,000,000-12,000,000 JPY	no	2	1	0	1	3	1	1	1	1	3	1	1	1	1	1	1	1	1	1	1	1	1	1	1	1
1076	71	married	2,000,000-3,000,000 JPY	yes	0	0	1	0	1	0	1	3	2	2	1	1	1	1	1	1	2	2	2	3	2	1	1	2	1
1077	75	married	8,000,000-9,000,000 JPY	yes	2	3	5	1	5	2	1	4	1	1	1	3	3	1	1	1	2	2	1	2	1	1	4	5	5
1078	106	married	less than 1,000,000 JPY	yes	0	3	0	0	0	0	1	1	1	1	1	1	1	1	1	1	1	1	1	1	1	1	1	1	1
1079	57	married	15,000,000-18,000,000 JPY	yes	5	0	0	0	0	0	0	5	1	1	1	1	1	1	1	1	1	1	1	1	1	1	1	3	1
1080	70	married	15,000,000-18,000,000 JPY	yes	5	5	5	4	5	5	2	6	2	2	1	2	2	1	2	2	1	1	1	2	1	2	2	2	2
1081	53	married	10,000,000-12,000,000 JPY	no	5	0	5	0	5	4	0	5	3	4	1	5	3	3	4	1	1	2	3	3	1	1	2	4	4
1082	63	married	20,000,000 or more JPY	yes	0	0	0	0	0	0	5	3	1	1	1	1	1	1	1	1	1	1	1	1	1	1	1	1	1
1083	57	unmarried	4,000,000-5,000,000 JPY	no	1	1	0	0	0	1	1	2	3	2	2	1	2	2	1	1	2	3	1	3	2	1	3	4	3
1084	40	unmarried	3,000,000-4,000,000 JPY	yes	0	0	0	0	0	0	3	3	2	1	1	1	3	2	2	1	2	2	2	4	1	1	2	2	4
1085	51	married	6,000,000-7,000,000 JPY	yes	1	1	0	1	1	0	1	3	2	2	1	1	2	2	2	1	2	2	1	3	1	1	2	2	3
1086	71	married	20,000,000 or more JPY	yes	0	0	0	0	0	0	0	0	1	1	1	1	1	1	1	1	1	1	1	1	1	1	1	1	1
1087	77	married	less than 1,000,000 JPY	yes	0	0	0	0	0	0	1	1	1	1	1	1	2	2	1	1	1	2	2	2	1	1	1	1	1
1088	49	married	12,000,000-15,000,000 JPY	yes	0	1	0	1	0	0	1	1	1	3	1	1	1	1	1	1	1	2	1	2	1	1	2	2	1
1089	66	married	12,000,000-15,000,000 JPY	yes	0	0	0	0	0	0	5	1	1	1	1	1	1	1	1	1	1	1	1	1	1	1	1	1	1
1090	63	married	5,000,000-6,000,000 JPY	yes	2	1	2	1	4	4	1	5	3	4	2	1	2	1	1	1	1	3	1	3	3	2	2	2	2
1091	73	married	5,000,000-6,000,000 JPY	yes	5	5	5	5	5	5	1	4	2	3	1	1	3	2	1	1	2	3	2	2	1	1	2	2	1
1092	48	unmarried	3,000,000-4,000,000 JPY	yes	0	0	0	1	1	0	3	2	3	1	2	1	2	3	1	1	3	2	1	4	1	1	3	1	1
1093	79	married	10,000,000-12,000,000 JPY	no	0	1	0	0	0	0	1	1	2	2	1	1	1	1	1	1	1	3	1	2	1	2	3	3	3
1094	44	unmarried	7,000,000-8,000,000 JPY	yes	0	0	0	0	0	0	0	2	2	1	1	1	2	1	1	1	1	1	1	2	1	1	2	2	2
1095	61	married	4,000,000-5,000,000 JPY	yes	3	3	0	1	0	0	2	4	1	1	1	1	2	1	1	1	2	3	1	3	2	2	2	3	2
1096	74	married	7,000,000-8,000,000 JPY	yes	0	0	0	0	0	0	2	2	2	1	1	1	1	1	1	1	1	1	1	1	1	1	1	1	1
1097	64	unmarried	less than 1,000,000 JPY	no	0	0	0	0	0	0	0	1	1	1	2	1	1	1	1	1	1	2	1	2	1	1	1	1	1
1098	59	married	9,000,000-10,000,000 JPY	yes	0	2	4	3	0	5	5	4	1	1	1	2	4	1	1	1	2	2	2	5	1	1	2	5	3
1099	75	married	less than 1,000,000 JPY	yes	0	1	0	0	1	0	0	1	1	1	1	1	1	2	2	1	1	2	1	2	1	2	3	3	4
1100	64	unmarried	1,000,000-2,000,000 JPY	no	5	4	5	0	0	4	1	3	2	3	1	2	4	2	2	2	2	3	2	2	3	2	2	3	2
1101	51	unmarried	10,000,000-12,000,000 JPY	no	0	1	0	0	0	0	1	1	3	3	1	2	4	1	2	2	3	3	2	2	2	1	1	1	1

1102	54	unmarried	3,000,000-4,000,000 JPY	no	0	1	0	0	0	0	0	2	2	1	1	2	2	1	1	1	2	4	2	3	2	1	3	5	3
1103	64	married	3,000,000-4,000,000 JPY	yes	5	5	1	1	5	5	0	3	3	3	1	3	3	2	2	1	2	2	2	5	1	2	4	5	5
1104	56	married	6,000,000-7,000,000 JPY	yes	0	0	0	0	0	0	1	3	1	1	1	1	1	1	1	1	1	1	1	1	1	1	1	1	1
1105	45	unmarried	less than 1,000,000 JPY	no	1	1	3	2	3	3	1	4	3	3	1	1	2	2	2	2	2	2	3	3	2	1	1	1	1
1106	51	married	10,000,000-12,000,000 JPY	yes	0	1	1	1	1	0	0	4	1	2	2	2	2	1	2	2	2	3	2	3	1	1	3	3	2
1107	72	married	2,000,000-3,000,000 JPY	yes	0	0	0	0	0	0	0	0	1	1	1	1	2	1	1	1	1	3	1	3	1	1	4	4	4
1108	62	married	6,000,000-7,000,000 JPY	no	3	1	4	3	3	2	3	3	3	3	3	3	3	3	3	3	3	3	3	3	3	3	2	3	3
1109	53	unmarried	3,000,000-4,000,000 JPY	no	0	0	0	0	0	0	0	1	1	1	1	2	2	1	1	2	1	1	2	1	2	1	2	2	2
1110	73	married	5,000,000-6,000,000 JPY	no	0	0	0	1	1	0	1	5	2	2	1	3	2	1	1	1	2	2	1	2	1	1	5	5	5
1111	68	married	1,000,000-2,000,000 JPY	yes	0	0	0	0	0	0	1	3	1	1	1	1	1	1	1	1	1	1	1	1	1	1	1	1	1
1112	51	married	12,000,000-15,000,000 JPY	no	0	0	0	0	0	0	0	0	1	1	1	1	1	1	1	1	1	2	1	2	1	1	2	1	2
1113	78	married	9,000,000-10,000,000 JPY	yes	1	0	0	0	0	0	1	3	2	1	1	1	1	1	1	1	1	2	1	2	1	1	1	2	2
1114	73	married	4,000,000-5,000,000 JPY	yes	1	0	2	1	2	0	2	3	3	3	2	2	2	2	2	2	3	2	4	4	3	2	2	4	3
1115	45	married	15,000,000-18,000,000 JPY	yes	1	3	1	1	1	2	2	4	2	2	2	2	2	3	2	2	2	2	3	3	2	1	1	3	2
1116	41	unmarried	less than 1,000,000 JPY	no	0	0	0	0	0	0	0	0	1	1	1	1	1	1	1	1	1	1	1	1	1	1	1	1	1
1117	49	married	8,000,000-9,000,000 JPY	yes	0	0	0	0	0	0	0	1	1	1	1	1	1	1	1	1	1	1	1	1	1	1	1	1	1
1118	72	married	3,000,000-4,000,000 JPY	yes	1	0	0	1	1	1	1	3	2	2	1	1	2	1	1	1	2	2	1	2	1	1	1	2	2
1119	42	married	6,000,000-7,000,000 JPY	yes	0	0	0	0	0	0	0	1	1	2	1	1	1	1	1	1	1	1	2	1	1	1	1	1	1
1120	50	married	8,000,000-9,000,000 JPY	yes	0	0	0	0	0	0	0	1	1	1	1	1	1	1	1	1	1	1	1	2	1	1	1	1	1
1121	76	married	5,000,000-6,000,000 JPY	yes	0	0	0	0	0	0	2	5	2	1	1	1	1	1	1	1	1	2	1	3	1	1	3	1	1
1122	53	unmarried	less than 1,000,000 JPY	no	0	0	1	0	0	1	0	2	2	3	1	2	1	1	1	1	3	2	1	3	1	1	2	2	2
1123	52	married	7,000,000-8,000,000 JPY	yes	1	2	0	2	2	0	1	4	1	1	1	3	1	2	2	1	1	2	3	2	2	2	2	2	1
1124	79	married	3,000,000-4,000,000 JPY	yes	5	0	0	0	0	0	2	3	1	2	1	1	1	1	1	1	1	2	1	3	1	1	5	5	5
1125	72	married	1,000,000-2,000,000 JPY	yes	1	3	1	0	5	3	1	3	1	1	1	3	2	1	1	1	1	1	2	3	1	1	3	5	3
1126	55	unmarried	5,000,000-6,000,000 JPY	no	0	2	1	1	3	0	0	1	1	2	1	2	2	3	1	1	1	4	2	5	5	1	5	5	1
1127	47	married	5,000,000-6,000,000 JPY	yes	3	4	0	4	1	0	2	6	5	3	3	5	5	3	3	2	3	3	5	5	5	2	2	4	1
1128	55	married	9,000,000-10,000,000 JPY	yes	2	3	3	4	2	2	3	4	2	3	3	3	3	3	4	2	3	2	3	3	1	4	2	4	3
1129	43	unmarried	5,000,000-6,000,000 JPY	no	3	4	3	3	3	4	1	4	3	3	3	3	3	3	3	3	3	3	3	3	2	2	2	2	3
1130	51	married	10,000,000-12,000,000 JPY	yes	1	1	0	0	1	0	1	2	2	2	1	1	1	1	1	1	1	1	1	2	1	1	1	1	1
1131	50	married	7,000,000-8,000,000 JPY	yes	0	0	0	0	0	0	2	3	1	1	1	1	1	1	1	1	1	1	1	1	1	1	1	1	1
1132	59	married	7,000,000-8,000,000 JPY	yes	1	1	0	5	1	1	2	3	3	2	2	3	3	1	1	1	3	3	3	3	3	3	3	3	3
1133	71	married	4,000,000-5,000,000 JPY	yes	1	1	4	5	5	0	0	2	1	2	1	2	3	2	2	4	2	2	2	2	4	2	1	1	2
1134	72	married	2,000,000-3,000,000 JPY	yes	0	1	5	3	5	5	2	3	3	2	1	2	2	4	3	3	2	2	4	4	3	3	4	4	4
1135	47	unmarried	2,000,000-3,000,000 JPY	no	0	2	1	1	2	0	1	6	3	2	3	3	4	2	3	4	2	3	2	3	3	4	1	1	1
1136	75	married	6,000,000-7,000,000 JPY	no	0	0	0	0	0	0	1	3	1	1	1	1	1	1	1	1	1	1	1	2	1	1	1	2	2
1137	52	married	6,000,000-7,000,000 JPY	yes	0	1	0	1	0	0	1	3	2	2	1	2	2	2	2	2	2	2	2	3	3	1	2	2	2
1138	79	married	2,000,000-3,000,000 JPY	yes	0	1	1	0	4	0	3	3	2	1	1	1	1	1	1	1	1	2	1	1	1	1	1	1	1
1139	40	married	4,000,000-5,000,000 JPY	yes	0	0	0	0	1	1	1	5	1	5	5	4	4	1	1	1	3	5	3	5	3	2	1	5	1
1140	62	married	7,000,000-8,000,000 JPY	yes	3	0	0	0	1	1	1	3	2	1	1	4	2	2	2	1	3	3	2	2	1	2	3	2	2
1141	71	married	4,000,000-5,000,000 JPY	yes	1	1	3	1	4	2	1	3	2	4	1	1	1	2	2	2	2	3	2	3	2	2	2	2	3
1142	50	married	4,000,000-5,000,000 JPY	yes	1	3	4	2	3	2	1	3	1	1	2	1	1	1	1	1	1	2	1	3	1	1	2	1	3
1143	46	unmarried	3,000,000-4,000,000 JPY	no	0	0	0	0	0	0	0	2	1	1	1	1	1	1	1	1	1	1	1	1	1	1	1	1	1
1144	49	unmarried	1,000,000-2,000,000 JPY	no	0	1	0	0	5	0	0	3	1	2	1	3	3	2	1	1	3	3	1	3	2	1	2	1	1
1145	53	married	7,000,000-8,000,000 JPY	yes	2	4	4	2	2	3	1	4	3	4	3	4	3	4	4	3	3	4	4	5	3	3	4	4	4
1146	74	married	7,000,000-8,000,000 JPY	yes	0	1	0	1	1	1	0	2	2	1	1	1	2	1	1	1	1	1	1	2	1	1	1	2	2
1147	40	unmarried	5,000,000-6,000,000 JPY	no	0	0	0	0	0	0	0	6	1	1	1	1	1	1	1	1	1	1	1	1	1	1	1	1	1
1148	47	unmarried	5,000,000-6,000,000 JPY	no	0	0	0	0	0	0	0	1	1	1	1	1	1	1	1	1	1	2	1	1	1	1	1	1	1
1149	63	married	9,000,000-10,000,000 JPY	yes	2	5	3	3	3	5	5	5	3	3	2	3	3	3	3	2	3	2	2	3	2	2	4	3	3
1150	62	married	12,000,000-15,000,000 JPY	yes	0	0	0	0	0	0	0	0	1	1	1	1	2	1	1	1	1	1	1	1	1	1	2	3	3
1151	57	unmarried	3,000,000-4,000,000 JPY	no	1	1	0	1	0	0	2	3	2	2	2	1	2	2	2	1	1	2	1	2	1	1	1	2	2
1152	68	unmarried	3,000,000-4,000,000 JPY	no	0	1	0	0	0	0	0	3	2	1	1	1	1	1	1	1	1	2	1	2	1	1	2	2	2
1153	56	married	7,000,000-8,000,000 JPY	yes	1	3	0	0	1	3	3	3	3	3	3	3	3	3	3	3	3	3	3	3	3	3	3	3	4
1154	49	unmarried	4,000,000-5,000,000 JPY	no	0	0	0	0	0	0	0	3	1	1	1	1	1	1	1	1	1	1	1	1	1	1	1	1	1
1155	63	married	2,000,000-3,000,000 JPY	no	5	5	5	0	4	0	3	5	3	4	1	1	3	3	3	1	3	3	1	2	1	1	3	1	3
1156	40	married	9,000,000-10,000,000 JPY	no	0	0	0	0	0	0	4	2	1	1	1	2	2	2	1	1	1	2	1	1	1	1	1	1	1
1157	44	married	2,000,000-3,000,000 JPY	yes	0	0	0	0	0	0	2	2	1	1	1	1	1	1	1	1	1	1	1	1	1	1	1	1	1
1158	72	married	20,000,000 or more JPY	yes	1	1	0	1	1	2	2	3	1	2	1	2	2	1	2	1	2	2	2	3	1	2	2	2	3
1159	40	unmarried	4,000,000-5,000,000 JPY	no	0	0	0	0	0	0	0	3	1	1	1	1	1	1	1	1	1	1	3	1	1	1	1	3	1

1160	71	married	4,000,000-5,000,000 JPY	yes	1	2	1	1	1	0	1	3	2	2	2	2	2	2	2	3	2	2	3	2	2	2	3	2	2
1161	45	unmarried	1,000,000-2,000,000 JPY	no	1	1	0	1	0	0	2	3	3	3	3	4	2	3	2	2	4	3	3	5	3	3	4	5	3
1162	71	married	2,000,000-3,000,000 JPY	yes	1	1	1	1	1	1	1	3	2	2	2	2	2	2	2	2	2	2	2	2	2	2	2	2	2
1163	62	unmarried	2,000,000-3,000,000 JPY	no	2	2	2	2	2	2	1	4	2	1	1	2	2	1	1	1	1	2	1	2	1	1	1	1	1
1164	72	married	3,000,000-4,000,000 JPY	no	5	5	5	5	5	5	5	6	3	1	1	4	3	1	2	1	3	3	3	3	3	3	3	3	3
1165	72	married	5,000,000-6,000,000 JPY	yes	1	0	0	0	1	0	2	2	1	1	1	1	1	1	1	1	1	1	1	2	1	2	2	2	2
1166	53	married	less than 1,000,000 JPY	yes	1	1	0	1	0	0	1	4	1	1	1	1	2	2	2	1	2	2	2	5	2	1	1	1	1
1167	41	unmarried	4,000,000-5,000,000 JPY	no	0	0	0	0	0	0	1	0	1	1	1	1	1	1	1	1	1	1	1	1	1	1	1	1	1
1168	74	married	4,000,000-5,000,000 JPY	yes	0	0	0	0	0	0	0	3	2	2	1	3	3	1	1	1	1	1	1	1	1	1	1	1	1
1169	42	unmarried	less than 1,000,000 JPY	no	1	2	1	1	3	0	3	4	2	2	2	2	3	2	2	4	3	3	3	3	3	3	3	3	3
1170	77	married	5,000,000-6,000,000 JPY	yes	1	2	0	1	2	0	1	4	1	1	1	2	1	1	1	1	2	1	1	3	1	1	2	4	3
1171	40	married	9,000,000-10,000,000 JPY	no	0	0	0	0	0	0	4	2	1	1	1	2	2	1	1	1	1	2	1	1	1	1	1	1	1
1172	70	married	4,000,000-5,000,000 JPY	yes	1	1	1	0	1	1	0	1	2	2	4	2	3	4	3	4	3	3	3	3	3	3	4	4	4
1173	49	unmarried	1,000,000-2,000,000 JPY	no	0	0	0	0	0	0	0	3	1	2	1	1	1	1	1	1	1	1	1	2	1	1	1	1	1
1174	59	married	12,000,000-15,000,000 JPY	yes	0	0	0	0	0	0	0	2	1	2	1	2	1	2	2	1	1	1	1	1	2	2	2	2	1
1175	67	married	3,000,000-4,000,000 JPY	yes	1	1	0	1	1	0	1	3	3	1	1	2	2	1	1	1	2	3	1	3	1	3	3	3	3
1176	69	married	3,000,000-4,000,000 JPY	yes	1	1	1	1	1	5	1	3	2	2	1	1	2	2	2	1	1	2	1	2	1	1	3	3	4
1177	47	married	4,000,000-5,000,000 JPY	yes	0	1	0	0	0	0	2	3	1	1	1	1	2	3	3	1	1	2	1	1	1	1	3	3	1
1178	59	married	10,000,000-12,000,000 JPY	yes	0	1	0	1	0	0	1	3	1	1	1	1	2	1	1	1	1	1	1	3	1	1	1	1	1
1179	77	married	less than 1,000,000 JPY	yes	0	1	1	0	1	0	1	1	1	1	1	2	2	1	1	1	2	2	1	2	1	1	4	5	4
1180	61	married	12,000,000-15,000,000 JPY	yes	2	4	1	1	2	1	1	4	3	5	3	4	4	1	4	4	3	3	2	3	2	2	3	3	3
1181	68	married	6,000,000-7,000,000 JPY	yes	0	0	0	0	0	0	1	2	1	1	1	1	1	1	1	1	1	1	1	1	1	1	1	1	1
1182	40	married	4,000,000-5,000,000 JPY	yes	1	0	0	0	0	0	1	2	1	2	1	3	2	1	1	1	1	1	1	2	2	2	2	1	1
1183	75	married	5,000,000-6,000,000 JPY	yes	0	1	0	1	0	0	1	3	1	2	2	2	2	1	1	1	2	2	2	2	1	1	2	2	2
1184	47	married	12,000,000-15,000,000 JPY	yes	0	0	0	0	0	0	0	0	1	1	1	1	1	1	1	1	1	3	2	1	1	1	2	2	2
1185	58	married	10,000,000-12,000,000 JPY	no	0	0	0	0	0	0	0	1	1	1	1	1	1	1	1	1	1	1	1	1	1	1	1	1	1
1186	62	married	7,000,000-8,000,000 JPY	yes	1	2	1	1	3	0	2	5	2	2	1	2	2	2	1	1	2	3	3	4	2	1	4	3	3
1187	48	married	5,000,000-6,000,000 JPY	yes	5	1	3	1	3	2	1	3	2	3	2	3	4	2	4	3	3	3	3	4	4	2	4	4	4
1188	60	married	10,000,000-12,000,000 JPY	no	0	0	0	0	0	0	1	2	1	2	1	1	2	2	2	1	1	3	2	3	3	3	3	3	2
1189	54	married	8,000,000-9,000,000 JPY	yes	1	1	0	0	0	0	0	4	2	2	1	1	2	2	2	2	3	3	2	3	2	2	2	3	2
1190	60	unmarried	3,000,000-4,000,000 JPY	yes	1	1	1	1	1	0	1	4	2	1	1	1	2	1	2	1	2	3	2	3	1	1	2	1	3
1191	57	unmarried	1,000,000-2,000,000 JPY	no	0	0	0	0	0	0	0	1	2	1	1	1	2	1	1	1	1	2	1	1	1	1	1	1	1
1192	66	married	4,000,000-5,000,000 JPY	yes	0	1	0	1	0	0	1	2	1	1	1	1	2	2	1	2	1	1	2	2	2	1	1	3	1
1193	52	married	4,000,000-5,000,000 JPY	no	0	0	1	0	1	1	0	2	2	2	1	3	2	2	1	1	3	2	2	2	2	2	4	3	2
1194	79	married	1,000,000-2,000,000 JPY	yes	1	1	1	3	3	0	2	4	3	3	1	3	3	1	1	1	2	1	1	1	1	1	3	3	4
1195	73	married	9,000,000-10,000,000 JPY	yes	0	0	0	0	0	0	5	1	2	2	1	1	2	1	1	1	2	2	1	1	1	1	1	2	1
1196	63	married	4,000,000-5,000,000 JPY	yes	0	1	0	0	1	0	1	3	1	1	1	1	1	1	1	1	1	2	1	1	1	1	2	2	2
1197	60	unmarried	3,000,000-4,000,000 JPY	no	0	0	0	0	0	0	1	1	1	1	1	1	1	1	1	1	1	1	1	1	1	1	1	1	1
1198	77	unmarried	3,000,000-4,000,000 JPY	no	3	3	4	0	4	4	4	5	2	2	1	3	2	1	2	2	2	3	2	2	1	1	2	4	4
1199	46	married	4,000,000-5,000,000 JPY	yes	0	0	0	0	0	0	1	3	1	1	1	1	1	1	1	1	1	1	1	1	1	1	1	1	1
1200	50	married	6,000,000-7,000,000 JPY	yes	0	0	0	0	0	0	0	0	2	2	2	1	2	2	2	2	2	2	2	2	1	1	2	2	1
1201	73	married	20,000,000 or more JPY	yes	3	2	1	4	5	0	1	4	3	4	1	4	3	1	2	1	2	4	1	4	1	1	4	4	4
1202	61	unmarried	2,000,000-3,000,000 JPY	no	1	1	0	1	1	0	2	5	2	2	2	2	2	4	4	4	5	4	5	5	5	1	5	5	5
1203	62	married	5,000,000-6,000,000 JPY	yes	0	0	0	0	0	0	1	3	1	1	1	1	1	1	1	1	1	1	1	1	1	1	1	1	1
1204	81	married	2,000,000-3,000,000 JPY	yes	1	1	1	0	4	0	5	6	3	1	1	4	1	1	1	1	1	3	1	1	1	1	1	1	1
1205	64	married	9,000,000-10,000,000 JPY	yes	5	1	2	0	5	0	3	3	2	2	1	3	2	2	2	2	4	5	4	5	3	1	4	4	3
1206	53	unmarried	4,000,000-5,000,000 JPY	no	5	0	0	5	5	0	1	4	1	1	1	2	2	1	3	3	1	4	3	5	1	1	5	5	5
1207	71	married	4,000,000-5,000,000 JPY	yes	0	0	0	0	0	0	1	1	2	2	1	1	1	1	1	1	1	2	1	2	2	2	2	3	3
1208	41	unmarried	less than 1,000,000 JPY	no	0	0	0	0	0	0	0	0	1	1	1	1	1	1	1	1	1	1	1	1	1	1	1	1	1
1209	55	married	12,000,000-15,000,000 JPY	yes	3	2	3	4	1	1	2	2	3	2	1	1	1	1	2	2	2	2	1	2	2	2	2	2	3
1210	71	married	2,000,000-3,000,000 JPY	yes	1	1	1	1	1	1	3	4	2	1	1	2	2	1	1	1	2	2	1	3	1	1	3	4	4
1211	71	married	2,000,000-3,000,000 JPY	yes	1	1	3	1	4	0	1	6	1	1	1	1	2	2	1	1	1	2	1	3	1	1	3	3	2
1212	49	unmarried	5,000,000-6,000,000 JPY	no	3	2	2	3	4	4	3	4	3	3	3	3	3	3	3	1	3	3	1	3	2	2	2	2	1
1213	47	unmarried	6,000,000-7,000,000 JPY	no	1	0	0	0	1	0	1	2	2	3	1	5	2	2	3	1	2	1	3	5	2	2	2	2	2
1214	58	married	6,000,000-7,000,000 JPY	yes	0	1	0	0	0	0	5	2	1	1	1	1	2	1	1	1	1	1	1	2	1	1	1	2	1
1215	71	married	2,000,000-3,000,000 JPY	yes	0	0	0	0	5	0	2	3	2	2	1	1	1	1	1	1	1	2	1	2	1	1	1	1	1
1216	77	married	4,000,000-5,000,000 JPY	yes	1	2	0	0	3	0	2	5	3	3	1	2	3	1	2	1	2	2	2	2	1	1	2	2	2
1217	63	married	2,000,000-3,000,000 JPY	yes	0	3	5	2	5	0	1	2	1	1	1	2	2	1	2	1	2	2	2	5	1	1	5	5	5

1218	62	married	5,000,000-6,000,000 JPY	yes	0	1	0	1	1	0	1	3	4	2	1	3	3	4	2	1	3	5	3	4	3	1	4	4	2
1219	59	unmarried	8,000,000-9,000,000 JPY	no	0	0	0	0	0	0	1	1	1	1	1	2	1	1	1	1	2	2	1	2	1	1	2	2	2
1220	41	unmarried	3,000,000-4,000,000 JPY	no	2	1	0	0	0	0	0	3	1	1	1	4	1	1	2	1	1	1	1	2	1	1	2	1	3
1221	46	married	10,000,000-12,000,000 JPY	yes	1	1	0	0	0	0	1	3	1	1	1	1	1	1	1	1	1	1	1	1	1	1	1	1	1
1222	61	married	2,000,000-3,000,000 JPY	yes	3	2	3	5	5	1	4	4	4	3	3	3	3	2	1	1	3	3	3	5	4	2	5	5	5
1223	42	married	2,000,000-3,000,000 JPY	yes	0	0	0	0	0	0	1	2	1	1	1	1	1	1	1	1	1	1	1	1	1	1	1	1	1
1224	72	married	less than 1,000,000 JPY	no	0	0	0	0	0	0	1	3	1	1	1	1	2	1	1	1	1	2	1	2	1	1	2	2	2
1225	40	unmarried	4,000,000-5,000,000 JPY	no	0	0	0	0	0	0	1	1	1	1	1	1	2	1	1	1	2	1	1	1	1	1	1	1	1
1226	64	unmarried	less than 1,000,000 JPY	no	0	0	0	0	1	0	3	3	1	1	1	2	1	2	2	1	1	2	1	2	1	2	2	2	2
1227	76	married	3,000,000-4,000,000 JPY	yes	1	1	1	1	2	1	1	3	2	4	3	2	2	2	2	1	3	3	2	3	2	2	4	1	3
1228	50	unmarried	4,000,000-5,000,000 JPY	no	0	0	1	0	1	1	0	3	1	1	1	1	2	2	2	1	2	2	2	3	1	1	1	3	2
1229	74	married	1,000,000-2,000,000 JPY	yes	5	3	3	5	4	3	1	5	3	3	3	3	3	2	3	3	3	3	3	3	3	3	3	4	4
1230	64	married	7,000,000-8,000,000 JPY	yes	2	2	0	2	2	2	0	3	1	2	1	2	2	2	1	1	2	2	1	4	1	1	1	1	1
1231	74	married	4,000,000-5,000,000 JPY	yes	2	0	0	0	2	0	0	3	2	1	1	1	1	1	1	1	1	1	1	1	1	1	3	3	3
1232	71	married	7,000,000-8,000,000 JPY	yes	1	1	0	1	1	1	1	3	1	1	1	1	1	1	1	1	2	2	1	3	1	1	2	2	3
1233	51	unmarried	20,000,000 or more JPY	no	5	5	5	5	5	5	0	0	3	3	3	3	3	3	3	3	3	3	3	3	3	3	3	3	3
1234	49	married	10,000,000-12,000,000 JPY	yes	1	3	3	1	1	0	1	3	2	2	2	3	3	3	1	1	1	2	2	5	1	1	4	2	1
1235	49	married	7,000,000-8,000,000 JPY	yes	0	0	0	0	0	0	2	3	1	3	1	2	1	1	1	1	1	1	1	2	1	1	1	1	1
1236	50	married	8,000,000-9,000,000 JPY	yes	0	0	0	0	1	0	2	2	1	4	1	2	2	2	2	1	3	3	2	3	1	1	3	4	4
1237	58	married	10,000,000-12,000,000 JPY	yes	3	0	0	0	0	0	1	3	2	2	1	1	1	1	1	1	1	2	1	1	1	1	2	2	2
1238	70	married	18,000,000-20,000,000 JPY	yes	0	1	0	1	1	1	0	0	2	2	1	2	2	2	1	1	2	2	1	1	1	1	1	2	1
1239	51	unmarried	5,000,000-6,000,000 JPY	no	1	1	0	1	0	0	0	3	3	1	1	1	1	2	2	1	1	1	1	1	1	1	1	1	1
1240	53	married	5,000,000-6,000,000 JPY	yes	0	0	0	1	1	0	3	1	1	1	1	1	2	2	1	1	1	2	1	3	1	1	2	3	2
1241	64	married	less than 1,000,000 JPY	yes	0	2	0	1	0	0	1	4	3	3	2	3	3	3	3	2	3	3	3	3	3	3	3	3	3
1242	44	unmarried	7,000,000-8,000,000 JPY	no	0	0	0	0	0	0	1	1	1	1	1	1	1	1	1	1	1	1	1	1	1	1	1	1	1
1243	48	married	7,000,000-8,000,000 JPY	no	0	1	0	0	5	0	1	2	1	1	1	1	1	1	1	1	1	1	1	2	1	1	1	2	1
1244	80	married	4,000,000-5,000,000 JPY	yes	0	0	0	0	1	1	1	1	1	1	2	1	2	2	1	1	1	1	1	2	1	1	2	3	2
1245	52	unmarried	less than 1,000,000 JPY	no	0	0	0	0	0	0	2	3	2	1	1	1	2	1	1	1	1	2	1	2	1	1	1	1	1
1246	40	married	4,000,000-5,000,000 JPY	yes	5	5	5	5	5	5	1	1	3	1	2	3	3	3	3	4	3	4	3	4	2	1	1	2	1
1247	63	married	6,000,000-7,000,000 JPY	yes	0	0	0	0	0	0	0	1	1	1	1	1	1	1	1	1	1	1	1	1	1	1	1	1	1
1248	55	unmarried	7,000,000-8,000,000 JPY	no	0	0	0	0	0	0	3	4	2	1	1	1	1	1	1	1	1	1	1	1	1	1	1	1	1
1249	59	married	8,000,000-9,000,000 JPY	yes	0	0	0	0	0	0	0	1	1	1	1	1	1	1	1	1	1	1	1	1	1	1	1	1	1
1250	74	married	1,000,000-2,000,000 JPY	no	0	0	0	0	0	0	1	1	1	1	1	1	1	2	2	2	2	2	2	2	2	1	3	2	2
1251	74	married	2,000,000-3,000,000 JPY	yes	0	0	1	0	2	0	4	2	2	3	1	1	2	1	1	1	1	3	1	2	2	1	5	5	4
1252	47	unmarried	4,000,000-5,000,000 JPY	no	0	0	0	0	0	0	0	3	1	1	1	1	1	1	1	1	1	1	1	1	1	1	1	1	1
1253	40	married	9,000,000-10,000,000 JPY	yes	0	0	0	0	0	0	0	1	1	1	1	1	1	1	1	1	1	1	1	1	1	1	1	1	1
1254	61	married	6,000,000-7,000,000 JPY	yes	0	0	0	0	0	0	4	3	2	1	1	1	2	1	1	1	1	1	1	1	1	1	1	2	2
1255	63	married	20,000,000 or more JPY	no	0	1	0	0	1	0	1	2	1	1	1	1	1	1	1	1	1	1	1	2	1	1	2	2	2
1256	72	unmarried	3,000,000-4,000,000 JPY	yes	0	1	0	0	1	0	1	1	1	1	1	2	2	1	1	1	1	2	1	3	1	1	3	3	2
1257	50	unmarried	5,000,000-6,000,000 JPY	yes	2	1	3	1	1	3	1	5	3	4	2	5	5	3	3	1	3	1	2	5	3	1	4	4	5
1258	80	married	1,000,000-2,000,000 JPY	yes	0	0	0	0	5	0	2	2	1	2	1	1	1	1	1	1	1	2	1	1	1	1	3	3	3
1259	77	married	2,000,000-3,000,000 JPY	yes	0	0	0	0	5	0	1	2	1	3	3	1	1	1	2	1	1	2	1	3	1	1	4	1	1
1260	81	married	3,000,000-4,000,000 JPY	yes	0	1	1	5	1	0	2	4	1	2	1	1	2	1	1	1	1	2	1	3	1	1	3	4	4
1261	42	married	10,000,000-12,000,000 JPY	yes	0	1	0	0	1	0	1	3	1	2	1	1	1	1	1	1	1	1	1	2	1	1	1	1	1
1262	67	married	4,000,000-5,000,000 JPY	yes	0	2	0	0	2	0	2	5	1	1	1	3	2	2	2	1	1	1	2	2	1	2	2	3	3
1263	54	unmarried	12,000,000-15,000,000 JPY	no	5	3	0	5	0	0	1	5	2	3	1	5	3	2	3	2	3	3	3	4	3	3	3	3	3
1264	64	married	5,000,000-6,000,000 JPY	yes	0	0	0	0	0	0	0	1	2	1	1	1	2	1	2	1	2	2	1	3	1	2	1	1	1
1265	76	married	3,000,000-4,000,000 JPY	yes	0	1	0	1	1	0	0	0	1	2	2	1	2	1	1	1	1	3	1	3	2	2	4	5	5
1266	40	married	6,000,000-7,000,000 JPY	no	0	1	0	0	0	0	0	2	1	1	1	1	1	1	1	1	1	1	1	1	1	1	1	1	1
1267	40	unmarried	less than 1,000,000 JPY	yes	2	1	2	2	4	2	3	3	3	3	3	3	3	3	3	3	3	3	3	3	3	3	3	3	3
1268	54	married	4,000,000-5,000,000 JPY	yes	1	1	0	1	0	0	0	3	2	2	3	3	2	3	3	1	2	2	3	3	2	2	1	1	1
1269	66	married	less than 1,000,000 JPY	yes	0	0	0	0	0	0	0	0	1	1	1	1	1	1	1	1	1	1	1	1	1	1	1	1	1
1270	72	married	3,000,000-4,000,000 JPY	yes	5	5	0	2	5	5	3	5	1	2	1	1	2	1	1	1	1	1	1	2	1	1	3	4	2
1271	49	married	15,000,000-18,000,000 JPY	yes	0	0	0	0	0	0	1	1	1	1	1	1	1	1	1	1	1	2	1	1	1	1	1	1	1
1272	60	married	9,000,000-10,000,000 JPY	yes	1	1	0	0	1	0	1	2	2	3	2	3	3	3	3	2	3	3	3	3	2	2	4	4	3
1273	71	married	2,000,000-3,000,000 JPY	yes	5	5	5	5	5	4	4	6	4	3	2	2	3	2	2	2	2	3	3	3	2	2	3	2	3
1274	76	married	3,000,000-4,000,000 JPY	yes	1	1	1	2	2	2	5	3	2	3	4	3	2	3	3	3	2	3	4	3	3	2	2	2	2
1275	68	married	2,000,000-3,000,000 JPY	yes	0	1	0	0	1	1	2	3	1	2	1	2	2	1	1	1	2	2	1	3	2	1	2	2	2

1276	48	married	20,000,000 or more JPY	yes	5	5	5	2	5	4	5	5	2	1	1	3	2	2	3	1	1	1	3	5	5	5	4	4	5
1277	54	married	10,000,000-12,000,000 JPY	yes	0	0	0	0	0	0	0	0	1	1	1	1	1	1	1	1	1	1	1	1	1	1	1	1	1
1278	73	married	3,000,000-4,000,000 JPY	yes	0	0	0	0	0	0	2	1	1	1	1	1	1	2	1	1	1	1	1	1	1	1	1	1	1
1279	62	married	3,000,000-4,000,000 JPY	yes	1	3	2	5	5	5	1	4	3	4	2	1	2	2	1	1	3	3	1	3	2	1	2	2	2
1280	55	married	6,000,000-7,000,000 JPY	yes	3	3	3	2	4	3	1	3	2	2	2	3	3	3	3	2	3	2	2	2	2	2	2	2	2
1281	59	married	2,000,000-3,000,000 JPY	yes	0	0	0	0	0	0	2	1	1	1	1	1	1	1	1	1	1	1	1	1	1	1	1	1	1
1282	72	married	2,000,000-3,000,000 JPY	yes	1	3	2	1	2	1	0	5	2	3	1	4	3	2	1	1	1	2	1	4	1	3	5	5	3
1283	43	unmarried	3,000,000-4,000,000 JPY	no	1	3	0	1	0	1	2	3	2	3	2	3	2	3	3	2	2	2	2	2	2	2	2	3	3
1284	55	married	2,000,000-3,000,000 JPY	yes	1	1	1	0	1	1	1	3	2	2	1	1	1	2	2	2	1	2	1	2	1	1	2	3	3
1285	51	unmarried	6,000,000-7,000,000 JPY	no	0	0	0	0	0	0	0	0	1	1	1	1	1	1	1	1	1	1	1	1	1	1	1	1	1
1286	104	unmarried	less than 1,000,000 JPY	no	0	1	0	0	0	0	5	1	1	1	1	2	2	1	1	1	3	3	2	3	1	1	3	1	2
1287	70	married	12,000,000-15,000,000 JPY	yes	1	1	1	1	1	1	1	2	2	2	1	2	2	2	1	2	2	2	2	3	1	2	3	3	2
1288	50	married	less than 1,000,000 JPY	no	2	2	3	0	0	2	1	3	2	2	5	3	2	4	2	5	5	1	2	5	1	1	2	3	2
1289	43	married	6,000,000-7,000,000 JPY	yes	0	0	0	0	0	0	3	4	1	1	1	3	3	2	1	1	1	1	1	5	1	1	4	1	3
1290	50	married	6,000,000-7,000,000 JPY	yes	1	1	0	5	1	0	1	5	3	3	1	2	2	1	1	1	2	1	1	3	1	1	2	3	2
1291	55	married	7,000,000-8,000,000 JPY	yes	5	5	4	3	5	5	3	3	1	1	1	2	2	2	1	1	2	2	1	3	1	1	2	2	2
1292	73	married	2,000,000-3,000,000 JPY	yes	1	1	3	0	1	0	1	2	1	2	1	3	2	1	1	1	1	2	1	3	1	2	5	5	5
1293	48	unmarried	1,000,000-2,000,000 JPY	no	0	0	0	0	0	0	2	0	1	1	1	1	1	1	1	1	1	1	1	1	1	1	1	1	1
1294	70	married	4,000,000-5,000,000 JPY	yes	3	2	4	1	5	4	2	4	3	4	1	2	2	1	1	1	3	3	1	4	3	1	4	5	4
1295	53	married	4,000,000-5,000,000 JPY	yes	0	0	0	0	0	0	1	3	1	1	1	1	1	1	1	1	1	1	1	1	1	1	1	1	1
1296	80	married	4,000,000-5,000,000 JPY	no	0	0	0	0	0	0	3	1	1	1	1	1	1	1	1	1	1	1	1	1	1	1	1	1	1
1297	64	married	less than 1,000,000 JPY	no	0	0	0	0	0	0	0	1	1	1	1	1	1	1	1	1	1	1	1	1	1	1	1	1	1
1298	74	married	2,000,000-3,000,000 JPY	yes	0	1	1	1	1	0	1	3	1	2	1	2	2	1	1	1	1	3	1	3	2	1	2	2	1
1299	71	married	4,000,000-5,000,000 JPY	yes	3	2	3	2	2	2	1	5	2	1	1	3	2	2	1	1	1	2	1	2	1	1	1	2	1
1300	70	married	5,000,000-6,000,000 JPY	yes	4	3	5	5	5	5	2	4	3	1	1	2	3	1	2	3	3	3	3	4	3	1	2	3	2
1301	74	married	3,000,000-4,000,000 JPY	yes	0	2	0	5	0	0	3	5	3	3	3	3	2	3	3	3	3	3	3	4	2	2	4	4	4
1302	53	unmarried	5,000,000-6,000,000 JPY	no	0	0	0	0	0	0	0	1	2	1	1	1	1	2	1	1	2	2	1	1	1	1	1	2	2
1303	74	married	6,000,000-7,000,000 JPY	yes	0	1	0	1	0	0	1	3	2	2	1	2	1	1	1	1	1	2	1	3	1	1	3	5	3
1304	66	married	4,000,000-5,000,000 JPY	yes	0	1	0	0	0	0	1	3	1	2	1	1	1	1	1	1	1	2	1	2	1	1	1	2	1
1305	54	married	6,000,000-7,000,000 JPY	no	0	0	0	0	0	0	1	1	2	3	1	2	2	1	1	1	2	3	1	2	1	3	3	2	1
1306	70	married	3,000,000-4,000,000 JPY	yes	0	1	0	0	0	0	0	1	1	1	1	1	1	2	1	1	1	1	1	1	1	1	1	1	1
1307	80	married	6,000,000-7,000,000 JPY	yes	1	1	3	4	3	2	2	3	2	2	1	2	2	1	2	1	1	2	2	3	2	1	4	5	4
1308	76	married	10,000,000-12,000,000 JPY	yes	0	1	0	0	0	0	1	1	3	1	1	3	2	3	3	2	3	2	3	3	2	1	2	2	2
1309	69	married	4,000,000-5,000,000 JPY	yes	3	2	4	5	5	5	4	6	3	3	1	1	2	2	1	2	1	1	2	5	5	2	1	5	3
1310	43	unmarried	8,000,000-9,000,000 JPY	no	1	4	0	0	0	0	0	3	1	1	2	1	1	1	1	1	1	1	1	1	1	1	1	1	1
1311	40	married	4,000,000-5,000,000 JPY	yes	1	1	0	1	0	1	0	3	3	4	1	4	2	3	3	2	2	1	2	3	1	1	1	3	1
1312	54	unmarried	5,000,000-6,000,000 JPY	yes	0	0	0	0	1	1	0	0	1	1	1	2	2	1	1	1	1	2	1	2	1	2	2	2	1
1313	59	unmarried	less than 1,000,000 JPY	no	0	0	0	0	0	0	1	2	1	1	1	1	1	1	2	1	1	1	1	1	1	1	1	1	1
1314	74	married	3,000,000-4,000,000 JPY	no	1	1	0	1	1	1	1	2	2	3	2	1	2	3	2	1	3	2	2	3	2	2	2	1	2
1315	62	married	2,000,000-3,000,000 JPY	no	0	0	0	0	0	0	0	3	1	1	1	1	1	1	1	1	1	1	1	1	1	1	2	2	2
1316	44	unmarried	3,000,000-4,000,000 JPY	no	0	0	0	0	0	0	0	1	1	1	1	2	1	2	1	1	1	1	2	2	1	1	1	2	1
1317	53	unmarried	2,000,000-3,000,000 JPY	no	1	1	0	1	0	1	0	1	1	3	1	1	1	2	2	1	2	2	1	3	1	1	2	2	2
1318	43	married	5,000,000-6,000,000 JPY	yes	3	1	1	3	1	3	1	3	3	3	3	3	3	4	3	3	2	3	3	2	3	3	2	3	4
1319	74	married	5,000,000-6,000,000 JPY	yes	0	1	1	4	5	5	2	5	3	4	1	3	3	1	1	1	4	4	2	3	3	3	3	3	4
1320	49	married	3,000,000-4,000,000 JPY	yes	0	0	0	0	0	0	0	3	1	1	1	1	1	1	1	1	1	1	1	1	1	1	1	1	1
1321	58	unmarried	3,000,000-4,000,000 JPY	no	1	4	1	2	2	0	2	5	2	3	2	2	2	3	3	2	2	2	2	1	2	2	2	2	2
1322	57	married	7,000,000-8,000,000 JPY	yes	2	1	0	1	1	0	0	3	2	2	1	4	3	1	1	1	2	2	2	2	1	1	5	4	4
1323	67	married	2,000,000-3,000,000 JPY	yes	0	1	0	0	0	0	3	5	2	1	1	1	1	1	1	1	1	1	1	1	1	1	3	3	3
1324	70	married	2,000,000-3,000,000 JPY	yes	1	1	0	1	0	0	2	3	1	1	1	2	2	1	1	1	1	2	1	1	1	1	3	3	3
1325	77	married	4,000,000-5,000,000 JPY	yes	3	1	1	3	5	1	3	4	3	3	2	3	1	1	2	2	3	3	3	4	3	2	4	5	5
1326	42	unmarried	less than 1,000,000 JPY	no	0	0	0	0	0	0	0	3	1	1	1	1	1	1	1	1	1	1	1	1	1	1	1	1	1
1327	66	married	3,000,000-4,000,000 JPY	yes	1	1	0	0	1	0	0	3	1	1	1	1	1	1	1	1	1	1	1	1	1	1	3	3	3
1328	73	married	6,000,000-7,000,000 JPY	yes	1	2	0	2	3	0	2	3	1	1	1	1	1	1	1	1	1	1	1	2	1	1	1	2	2
1329	57	unmarried	1,000,000-2,000,000 JPY	no	1	1	0	0	1	0	0	2	1	2	1	2	2	1	2	1	1	2	2	2	2	1	1	2	2
1330	51	unmarried	2,000,000-3,000,000 JPY	no	2	5	0	5	5	0	2	3	5	4	2	5	5	5	5	5	5	5	5	5	5	1	5	5	5
1331	40	married	15,000,000-18,000,000 JPY	yes	5	1	0	1	1	3	1	5	1	1	1	3	3	1	1	1	1	1	1	3	1	1	2	3	2
1332	47	married	9,000,000-10,000,000 JPY	no	1	0	0	0	0	0	2	2	1	3	4	3	3	2	1	1	2	2	1	2	1	2	2	2	2
1333	73	married	3,000,000-4,000,000 JPY	yes	2	0	0	1	2	0	2	6	3	3	2	3	2	2	1	1	2	4	1	1	1	2	4	5	4

1334	72	married	15,000,000-18,000,000 JPY	yes	0	0	0	0	0	0	5	3	1	1	1	2	2	1	1	1	1	1	1	3	1	1	3	1	3
1335	76	married	3,000,000-4,000,000 JPY	yes	1	2	2	2	2	1	3	4	2	1	1	2	2	1	1	1	2	2	1	2	1	1	2	2	3
1336	71	married	2,000,000-3,000,000 JPY	yes	3	1	1	0	1	3	0	2	1	1	1	1	1	1	1	1	1	2	1	3	1	1	3	3	2
1337	52	married	12,000,000-15,000,000 JPY	yes	1	0	0	0	0	0	5	2	1	2	1	1	3	1	1	1	1	2	1	2	1	1	1	1	1
1338	70	married	4,000,000-5,000,000 JPY	yes	5	5	4	4	5	5	0	5	3	3	2	2	3	2	2	2	3	4	3	4	2	3	4	5	2
1339	72	married	less than 1,000,000 JPY	yes	5	5	5	5	5	5	2	6	3	3	2	3	3	3	3	3	4	4	3	4	4	3	4	4	4
1340	41	unmarried	less than 1,000,000 JPY	no	4	2	3	2	2	2	1	5	2	3	3	3	2	3	4	4	4	3	3	3	3	3	4	4	4
1341	78	married	7,000,000-8,000,000 JPY	yes	0	0	0	0	0	0	1	1	1	1	1	1	1	1	1	1	1	2	1	2	1	1	3	5	3
1342	63	married	8,000,000-9,000,000 JPY	yes	3	3	5	3	5	2	1	6	2	1	1	2	3	1	1	1	2	3	1	4	1	2	4	5	1
1343	76	married	8,000,000-9,000,000 JPY	yes	1	1	0	0	1	0	2	5	3	1	2	2	3	1	1	1	2	3	3	3	3	1	4	4	4
1344	53	married	4,000,000-5,000,000 JPY	no	1	3	0	0	0	0	2	3	2	1	1	1	2	2	1	3	2	2	2	2	2	2	2	2	2
1345	58	unmarried	4,000,000-5,000,000 JPY	no	3	2	2	0	0	0	2	2	4	2	1	2	2	1	1	2	2	2	1	1	1	2	1	2	2
1346	40	married	6,000,000-7,000,000 JPY	yes	1	1	1	0	0	2	1	1	3	3	4	3	3	3	3	3	4	3	3	4	3	4	3	3	3
1347	54	married	12,000,000-15,000,000 JPY	yes	5	5	5	5	5	5	1	0	1	1	1	1	1	1	1	1	1	1	1	1	1	1	1	1	1
1348	45	married	4,000,000-5,000,000 JPY	yes	0	0	0	0	0	0	0	0	1	1	1	1	1	1	1	1	1	1	1	1	1	1	1	1	1
1349	62	married	9,000,000-10,000,000 JPY	yes	1	1	1	0	1	1	1	4	2	3	2	5	5	1	1	1	3	3	2	3	3	3	4	4	3
1350	48	married	12,000,000-15,000,000 JPY	no	0	0	0	0	0	0	0	0	3	1	1	2	2	2	1	1	2	3	1	3	1	1	1	2	1
1351	65	married	2,000,000-3,000,000 JPY	yes	5	5	5	5	5	5	0	1	1	1	1	1	1	1	1	1	1	1	1	1	1	1	1	1	1
1352	46	married	5,000,000-6,000,000 JPY	yes	1	5	0	5	0	0	2	5	3	4	2	4	4	3	3	1	5	4	2	5	2	1	2	2	2
1353	71	married	3,000,000-4,000,000 JPY	yes	0	0	0	0	0	0	4	2	2	2	1	1	2	1	1	1	1	2	1	1	2	1	2	1	2
1354	58	unmarried	3,000,000-4,000,000 JPY	no	3	2	0	0	5	2	0	3	1	1	1	1	1	1	1	1	1	2	1	2	1	1	2	1	1
1355	59	married	4,000,000-5,000,000 JPY	yes	0	1	0	0	0	0	0	3	1	2	1	2	2	2	2	2	2	2	1	1	1	1	1	3	2
1356	61	married	3,000,000-4,000,000 JPY	yes	3	2	1	1	1	1	1	4	2	2	2	2	2	1	1	1	2	2	1	2	2	2	3	3	2
1357	82	married	6,000,000-7,000,000 JPY	yes	1	1	1	0	1	1	0	3	2	2	1	1	1	1	1	1	1	2	1	2	1	1	3	3	3
1358	67	married	4,000,000-5,000,000 JPY	no	2	1	5	0	5	2	1	4	2	2	2	1	2	2	3	1	3	4	1	4	2	1	4	3	2
1359	46	married	9,000,000-10,000,000 JPY	yes	0	2	0	0	0	0	1	2	3	2	3	2	2	3	4	2	2	1	1	3	3	1	2	2	2
1360	53	married	5,000,000-6,000,000 JPY	yes	0	0	0	0	0	0	0	0	1	2	1	2	3	2	2	1	2	2	1	4	1	1	4	2	2
1361	55	married	9,000,000-10,000,000 JPY	yes	4	1	5	0	5	5	0	3	2	1	1	2	2	2	2	2	2	2	2	3	1	1	2	2	2
1362	71	married	3,000,000-4,000,000 JPY	yes	0	0	0	0	0	0	1	3	1	1	1	1	1	1	1	1	1	1	1	1	1	1	2	2	2
1363	40	unmarried	2,000,000-3,000,000 JPY	no	0	0	0	0	0	0	0	0	1	1	1	1	1	1	1	1	1	1	1	1	1	1	1	1	1
1364	64	married	3,000,000-4,000,000 JPY	yes	5	3	4	1	3	0	2	3	1	1	1	1	1	1	1	1	1	1	1	2	1	1	3	3	3
1365	71	married	12,000,000-15,000,000 JPY	yes	1	1	0	0	0	0	1	2	2	1	1	2	2	1	1	1	1	1	1	1	1	1	1	1	1
1366	43	unmarried	1,000,000-2,000,000 JPY	yes	1	1	1	1	0	0	1	1	1	2	3	2	2	2	3	1	2	2	1	2	2	1	2	2	2
1367	66	unmarried	2,000,000-3,000,000 JPY	no	1	1	0	0	1	0	3	2	1	1	1	1	1	1	1	1	1	1	1	2	1	1	2	3	1
1368	43	married	5,000,000-6,000,000 JPY	yes	2	4	3	3	2	2	3	2	3	3	3	3	3	2	2	2	2	2	2	2	2	2	2	2	2
1369	52	married	3,000,000-4,000,000 JPY	no	0	0	0	0	0	0	2	3	1	3	1	2	1	1	2	2	1	3	2	2	1	1	1	2	2
1370	51	married	1,000,000-2,000,000 JPY	no	0	1	0	0	0	0	0	2	2	1	1	3	2	2	1	1	2	2	2	3	1	1	2	3	3
1371	67	married	less than 1,000,000 JPY	yes	0	0	0	0	0	0	0	1	3	3	3	3	3	3	3	3	3	3	3	3	3	3	3	3	3
1372	55	married	12,000,000-15,000,000 JPY	no	1	3	0	1	1	0	1	3	2	3	1	2	2	2	1	1	2	2	1	3	1	2	2	2	2
1373	54	unmarried	1,000,000-2,000,000 JPY	no	0	0	0	0	0	0	4	1	3	1	1	1	2	2	1	1	1	1	4	1	1	1	1	1	1
1374	54	unmarried	less than 1,000,000 JPY	no	0	0	0	0	0	0	1	1	1	2	2	1	1	1	1	1	1	2	1	2	2	1	1	1	1
1375	54	unmarried	4,000,000-5,000,000 JPY	no	1	3	0	0	1	1	1	1	1	1	1	1	1	1	1	1	1	1	1	1	1	1	1	1	1
1376	76	married	18,000,000-20,000,000 JPY	yes	1	1	0	0	1	0	2	4	1	3	1	1	1	1	1	1	1	2	1	2	2	2	3	3	3
1377	71	married	2,000,000-3,000,000 JPY	yes	1	5	4	5	5	0	0	5	3	2	4	1	2	5	1	1	3	4	1	5	1	1	3	3	2
1378	62	married	5,000,000-6,000,000 JPY	no	0	1	0	1	1	0	2	5	2	1	1	1	1	2	1	1	1	1	1	3	1	1	4	4	4
1379	60	married	8,000,000-9,000,000 JPY	yes	1	1	0	1	0	1	3	3	2	3	1	2	3	3	3	1	3	2	2	3	2	3	2	3	1
1380	74	married	4,000,000-5,000,000 JPY	yes	3	3	3	3	3	0	2	5	2	1	1	1	3	2	1	1	1	2	1	2	1	1	4	4	4
1381	70	married	3,000,000-4,000,000 JPY	yes	2	2	2	3	4	4	1	4	2	2	1	3	2	1	1	1	1	2	1	3	1	1	4	4	3
1382	71	married	5,000,000-6,000,000 JPY	yes	5	4	0	1	1	1	1	5	2	2	2	3	2	1	2	1	2	2	2	2	1	1	4	4	3
1383	70	married	3,000,000-4,000,000 JPY	yes	1	2	1	1	5	3	1	4	2	3	4	4	3	2	2	2	3	4	3	4	3	2	2	3	3
1384	61	married	5,000,000-6,000,000 JPY	yes	0	0	0	0	0	0	0	1	1	1	1	1	1	1	1	1	1	1	1	1	1	1	1	1	1
1385	54	married	5,000,000-6,000,000 JPY	yes	0	1	0	1	1	0	2	3	1	3	1	2	3	2	2	1	2	2	2	3	2	2	2	2	2
1386	44	married	9,000,000-10,000,000 JPY	no	1	0	2	0	2	0	1	3	1	1	1	3	3	3	4	1	1	1	1	2	1	1	4	5	5
1387	49	married	4,000,000-5,000,000 JPY	yes	0	0	0	0	0	0	0	6	1	1	1	1	1	1	1	1	1	1	1	1	1	1	1	1	1
1388	70	married	3,000,000-4,000,000 JPY	yes	0	1	0	0	0	0	2	2	3	4	2	4	2	1	2	2	2	4	3	3	2	1	4	4	4
1389	70	married	2,000,000-3,000,000 JPY	yes	0	0	1	0	3	0	1	1	3	2	2	3	3	2	2	2	2	2	2	4	2	3	4	4	4
1390	72	married	2,000,000-3,000,000 JPY	yes	0	0	0	0	1	0	2	5	3	1	1	1	1	1	1	1	1	3	1	3	1	1	3	3	4
1391	74	married	10,000,000-12,000,000 JPY	yes	0	0	0	0	0	0	1	1	1	1	1	1	2	1	1	1	2	2	1	2	2	1	3	1	3

1392	83	married	1,000,000-2,000,000 JPY	yes	0	4	0	0	0	0	3	3	1	3	1	2	1	2	2	2	2	2	2	3	1	1	3	1	2
1393	51	married	18,000,000-20,000,000 JPY	yes	5	2	0	2	5	5	1	2	2	2	2	2	2	1	1	1	1	2	1	2	1	1	1	1	1
1394	70	married	2,000,000-3,000,000 JPY	yes	1	1	3	1	1	0	1	4	2	2	1	2	2	2	2	2	2	2	2	3	1	2	4	4	4
1395	59	unmarried	less than 1,000,000 JPY	no	0	0	0	0	0	0	0	3	3	1	1	2	2	2	2	1	2	2	1	2	1	1	2	2	2
1396	60	unmarried	5,000,000-6,000,000 JPY	no	0	1	0	0	1	0	1	3	2	3	1	2	3	2	2	1	3	3	2	4	2	2	2	3	3
1397	49	unmarried	10,000,000-12,000,000 JPY	no	2	1	0	1	1	1	0	4	2	2	1	3	3	2	3	2	3	2	2	4	3	2	2	3	3
1398	72	unmarried	2,000,000-3,000,000 JPY	no	0	1	1	0	1	1	1	3	2	3	2	2	2	2	2	1	2	2	2	3	2	1	2	2	2
1399	47	unmarried	3,000,000-4,000,000 JPY	no	0	1	5	5	5	5	3	5	3	1	2	2	2	1	1	1	1	1	1	1	1	1	2	1	2
1400	59	married	10,000,000-12,000,000 JPY	yes	0	0	0	0	0	0	1	2	1	1	1	1	1	2	2	1	1	2	1	2	1	1	1	1	1
1401	55	married	4,000,000-5,000,000 JPY	yes	3	2	2	2	2	1	2	4	3	2	2	3	3	3	3	2	3	3	3	3	2	2	3	3	3
1402	85	married	9,000,000-10,000,000 JPY	yes	0	0	0	0	0	0	1	2	1	1	1	1	1	1	1	1	1	2	1	2	1	1	2	3	4
1403	46	unmarried	less than 1,000,000 JPY	no	0	0	0	0	0	0	0	3	2	2	2	2	3	2	2	2	2	2	2	3	2	1	2	2	2
1404	74	married	5,000,000-6,000,000 JPY	yes	3	3	3	4	3	3	2	3	4	5	1	3	3	2	2	2	3	4	2	1	3	3	1	1	3
1405	50	married	5,000,000-6,000,000 JPY	yes	5	1	0	2	3	0	0	5	1	1	1	3	3	2	1	2	1	2	1	4	1	1	4	3	3
1406	71	unmarried	1,000,000-2,000,000 JPY	no	5	5	5	1	1	0	3	5	3	2	1	3	3	3	2	2	1	2	2	3	1	1	3	2	2
1407	42	unmarried	3,000,000-4,000,000 JPY	no	4	4	4	4	4	4	2	3	3	2	2	2	2	2	2	2	2	1	2	2	2	2	3	3	3
1408	58	married	12,000,000-15,000,000 JPY	yes	1	0	0	0	1	0	1	1	1	2	2	2	1	1	1	1	2	2	2	2	2	1	2	2	2
1409	56	married	20,000,000 or more JPY	no	1	1	0	0	0	0	1	2	1	1	1	3	2	2	1	1	2	1	1	2	1	1	1	1	2
1410	70	married	4,000,000-5,000,000 JPY	yes	3	5	3	3	5	3	4	3	2	2	1	2	2	2	2	3	3	3	2	3	3	2	2	2	2
1411	79	married	4,000,000-5,000,000 JPY	yes	0	1	2	2	4	0	3	3	1	1	1	1	1	1	1	1	1	2	1	2	1	1	3	3	2
1412	51	unmarried	less than 1,000,000 JPY	no	0	0	0	0	0	0	0	0	1	2	1	1	1	1	1	1	1	1	1	5	1	1	1	1	1
1413	52	married	9,000,000-10,000,000 JPY	no	0	0	0	0	0	0	1	3	1	1	1	1	1	1	1	1	1	1	1	1	1	1	1	1	1
1414	44	unmarried	5,000,000-6,000,000 JPY	no	0	1	0	1	0	0	0	1	3	3	2	3	3	3	2	2	4	2	4	5	3	1	4	5	5
1415	63	married	2,000,000-3,000,000 JPY	no	0	3	0	1	0	0	1	5	3	3	1	1	2	2	3	2	2	3	3	4	1	1	5	5	5
1416	63	married	3,000,000-4,000,000 JPY	yes	5	5	5	0	5	0	1	6	4	3	1	2	2	1	2	1	1	4	2	3	3	1	5	5	5
1417	68	married	less than 1,000,000 JPY	yes	0	0	0	0	0	0	0	3	1	1	1	1	1	1	1	1	1	1	1	2	1	1	2	2	2
1418	52	unmarried	9,000,000-10,000,000 JPY	no	0	0	0	0	0	0	1	4	1	1	1	2	2	1	2	1	1	2	1	1	2	1	1	1	1
1419	84	married	12,000,000-15,000,000 JPY	yes	2	2	1	0	1	1	1	2	1	2	1	2	1	1	1	1	1	2	1	2	1	1	2	2	2
1420	72	married	1,000,000-2,000,000 JPY	no	1	1	0	0	1	0	1	2	2	1	3	2	2	1	1	2	2	2	1	2	1	2	2	2	1
1421	56	married	7,000,000-8,000,000 JPY	yes	0	0	0	0	0	0	1	1	1	1	1	2	2	1	2	1	1	2	1	1	1	1	2	2	2
1422	64	married	less than 1,000,000 JPY	yes	1	1	2	2	5	4	1	4	2	3	3	4	3	4	3	3	4	4	4	4	4	3	4	4	4
1423	64	married	2,000,000-3,000,000 JPY	yes	0	0	0	0	0	0	5	3	3	4	3	4	4	2	2	2	2	3	2	2	3	3	3	3	3
1424	58	married	2,000,000-3,000,000 JPY	no	0	1	0	0	1	0	1	2	1	2	1	1	1	1	1	1	1	2	1	1	1	3	3	1	1
1425	44	married	7,000,000-8,000,000 JPY	no	1	1	0	0	1	0	0	3	3	3	2	5	3	3	4	2	3	3	4	5	3	1	4	4	3
1426	48	married	9,000,000-10,000,000 JPY	yes	3	3	3	4	2	3	1	3	3	3	3	3	3	3	3	3	3	3	3	3	3	4	3	3	3
1427	71	married	4,000,000-5,000,000 JPY	yes	0	1	2	1	1	0	1	2	1	2	1	1	1	1	1	2	1	2	1	3	1	2	2	1	1
1428	73	married	5,000,000-6,000,000 JPY	yes	0	1	0	0	0	0	1	3	2	1	1	1	1	1	1	1	1	2	1	2	1	1	1	1	2
1429	55	unmarried	4,000,000-5,000,000 JPY	no	0	0	0	0	0	0	1	3	1	1	1	1	1	1	1	1	1	2	1	1	1	1	1	1	1
1430	40	married	6,000,000-7,000,000 JPY	no	0	0	0	0	0	0	0	0	1	1	1	1	1	1	1	1	1	1	1	1	1	1	1	1	1
1431	62	married	3,000,000-4,000,000 JPY	yes	0	1	0	3	0	0	1	3	1	1	1	1	2	1	1	1	2	2	1	3	1	1	2	3	3
1432	59	married	9,000,000-10,000,000 JPY	no	1	0	1	0	2	0	5	3	1	1	2	1	2	2	1	1	2	2	1	2	1	1	2	2	1
1433	42	married	6,000,000-7,000,000 JPY	yes	1	1	0	0	0	0	0	1	1	1	2	2	3	3	2	1	2	2	2	2	1	1	2	3	2
1434	58	unmarried	6,000,000-7,000,000 JPY	no	1	1	0	1	1	1	1	4	1	2	1	1	1	1	1	1	1	2	1	2	1	1	2	1	2
1435	60	unmarried	less than 1,000,000 JPY	no	1	1	3	1	1	0	3	6	1	1	1	4	4	3	2	3	4	4	3	4	3	1	3	4	3
1436	52	married	8,000,000-9,000,000 JPY	yes	1	2	1	0	0	0	0	4	1	1	2	1	1	1	1	1	1	1	1	1	1	1	2	1	2
1437	47	unmarried	8,000,000-9,000,000 JPY	no	5	3	2	1	2	2	3	3	3	3	3	2	2	5	2	3	3	3	2	4	2	2	3	3	3
1438	55	married	3,000,000-4,000,000 JPY	no	0	0	0	0	0	0	3	1	1	2	1	2	2	2	1	1	2	2	1	2	1	1	1	1	2
1439	63	married	10,000,000-12,000,000 JPY	yes	1	2	2	2	2	5	3	3	3	3	3	4	2	1	1	1	2	3	1	3	1	1	4	5	2
1440	78	married	2,000,000-3,000,000 JPY	yes	4	3	3	4	3	3	2	4	3	4	2	2	1	3	2	1	3	4	4	5	3	2	2	1	2
1441	76	married	2,000,000-3,000,000 JPY	yes	2	4	5	4	5	4	3	3	2	1	3	3	2	2	2	1	2	2	4	4	1	5	5	3	2
1442	73	married	3,000,000-4,000,000 JPY	yes	1	0	0	0	2	2	0	1	2	1	2	1	1	1	1	1	1	2	1	2	2	2	4	4	2
1443	62	married	3,000,000-4,000,000 JPY	yes	0	0	0	0	0	0	1	3	1	3	1	1	2	1	1	1	2	2	1	2	1	1	1	4	2
1444	51	unmarried	1,000,000-2,000,000 JPY	no	0	0	0	0	0	0	0	1	1	1	2	1	1	3	3	2	1	1	2	4	2	1	2	2	2
1445	45	married	10,000,000-12,000,000 JPY	no	0	0	0	0	0	0	0	1	1	1	1	1	2	1	1	1	1	1	1	1	1	1	1	1	1
1446	71	married	3,000,000-4,000,000 JPY	yes	3	4	2	1	1	2	5	5	1	1	1	1	2	2	1	1	1	2	1	4	1	1	4	4	4
1447	72	married	2,000,000-3,000,000 JPY	yes	0	1	1	2	2	0	2	2	1	3	1	1	1	2	2	1	1	2	2	3	2	2	3	2	2
1448	42	married	7,000,000-8,000,000 JPY	yes	1	1	1	0	1	1	1	3	1	1	1	1	1	1	1	1	1	1	1	1	1	1	1	1	1
1449	52	married	9,000,000-10,000,000 JPY	yes	0	1	0	1	0	0	2	3	2	3	1	2	3	2	4	3	2	2	3	3	2	2	3	3	2

1450	48	married	7,000,000-8,000,000 JPY	yes	0	1	0	0	0	0	1	2	2	2	1	1	1	1	1	1	1	1	1	2	1	1	1	1	1
1451	81	married	4,000,000-5,000,000 JPY	yes	1	3	3	3	3	3	2	3	3	4	2	3	3	2	2	2	2	4	4	4	3	3	4	4	4
1452	46	married	6,000,000-7,000,000 JPY	yes	3	3	1	2	3	3	0	4	3	2	2	2	3	2	3	3	3	3	2	3	5	3	3	2	2
1453	79	married	2,000,000-3,000,000 JPY	yes	2	2	4	4	4	2	2	4	2	4	2	5	3	2	2	1	2	3	1	2	3	3	3	3	3
1454	40	married	6,000,000-7,000,000 JPY	yes	0	0	0	0	0	0	1	6	4	4	4	3	3	4	3	1	3	4	1	1	3	1	3	3	3
1455	57	unmarried	less than 1,000,000 JPY	no	0	0	0	0	0	0	0	3	1	1	1	1	1	1	1	1	1	1	1	1	1	1	1	1	1
1456	57	married	15,000,000-18,000,000 JPY	yes	0	5	0	1	0	0	1	3	4	3	2	3	3	3	3	3	4	4	3	5	3	3	4	4	4
1457	74	married	2,000,000-3,000,000 JPY	yes	0	0	0	0	1	0	1	4	2	1	1	1	2	1	1	1	1	3	1	4	2	1	3	4	4
1458	48	married	20,000,000 or more JPY	yes	1	1	0	0	1	1	1	3	1	1	1	1	1	1	1	1	1	2	1	1	1	1	2	2	2
1459	59	unmarried	1,000,000-2,000,000 JPY	no	1	5	1	1	4	0	2	5	3	3	1	3	3	3	3	1	3	3	2	3	2	1	2	2	2
1460	71	married	3,000,000-4,000,000 JPY	no	0	1	0	1	1	1	3	3	1	1	1	1	1	1	1	1	1	2	1	2	1	1	3	4	5
1461	53	unmarried	3,000,000-4,000,000 JPY	no	1	1	1	1	1	0	0	3	2	3	3	2	2	1	1	1	1	1	2	2	1	1	2	3	3
1462	59	married	5,000,000-6,000,000 JPY	yes	0	2	0	0	2	0	1	3	1	1	1	2	1	1	1	1	1	1	1	3	1	2	2	2	2
1463	47	married	7,000,000-8,000,000 JPY	yes	5	5	0	2	0	0	2	6	2	2	1	4	2	5	5	2	2	2	4	5	1	1	3	1	1
1464	71	married	8,000,000-9,000,000 JPY	yes	0	3	1	1	2	0	2	5	1	1	1	1	1	1	1	1	1	1	1	1	1	1	1	3	2
1465	52	married	8,000,000-9,000,000 JPY	yes	1	1	1	0	1	1	0	1	1	1	2	3	2	1	1	1	2	3	1	2	1	1	2	1	2
1466	56	unmarried	less than 1,000,000 JPY	no	0	0	0	0	0	0	0	2	1	1	1	1	1	1	1	1	1	1	1	1	1	1	1	1	1
1467	65	married	5,000,000-6,000,000 JPY	yes	1	1	0	1	1	0	1	2	1	4	1	3	2	1	2	1	2	3	2	4	3	2	4	4	2
1468	71	married	2,000,000-3,000,000 JPY	yes	4	1	0	1	0	0	3	5	3	2	1	1	1	1	1	1	1	3	2	3	1	1	2	2	2
1469	83	married	3,000,000-4,000,000 JPY	yes	0	0	0	0	0	1	1	3	3	3	1	1	1	1	2	1	2	3	1	4	1	1	4	5	5
1470	47	married	4,000,000-5,000,000 JPY	yes	0	0	0	1	0	0	2	1	1	2	1	1	1	2	2	1	1	2	1	2	1	1	1	1	1
1471	58	married	15,000,000-18,000,000 JPY	yes	5	5	5	0	0	0	2	5	1	2	2	2	1	2	2	1	2	2	1	5	1	1	2	3	2
1472	48	married	5,000,000-6,000,000 JPY	yes	0	0	0	0	0	0	0	1	1	2	1	1	1	2	1	1	2	2	1	2	1	1	1	1	2
1473	49	unmarried	less than 1,000,000 JPY	no	4	3	2	0	0	0	2	5	5	2	1	3	4	2	1	1	3	3	4	4	2	1	1	3	1
1474	106	unmarried	20,000,000 or more JPY	no	0	0	0	0	0	0	0	0	1	1	1	1	1	1	1	1	1	1	1	1	1	1	1	1	1
1475	54	unmarried	8,000,000-9,000,000 JPY	no	1	5	2	4	4	2	2	6	3	2	1	3	3	1	2	2	4	3	2	4	3	1	3	3	3
1476	41	unmarried	9,000,000-10,000,000 JPY	no	0	0	0	0	0	0	0	0	1	1	1	1	1	1	1	1	1	1	1	1	1	1	1	1	1
1477	58	married	4,000,000-5,000,000 JPY	yes	0	2	0	2	2	0	1	5	5	5	2	2	2	2	2	2	4	5	4	4	4	2	5	5	5
1478	69	married	9,000,000-10,000,000 JPY	yes	0	1	5	2	4	5	0	3	1	2	1	1	2	1	1	1	1	1	1	1	1	2	2	2	1
1479	48	married	4,000,000-5,000,000 JPY	yes	0	0	0	0	0	0	1	0	1	1	1	1	1	1	1	1	1	1	1	1	1	1	1	1	1
1480	49	married	5,000,000-6,000,000 JPY	yes	3	0	0	0	0	0	2	3	1	1	1	1	1	1	1	1	1	2	1	1	1	1	1	1	2
1481	67	unmarried	1,000,000-2,000,000 JPY	no	1	0	0	0	0	0	0	3	1	3	1	1	2	1	1	1	2	3	1	2	1	1	1	2	1
1482	45	married	10,000,000-12,000,000 JPY	no	0	0	0	0	0	0	0	1	1	1	1	1	1	1	1	1	1	1	1	1	1	1	1	1	1
1483	60	married	10,000,000-12,000,000 JPY	yes	0	0	0	0	0	0	0	1	1	1	1	1	1	1	1	1	1	1	1	3	1	1	1	1	1
1484	41	married	6,000,000-7,000,000 JPY	yes	0	0	0	0	0	0	0	1	1	1	1	1	1	1	1	1	1	1	1	1	1	1	1	1	1
1485	49	unmarried	less than 1,000,000 JPY	no	0	0	0	0	0	0	1	3	1	1	1	1	1	1	1	1	1	1	1	1	1	1	1	1	1
1486	56	married	10,000,000-12,000,000 JPY	yes	1	0	2	0	5	0	1	3	3	2	2	2	1	1	1	1	2	3	1	4	2	1	2	2	1
1487	44	unmarried	1,000,000-2,000,000 JPY	no	2	3	1	1	1	0	1	3	1	1	3	2	2	3	4	2	2	2	3	4	3	1	3	3	2
1488	49	unmarried	2,000,000-3,000,000 JPY	no	0	0	0	0	0	0	0	3	1	1	1	1	1	1	1	1	1	1	1	1	1	1	1	1	1
1489	71	married	5,000,000-6,000,000 JPY	yes	0	1	0	1	0	0	2	4	1	2	1	1	1	1	1	1	1	2	1	2	1	1	1	1	1
1490	45	married	6,000,000-7,000,000 JPY	yes	1	1	0	2	0	0	1	3	1	2	1	2	3	3	3	1	2	1	2	3	1	1	2	1	1
1491	79	married	3,000,000-4,000,000 JPY	yes	3	5	5	5	5	4	2	6	3	3	3	3	3	3	3	3	3	3	3	3	3	3	3	3	3
1492	43	married	6,000,000-7,000,000 JPY	yes	1	1	3	4	0	1	0	3	1	1	1	1	2	4	3	1	3	2	1	2	1	1	1	2	1
1493	52	married	20,000,000 or more JPY	yes	0	1	0	0	1	0	1	4	1	1	1	1	1	1	1	1	1	2	1	4	1	1	3	3	3
1494	71	married	4,000,000-5,000,000 JPY	yes	0	1	0	0	0	0	1	2	2	4	1	1	2	2	1	1	1	3	1	1	1	1	1	3	1
1495	72	married	1,000,000-2,000,000 JPY	yes	1	1	2	1	1	2	2	4	2	3	1	4	3	2	2	2	2	3	3	4	2	2	5	5	4
1496	51	married	4,000,000-5,000,000 JPY	yes	5	2	0	3	5	0	1	5	3	5	4	4	4	2	4	4	4	2	3	3	2	1	2	2	2
1497	48	married	4,000,000-5,000,000 JPY	yes	2	1	0	1	0	0	1	3	2	2	1	1	2	2	1	1	3	3	1	2	1	5	1	1	1
1498	43	married	3,000,000-4,000,000 JPY	yes	1	1	1	1	0	1	2	4	3	4	2	3	3	3	3	3	4	3	4	3	2	1	2	1	1
1499	43	married	6,000,000-7,000,000 JPY	yes	0	0	0	0	0	0	0	0	1	1	1	1	1	2	1	1	2	2	1	2	1	1	1	1	1
1500	61	married	6,000,000-7,000,000 JPY	yes	4	2	2	4	4	0	2	6	3	3	3	2	3	3	2	2	4	4	2	4	3	1	2	5	1
1501	46	married	4,000,000-5,000,000 JPY	yes	3	3	3	4	3	3	2	6	3	3	3	3	4	3	3	2	3	3	2	3	2	3	3	3	3
1502	45	unmarried	less than 1,000,000 JPY	no	0	0	0	0	0	0	0	3	3	3	2	2	2	2	2	2	2	2	2	1	2	2	2	2	2
1503	58	married	7,000,000-8,000,000 JPY	yes	0	1	0	0	0	0	0	1	1	1	1	1	1	1	1	1	2	1	1	2	2	1	2	2	2
1504	58	married	9,000,000-10,000,000 JPY	yes	0	3	1	0	5	0	0	1	3	2	1	1	3	1	1	1	3	3	1	3	1	1	2	3	2
1505	59	married	12,000,000-15,000,000 JPY	yes	0	2	0	0	1	0	1	4	2	3	1	1	2	1	1	1	2	2	1	3	1	1	2	2	3
1506	41	married	12,000,000-15,000,000 JPY	yes	0	0	0	0	0	0	0	1	1	1	1	1	1	1	1	1	1	1	1	2	1	1	1	1	1
1507	52	married	2,000,000-3,000,000 JPY	yes	5	4	3	3	5	5	3	5	2	3	2	4	4	2	2	2	2	3	2	3	2	2	3	3	3

1508	40	married	5,000,000-6,000,000 JPY	yes	1	0	0	0	0	1	1	3	2	2	1	2	3	2	2	1	2	2	1	2	1	1	1	1	1
1509	44	married	4,000,000-5,000,000 JPY	no	0	1	0	1	0	0	3	3	1	2	2	2	2	2	1	2	3	3	2	3	1	1	2	2	2
1510	43	unmarried	5,000,000-6,000,000 JPY	no	1	5	5	0	1	1	3	6	3	4	1	4	4	5	4	1	3	4	3	5	5	1	3	3	3
1511	44	married	6,000,000-7,000,000 JPY	yes	1	1	0	1	0	0	1	3	1	1	2	3	2	1	1	1	2	1	1	3	1	1	1	1	1
1512	43	unmarried	less than 1,000,000 JPY	yes	0	0	5	0	0	5	5	0	1	1	1	1	1	1	1	1	1	1	1	1	1	1	1	1	1
1513	46	married	4,000,000-5,000,000 JPY	no	0	0	0	0	0	0	4	3	1	1	1	1	1	1	1	1	1	1	1	1	1	1	1	1	1
1514	59	unmarried	7,000,000-8,000,000 JPY	no	5	4	1	1	2	3	3	3	3	3	3	3	3	3	3	3	3	3	3	3	3	3	3	3	3
1515	58	unmarried	5,000,000-6,000,000 JPY	yes	3	3	3	2	3	2	1	3	2	1	1	2	3	2	1	1	1	1	1	1	2	1	4	3	2
1516	56	married	8,000,000-9,000,000 JPY	yes	1	1	1	0	5	0	0	2	2	4	3	2	2	2	1	2	3	3	3	4	3	3	4	4	3
1517	50	unmarried	5,000,000-6,000,000 JPY	no	1	4	1	1	5	0	1	3	1	2	1	1	1	1	1	1	1	1	1	1	1	1	1	1	1
1518	50	married	4,000,000-5,000,000 JPY	no	1	1	1	0	1	0	3	3	2	3	2	2	3	2	2	2	1	1	1	3	2	1	2	3	1
1519	63	unmarried	less than 1,000,000 JPY	yes	5	5	5	5	5	5	1	3	2	2	2	2	2	2	2	2	2	2	2	2	2	2	2	2	2
1520	62	unmarried	less than 1,000,000 JPY	no	5	5	5	3	5	1	3	3	3	1	1	3	3	2	2	1	2	2	2	2	2	2	2	2	2
1521	69	married	6,000,000-7,000,000 JPY	yes	0	3	5	4	5	0	1	3	2	1	1	1	2	1	2	1	3	4	4	3	1	1	1	4	2
1522	53	married	4,000,000-5,000,000 JPY	yes	1	3	4	4	4	4	2	6	2	3	1	5	2	1	1	1	3	3	2	5	3	2	5	5	5
1523	52	married	10,000,000-12,000,000 JPY	yes	0	0	0	0	0	0	2	3	1	2	2	1	2	1	1	1	1	1	1	2	1	1	1	1	1
1524	40	unmarried	6,000,000-7,000,000 JPY	no	0	0	0	0	0	0	2	3	1	1	1	1	1	1	2	2	2	3	2	1	2	1	1	1	1
1525	47	married	4,000,000-5,000,000 JPY	yes	3	3	1	4	3	2	1	2	3	3	2	2	3	3	1	3	3	3	3	2	2	2	2	2	1
1526	68	married	6,000,000-7,000,000 JPY	yes	4	5	4	4	4	5	2	5	2	2	1	1	2	2	3	1	2	3	2	3	2	1	3	4	4
1527	60	married	5,000,000-6,000,000 JPY	no	0	0	0	0	0	0	0	3	2	1	1	1	2	2	2	2	1	2	2	2	1	1	2	1	1
1528	40	married	4,000,000-5,000,000 JPY	yes	0	0	0	0	0	0	1	2	1	1	1	1	2	2	2	1	1	1	1	1	1	1	1	1	1
1529	70	married	2,000,000-3,000,000 JPY	yes	5	1	3	1	5	1	1	3	2	2	2	1	2	2	1	1	3	3	2	5	2	1	4	5	5
1530	56	married	7,000,000-8,000,000 JPY	yes	1	1	0	0	1	0	1	2	1	2	1	1	2	2	1	1	2	2	1	3	1	1	2	2	2
1531	71	married	3,000,000-4,000,000 JPY	yes	0	1	0	0	0	0	0	2	1	1	1	1	1	1	2	1	1	2	1	2	1	1	2	1	1
1532	52	married	10,000,000-12,000,000 JPY	yes	0	1	0	0	0	0	1	3	2	1	1	2	1	3	2	4	2	3	3	5	2	1	3	5	5
1533	57	unmarried	4,000,000-5,000,000 JPY	no	0	0	1	0	0	0	1	2	1	2	1	2	2	2	1	1	1	2	1	1	1	1	2	3	2
1534	81	married	3,000,000-4,000,000 JPY	yes	0	0	0	0	1	0	1	2	1	2	1	1	2	1	1	1	2	3	2	4	3	2	4	4	4
1535	74	married	5,000,000-6,000,000 JPY	yes	0	0	0	0	1	0	1	1	1	2	1	1	1	1	1	1	1	1	1	2	1	1	2	2	2
1536	68	married	6,000,000-7,000,000 JPY	yes	1	1	0	1	1	0	1	4	2	2	1	1	1	1	1	1	1	2	1	2	1	2	2	2	2
1537	62	unmarried	6,000,000-7,000,000 JPY	no	0	0	0	0	0	0	1	4	1	2	1	1	2	1	1	1	2	1	1	2	1	1	1	1	1
1538	49	married	4,000,000-5,000,000 JPY	yes	0	0	1	0	5	1	0	4	3	3	2	1	2	2	1	1	1	3	1	4	2	2	5	5	4
1539	73	married	4,000,000-5,000,000 JPY	yes	1	1	1	1	1	1	0	2	2	2	1	1	1	1	1	1	1	2	1	4	1	1	2	2	2
1540	59	married	5,000,000-6,000,000 JPY	yes	1	1	0	0	1	1	1	3	2	3	1	4	2	2	2	1	2	3	2	3	1	1	2	2	2
1541	55	unmarried	5,000,000-6,000,000 JPY	no	1	1	0	1	1	0	1	4	1	2	1	2	2	1	1	1	2	2	1	3	1	1	2	2	2
1542	74	married	4,000,000-5,000,000 JPY	yes	1	2	0	4	0	0	2	3	3	2	1	1	3	1	1	1	2	3	1	5	1	1	5	5	3
1543	72	married	3,000,000-4,000,000 JPY	yes	0	1	0	1	5	0	2	5	1	3	1	1	2	1	1	1	1	3	1	3	1	1	4	4	3
1544	63	married	4,000,000-5,000,000 JPY	yes	0	0	0	0	0	0	1	3	2	2	1	1	1	1	1	1	1	1	1	3	1	1	1	1	1
1545	52	unmarried	less than 1,000,000 JPY	no	0	0	0	0	0	0	1	2	1	1	1	1	1	2	1	1	1	1	1	1	1	1	1	1	1
1546	74	married	3,000,000-4,000,000 JPY	yes	5	5	5	4	4	4	0	6	2	2	1	1	1	1	1	1	2	2	1	3	1	1	3	3	1
1547	73	married	3,000,000-4,000,000 JPY	yes	5	5	5	5	5	0	2	6	3	4	2	3	4	4	4	3	4	5	4	5	4	3	5	4	5
1548	77	married	5,000,000-6,000,000 JPY	yes	5	4	4	4	4	4	2	5	3	3	2	3	3	3	3	3	3	3	3	3	3	3	3	3	3
1549	44	unmarried	1,000,000-2,000,000 JPY	no	0	0	0	0	0	0	0	3	1	1	1	1	1	1	1	1	1	1	1	1	1	1	1	1	1
1550	68	married	3,000,000-4,000,000 JPY	yes	4	4	4	4	4	3	4	3	2	2	1	1	1	1	1	1	4	3	1	3	1	1	3	4	4
1551	52	married	8,000,000-9,000,000 JPY	yes	1	1	0	0	1	0	1	1	1	1	1	1	1	2	1	1	2	2	1	3	1	1	3	3	3
1552	75	married	4,000,000-5,000,000 JPY	no	0	1	2	0	2	0	1	3	2	2	2	1	2	1	1	1	2	2	1	3	1	1	3	3	3
1553	85	married	9,000,000-10,000,000 JPY	yes	5	5	5	5	5	5	3	4	3	3	3	3	3	3	3	3	3	3	3	3	3	3	3	3	3
1554	40	married	6,000,000-7,000,000 JPY	yes	0	0	0	0	0	0	0	0	1	1	1	1	1	1	1	1	1	1	1	1	1	1	1	1	1
1555	57	married	5,000,000-6,000,000 JPY	yes	0	0	0	0	1	0	0	1	1	1	1	1	1	1	1	1	2	3	1	3	1	2	2	1	1
1556	67	unmarried	6,000,000-7,000,000 JPY	yes	0	0	0	0	0	0	0	0	1	1	1	1	1	1	1	1	1	1	1	1	1	1	1	1	1
1557	44	married	10,000,000-12,000,000 JPY	yes	0	1	0	0	0	0	2	3	2	2	1	2	3	2	2	1	1	2	1	2	2	1	1	1	1
1558	45	married	5,000,000-6,000,000 JPY	no	1	1	0	0	1	0	4	1	1	1	1	2	2	2	2	2	2	3	3	2	1	1	2	1	2
1559	52	married	5,000,000-6,000,000 JPY	yes	2	1	0	0	2	0	2	4	3	2	2	5	4	1	4	2	3	2	4	4	3	3	4	5	2
1560	60	married	6,000,000-7,000,000 JPY	no	0	0	0	0	0	0	1	2	1	2	1	2	2	2	2	1	2	2	2	1	1	1	1	1	1
1561	84	married	1,000,000-2,000,000 JPY	yes	1	1	2	0	2	1	3	3	2	3	1	3	2	2	2	1	2	4	2	4	2	2	2	2	1
1562	49	unmarried	3,000,000-4,000,000 JPY	no	1	1	1	1	1	0	1	5	4	4	4	5	4	4	4	2	4	4	4	4	3	2	3	3	3
1563	73	married	3,000,000-4,000,000 JPY	yes	5	5	5	1	5	5	2	6	3	3	2	5	4	3	3	3	3	3	3	4	3	3	5	5	3
1564	41	married	5,000,000-6,000,000 JPY	no	0	0	0	0	0	0	0	0	1	2	1	1	2	1	1	1	1	1	1	2	1	1	2	2	2
1565	79	married	5,000,000-6,000,000 JPY	yes	2	5	1	3	1	1	5	5	2	1	1	3	3	1	1	1	1	3	1	4	2	2	4	4	4

1566	52	unmarried	6,000,000-7,000,000 JPY	no	0	1	0	0	1	0	0	4	1	1	2	1	2	1	1	1	1	1	1	1	1	1	2	2	1
1567	62	unmarried	1,000,000-2,000,000 JPY	no	0	0	1	0	2	0	1	2	2	2	2	2	2	2	3	1	3	3	3	3	3	1	2	3	3
1568	44	married	4,000,000-5,000,000 JPY	yes	0	0	0	0	0	0	5	3	1	1	1	1	1	1	1	1	1	1	1	2	1	1	1	1	1
1569	75	married	4,000,000-5,000,000 JPY	yes	1	5	5	5	1	4	1	4	2	2	1	1	1	1	1	1	1	2	2	2	2	1	2	2	2
1570	50	married	9,000,000-10,000,000 JPY	no	0	0	0	0	0	0	0	2	1	1	1	1	1	1	1	1	1	1	1	1	1	1	1	1	1
1571	73	married	10,000,000-12,000,000 JPY	no	0	0	0	0	0	0	0	0	1	1	1	1	1	1	1	1	1	1	1	1	1	1	1	1	1
1572	43	married	6,000,000-7,000,000 JPY	yes	0	0	0	0	1	0	0	0	1	1	1	1	2	2	1	1	2	2	2	3	2	1	1	2	2
1573	49	married	9,000,000-10,000,000 JPY	yes	0	0	0	0	0	0	1	3	1	1	1	1	1	1	1	1	1	1	1	1	1	1	1	1	1
1574	72	married	4,000,000-5,000,000 JPY	yes	1	5	3	1	5	4	3	5	2	1	1	3	2	1	1	1	1	2	1	3	1	1	3	2	2
1575	73	married	2,000,000-3,000,000 JPY	yes	0	0	0	0	0	0	1	3	1	1	1	1	1	1	1	1	1	1	1	1	1	1	1	1	1
1576	70	married	1,000,000-2,000,000 JPY	yes	0	1	2	2	2	1	4	3	1	2	1	2	2	1	1	1	1	1	1	3	1	1	2	4	1
1577	65	married	2,000,000-3,000,000 JPY	yes	0	1	0	1	1	0	0	2	2	3	1	2	1	2	1	2	2	2	1	3	1	1	2	3	2
1578	41	unmarried	6,000,000-7,000,000 JPY	no	0	0	0	0	0	0	0	3	1	1	1	1	1	1	1	1	1	1	1	1	1	1	1	1	1
1579	72	unmarried	3,000,000-4,000,000 JPY	no	2	2	0	2	2	1	1	2	2	2	1	1	1	1	1	1	1	2	1	2	1	1	2	1	1
1580	59	unmarried	7,000,000-8,000,000 JPY	no	1	1	1	1	1	1	4	5	3	2	2	2	2	2	3	2	2	2	2	2	2	2	2	2	2
1581	61	married	5,000,000-6,000,000 JPY	yes	0	1	0	0	0	0	1	3	2	2	2	2	3	2	2	2	3	3	2	3	2	1	3	3	3
1582	74	married	4,000,000-5,000,000 JPY	no	0	0	0	0	0	0	5	1	1	2	1	2	1	1	1	1	1	1	1	2	1	1	1	1	1
1583	70	married	4,000,000-5,000,000 JPY	yes	1	1	0	2	1	1	2	5	1	2	1	1	1	1	1	1	1	2	1	2	1	1	1	1	1
1584	50	unmarried	6,000,000-7,000,000 JPY	no	0	0	0	0	0	0	1	3	2	2	3	2	3	3	3	1	2	2	2	2	2	2	2	2	2
1585	56	unmarried	3,000,000-4,000,000 JPY	no	2	1	0	1	1	0	1	5	3	2	2	3	2	4	4	4	4	4	4	4	3	2	4	4	4
1586	70	unmarried	3,000,000-4,000,000 JPY	no	0	0	0	0	0	0	0	0	1	1	1	1	1	1	1	1	1	1	1	1	1	1	1	1	1
1587	49	unmarried	5,000,000-6,000,000 JPY	no	1	0	1	0	1	0	0	3	1	1	1	1	1	1	1	1	1	1	1	1	1	1	1	1	1
1588	72	married	2,000,000-3,000,000 JPY	yes	0	0	0	0	0	0	1	2	1	2	1	1	1	1	1	1	1	2	1	2	1	1	2	2	2
1589	73	married	5,000,000-6,000,000 JPY	yes	1	1	1	1	1	1	3	5	3	2	2	4	3	2	1	1	1	1	2	2	1	1	2	3	3
1590	54	married	less than 1,000,000 JPY	no	0	0	1	0	0	0	0	2	2	1	1	2	1	1	1	1	1	2	1	2	1	1	2	2	1
1591	65	married	2,000,000-3,000,000 JPY	no	1	0	5	1	5	5	1	4	2	2	1	3	3	2	2	1	1	3	1	3	1	1	3	3	2
1592	78	married	9,000,000-10,000,000 JPY	yes	1	1	0	0	5	0	0	4	2	2	1	1	2	1	1	1	1	2	1	3	1	1	3	3	3
1593	75	married	4,000,000-5,000,000 JPY	yes	1	2	0	2	1	0	1	4	1	1	1	1	1	1	1	1	1	2	1	2	1	1	1	1	1
1594	50	unmarried	1,000,000-2,000,000 JPY	no	4	4	3	3	3	2	2	3	3	4	4	3	3	3	3	3	4	3	4	4	4	3	3	3	3
1595	53	married	5,000,000-6,000,000 JPY	yes	0	1	0	0	0	1	1	0	2	3	3	2	3	1	1	1	2	3	1	3	2	1	3	3	3
1596	66	married	9,000,000-10,000,000 JPY	no	0	2	0	0	5	0	2	3	1	1	1	1	1	1	1	1	1	1	1	2	1	1	1	2	1
1597	56	unmarried	3,000,000-4,000,000 JPY	no	1	1	0	1	0	0	0	3	3	3	1	3	2	1	1	1	2	2	2	3	2	1	3	3	2
1598	42	unmarried	5,000,000-6,000,000 JPY	no	5	4	5	5	5	4	0	3	3	3	2	4	4	3	3	1	3	4	3	5	2	1	4	4	3
1599	54	unmarried	4,000,000-5,000,000 JPY	no	0	1	0	0	1	1	5	1	1	1	3	1	1	1	1	1	1	1	1	1	1	1	1	1	1
1600	85	married	3,000,000-4,000,000 JPY	yes	0	1	1	5	4	0	1	3	2	2	1	1	2	1	1	1	1	2	1	2	1	1	3	2	3
1601	70	married	12,000,000-15,000,000 JPY	yes	0	0	0	0	0	0	0	0	1	2	1	1	1	1	1	1	1	2	1	3	1	1	1	3	1
1602	58	married	9,000,000-10,000,000 JPY	yes	5	5	2	1	4	5	5	6	4	4	3	5	3	3	5	4	4	4	5	5	4	3	5	5	4
1603	47	unmarried	3,000,000-4,000,000 JPY	no	0	3	1	2	5	1	1	3	2	3	2	3	3	3	2	2	2	3	2	2	1	2	3	3	1
1604	44	married	10,000,000-12,000,000 JPY	yes	5	5	5	1	5	1	5	5	1	1	1	1	4	1	4	1	4	4	4	5	4	1	5	5	5
1605	71	married	2,000,000-3,000,000 JPY	yes	0	0	0	0	0	0	1	1	1	1	1	1	1	1	1	1	1	1	1	1	1	2	1	1	1
1606	66	married	7,000,000-8,000,000 JPY	yes	3	5	0	1	3	3	3	3	3	4	1	3	3	2	3	2	2	3	2	3	2	2	3	1	2
1607	48	unmarried	less than 1,000,000 JPY	no	1	1	0	1	0	0	1	4	2	2	3	2	3	2	2	1	3	2	1	3	2	1	2	3	2
1608	55	unmarried	8,000,000-9,000,000 JPY	no	1	0	0	1	0	0	0	3	2	1	2	1	4	1	2	1	2	2	1	3	1	1	1	1	1
1609	63	married	18,000,000-20,000,000 JPY	yes	0	1	0	0	1	0	1	4	1	1	1	1	1	1	1	1	1	1	1	2	1	1	2	2	2
1610	81	married	3,000,000-4,000,000 JPY	yes	3	3	1	1	3	1	3	5	3	4	3	3	2	2	2	1	2	3	2	3	3	2	2	2	2
1611	63	unmarried	8,000,000-9,000,000 JPY	yes	1	1	0	0	0	0	1	5	2	3	3	4	2	1	1	1	2	4	2	3	3	3	4	1	3
1612	47	married	4,000,000-5,000,000 JPY	yes	3	3	3	3	3	3	2	5	3	3	3	3	3	3	3	3	3	3	3	3	3	3	3	3	3
1613	65	unmarried	less than 1,000,000 JPY	no	0	5	0	5	0	0	5	5	3	3	2	2	3	2	2	2	3	3	3	3	3	2	2	2	2
1614	63	married	4,000,000-5,000,000 JPY	yes	1	1	1	0	1	1	2	4	1	1	2	2	2	2	1	1	1	2	1	3	1	1	4	5	4
1615	57	unmarried	less than 1,000,000 JPY	no	0	0	0	0	0	0	0	0	1	1	1	1	1	1	1	1	1	1	1	1	1	1	1	1	1
1616	47	unmarried	3,000,000-4,000,000 JPY	no	5	5	0	0	0	0	2	3	1	1	1	2	2	1	1	1	5	1	1	1	1	1	1	2	1
1617	56	unmarried	4,000,000-5,000,000 JPY	no	0	1	0	0	0	0	0	1	1	2	1	1	2	1	1	1	1	2	1	3	2	1	1	1	1
1618	63	married	less than 1,000,000 JPY	yes	2	1	0	3	3	1	1	4	2	1	1	2	1	1	1	1	1	2	1	3	1	2	3	3	3
1619	68	married	2,000,000-3,000,000 JPY	yes	1	1	1	1	1	0	0	3	1	2	1	1	1	1	1	1	1	2	1	2	1	1	2	2	2
1620	73	married	6,000,000-7,000,000 JPY	yes	0	4	3	5	0	0	2	5	2	1	1	2	2	2	2	2	3	2	2	3	1	1	3	4	2
1621	74	married	3,000,000-4,000,000 JPY	no	2	3	2	2	5	4	5	5	2	2	1	2	2	1	1	1	2	2	1	2	1	1	4	4	4
1622	50	married	9,000,000-10,000,000 JPY	yes	0	3	0	0	0	0	1	2	1	3	2	1	1	2	2	1	2	2	2	1	1	1	1	1	2
1623	78	married	3,000,000-4,000,000 JPY	yes	2	2	3	2	1	1	2	4	2	2	1	3	2	2	2	1	2	3	1	2	1	1	4	4	4

1624	47	unmarried	1,000,000-2,000,000 JPY	no	0	0	0	0	0	0	1	3	3	4	4	5	3	3	2	3	4	5	3	1	3	3	2	3	3
1625	63	married	2,000,000-3,000,000 JPY	yes	0	1	1	1	2	2	2	3	2	3	4	4	4	4	4	4	4	4	4	4	3	3	4	3	3
1626	66	married	5,000,000-6,000,000 JPY	yes	0	0	0	0	1	0	5	2	1	2	1	1	1	1	1	1	1	1	1	2	1	1	2	2	1
1627	71	married	9,000,000-10,000,000 JPY	yes	1	0	0	0	2	0	2	4	2	2	1	2	2	1	1	1	1	2	1	2	1	1	2	2	2
1628	46	unmarried	4,000,000-5,000,000 JPY	no	1	0	0	0	1	0	1	1	1	1	1	2	1	5	1	1	1	1	3	5	4	1	3	4	3
1629	51	unmarried	less than 1,000,000 JPY	no	5	4	0	0	0	0	1	3	3	1	4	2	2	2	2	2	3	3	2	4	3	2	2	2	2
1630	57	married	4,000,000-5,000,000 JPY	no	1	0	0	0	1	0	1	3	2	2	2	3	3	2	2	1	2	2	1	3	2	1	2	3	3
1631	53	unmarried	1,000,000-2,000,000 JPY	no	0	0	0	0	0	0	1	0	1	1	1	2	1	2	1	1	1	1	1	1	1	1	1	1	1
1632	58	married	12,000,000-15,000,000 JPY	yes	1	0	0	0	1	1	0	3	3	3	2	2	3	1	1	2	2	2	1	2	1	1	4	4	4
1633	79	married	4,000,000-5,000,000 JPY	yes	0	0	0	0	0	0	5	0	1	1	1	1	1	1	1	1	1	1	1	1	1	1	1	1	1
1634	41	married	9,000,000-10,000,000 JPY	no	1	2	2	1	1	1	1	4	2	2	3	2	2	2	2	2	2	2	2	2	2	2	2	2	2
1635	53	unmarried	8,000,000-9,000,000 JPY	no	0	0	0	0	0	0	1	4	2	2	2	2	2	2	2	2	2	2	2	2	2	2	2	2	2
1636	65	married	7,000,000-8,000,000 JPY	yes	1	1	1	1	1	1	1	2	2	3	1	2	2	2	1	1	2	2	1	2	1	1	4	4	4
1637	57	married	3,000,000-4,000,000 JPY	no	1	1	0	0	1	0	2	4	1	1	1	1	2	1	1	1	1	1	1	2	2	1	2	2	2
1638	63	married	8,000,000-9,000,000 JPY	yes	0	1	0	0	1	0	0	2	1	2	1	3	2	2	1	1	3	3	1	4	2	1	4	5	3
1639	61	married	12,000,000-15,000,000 JPY	yes	0	4	0	2	0	0	1	3	1	1	1	2	1	1	2	1	1	2	1	1	1	1	1	2	1
1640	55	married	6,000,000-7,000,000 JPY	yes	0	1	0	0	1	0	0	1	2	1	1	3	2	2	2	2	1	1	2	1	1	1	1	3	2
1641	50	unmarried	1,000,000-2,000,000 JPY	no	0	0	0	0	0	0	5	3	1	2	1	1	1	1	1	1	1	2	2	1	1	1	1	1	1
1642	48	married	4,000,000-5,000,000 JPY	yes	0	1	1	1	4	3	0	4	2	1	2	2	2	2	1	1	3	2	2	3	1	1	2	2	2
1643	72	married	1,000,000-2,000,000 JPY	yes	1	2	2	0	3	2	1	3	3	3	2	3	3	2	1	1	2	2	1	2	1	1	1	2	2
1644	65	married	2,000,000-3,000,000 JPY	yes	1	1	1	1	1	1	1	3	2	2	1	1	1	1	1	1	1	1	1	1	1	1	1	1	1
1645	50	unmarried	1,000,000-2,000,000 JPY	no	0	0	0	0	0	0	0	0	1	1	1	2	1	1	1	1	1	1	1	1	1	1	1	1	1
1646	60	married	12,000,000-15,000,000 JPY	yes	5	3	5	5	5	5	2	5	3	2	3	3	2	2	3	3	2	3	4	3	3	2	2	3	3
1647	72	married	2,000,000-3,000,000 JPY	yes	0	2	5	5	5	1	3	4	1	1	1	2	1	2	1	1	2	3	2	3	3	1	5	5	4
1648	46	unmarried	3,000,000-4,000,000 JPY	no	0	2	2	2	1	3	1	3	2	2	2	2	2	3	2	1	2	3	2	2	2	2	3	2	2
1649	49	unmarried	1,000,000-2,000,000 JPY	no	0	1	0	0	0	0	1	2	2	2	2	1	2	2	1	1	2	2	2	2	1	1	3	5	4
1650	64	married	5,000,000-6,000,000 JPY	no	1	1	0	2	0	0	0	2	2	3	1	1	3	2	2	2	3	3	2	3	2	1	1	2	1
1651	71	married	3,000,000-4,000,000 JPY	yes	0	0	0	0	0	0	3	5	1	1	1	1	4	1	1	1	1	4	4	4	1	1	5	5	5
1652	41	married	4,000,000-5,000,000 JPY	no	2	5	4	4	3	0	2	4	3	2	2	3	2	2	3	3	3	2	3	4	1	1	3	3	3
1653	56	unmarried	7,000,000-8,000,000 JPY	no	1	4	1	2	3	0	1	3	3	3	1	3	2	2	2	1	2	3	1	1	1	1	2	3	1
1654	51	unmarried	4,000,000-5,000,000 JPY	no	0	0	0	0	0	0	0	3	1	1	1	1	1	1	1	1	1	1	1	1	1	1	1	1	1
1655	54	married	18,000,000-20,000,000 JPY	yes	1	2	3	3	2	1	5	5	3	4	3	4	2	3	4	3	2	2	3	4	4	3	2	2	3
1656	51	married	4,000,000-5,000,000 JPY	yes	0	1	0	1	5	1	0	3	1	2	1	1	2	1	1	1	1	2	1	2	1	1	2	1	1
1657	59	unmarried	8,000,000-9,000,000 JPY	no	0	0	0	0	0	0	0	0	1	1	1	2	1	1	1	2	2	2	1	2	1	1	1	1	1
1658	55	unmarried	1,000,000-2,000,000 JPY	no	1	2	1	2	0	0	1	3	2	2	1	2	2	2	1	1	3	3	2	4	2	1	2	3	1
1659	43	unmarried	4,000,000-5,000,000 JPY	yes	0	1	0	1	0	0	1	3	1	1	1	1	1	4	2	1	1	1	1	2	1	1	1	1	1
1660	78	married	less than 1,000,000 JPY	no	0	1	0	0	0	5	2	3	3	1	1	1	1	2	1	1	1	1	2	1	1	2	1	4	4
1661	60	married	7,000,000-8,000,000 JPY	yes	3	1	1	4	4	1	1	4	2	1	1	1	3	2	1	1	2	2	2	1	1	1	2	2	2
1662	58	unmarried	9,000,000-10,000,000 JPY	no	0	0	0	0	0	0	0	0	1	1	1	1	1	1	1	1	1	2	1	2	1	1	1	2	1
1663	52	married	10,000,000-12,000,000 JPY	yes	1	1	0	0	0	0	1	4	2	1	1	2	2	2	2	1	2	2	2	4	2	1	4	1	2
1664	79	unmarried	2,000,000-3,000,000 JPY	yes	2	2	0	0	0	0	1	3	4	4	1	3	2	1	1	1	2	3	3	4	3	3	4	4	4
1665	71	married	3,000,000-4,000,000 JPY	yes	1	0	0	1	1	1	1	1	1	1	1	1	2	2	2	1	1	1	2	1	2	1	2	2	2
1666	41	unmarried	less than 1,000,000 JPY	no	0	0	0	0	0	0	5	0	1	1	1	1	1	1	1	1	1	1	1	1	1	1	1	1	1
1667	58	unmarried	3,000,000-4,000,000 JPY	no	0	0	0	0	0	0	0	1	1	1	1	1	1	1	2	1	1	1	1	1	1	1	1	1	1
1668	77	married	2,000,000-3,000,000 JPY	yes	0	0	0	0	0	0	1	2	2	2	1	2	2	2	2	2	2	3	2	3	3	3	3	3	3
1669	71	unmarried	less than 1,000,000 JPY	no	0	0	0	0	0	0	1	3	1	1	1	1	1	1	1	1	1	1	1	1	1	1	1	1	1
1670	56	unmarried	5,000,000-6,000,000 JPY	no	1	1	0	1	0	0	1	1	3	4	3	4	3	3	3	3	3	3	3	3	2	3	5	5	5
1671	44	married	7,000,000-8,000,000 JPY	yes	0	0	0	0	0	0	0	0	1	1	1	1	2	1	1	1	1	1	1	1	1	1	1	1	1
1672	73	married	2,000,000-3,000,000 JPY	yes	4	4	1	3	3	3	3	5	3	2	1	1	3	3	3	3	3	2	2	3	1	1	2	2	2
1673	70	married	2,000,000-3,000,000 JPY	yes	1	5	4	1	2	0	5	4	3	3	3	4	3	3	3	1	1	2	2	3	3	3	3	3	3
1674	75	married	3,000,000-4,000,000 JPY	yes	2	5	1	1	4	1	3	3	3	2	2	3	2	2	2	1	3	3	2	3	2	3	3	4	3
1675	52	unmarried	3,000,000-4,000,000 JPY	no	0	0	0	0	0	0	0	3	1	1	1	1	1	1	1	1	1	1	1	1	1	1	1	1	1
1676	74	married	2,000,000-3,000,000 JPY	yes	0	0	0	0	0	0	0	1	1	1	1	1	2	1	1	1	1	1	1	1	1	1	1	1	1
1677	70	married	2,000,000-3,000,000 JPY	yes	1	1	1	1	2	0	1	4	2	1	1	2	1	1	2	1	2	2	1	2	1	1	2	2	2
1678	73	married	8,000,000-9,000,000 JPY	yes	0	1	0	0	0	0	1	1	2	2	1	2	2	1	1	1	1	2	1	2	1	1	2	2	2
1679	45	unmarried	4,000,000-5,000,000 JPY	no	5	4	5	5	2	2	5	6	4	5	5	5	5	4	3	2	3	3	3	5	4	1	5	4	4
1680	65	married	3,000,000-4,000,000 JPY	no	1	2	0	0	0	0	1	2	2	2	1	4	2	1	2	2	2	3	2	4	4	1	3	3	4
1681	72	married	3,000,000-4,000,000 JPY	yes	0	0	0	0	1	0	2	2	2	1	1	1	1	1	1	1	1	2	1	1	1	1	2	3	2

1682	50	unmarried	5,000,000-6,000,000 JPY	no	2	0	1	1	5	1	1	3	3	2	1	4	4	3	3	3	5	4	4	5	3	3	5	5	3
1683	58	married	12,000,000-15,000,000 JPY	yes	0	0	0	0	0	0	1	3	1	1	1	1	1	1	1	1	1	1	1	1	1	1	1	1	1
1684	82	married	5,000,000-6,000,000 JPY	yes	3	4	5	5	5	5	2	6	2	3	2	2	3	2	2	2	2	2	2	2	1	1	4	5	2
1685	68	married	3,000,000-4,000,000 JPY	yes	1	1	1	2	1	2	1	3	2	2	1	1	2	1	1	1	2	2	2	2	1	2	2	2	2
1686	75	married	2,000,000-3,000,000 JPY	yes	1	2	2	1	5	0	2	6	2	3	1	2	1	1	2	1	2	2	1	5	4	3	5	5	5
1687	45	unmarried	1,000,000-2,000,000 JPY	no	3	3	2	1	1	1	1	4	5	4	2	4	5	4	3	2	3	2	2	2	1	4	1	1	1
1688	61	married	less than 1,000,000 JPY	no	1	1	5	0	5	0	0	3	4	4	1	5	4	5	5	1	4	5	4	5	2	4	3	3	3
1689	72	married	less than 1,000,000 JPY	yes	0	1	1	0	0	0	1	3	1	3	3	3	3	2	2	2	2	3	3	2	3	3	3	1	1
1690	57	married	10,000,000-12,000,000 JPY	yes	1	1	1	1	2	2	1	3	3	2	2	2	2	2	2	2	2	2	2	4	3	2	3	3	2
1691	49	married	8,000,000-9,000,000 JPY	yes	1	1	2	0	1	2	1	4	3	3	1	1	1	2	2	2	2	1	1	3	1	1	1	1	1
1692	47	unmarried	3,000,000-4,000,000 JPY	no	0	0	0	0	0	0	0	3	2	1	1	2	1	1	1	1	1	1	1	1	1	1	2	1	1
1693	61	married	6,000,000-7,000,000 JPY	yes	1	1	1	0	0	0	2	4	3	3	3	3	3	3	3	3	3	4	3	3	3	3	3	3	3
1694	55	unmarried	6,000,000-7,000,000 JPY	no	1	1	1	1	0	0	0	2	2	2	1	1	1	1	1	1	2	2	1	1	1	1	1	1	1
1695	41	married	3,000,000-4,000,000 JPY	yes	0	1	2	0	2	2	0	3	1	1	1	3	3	1	1	1	3	3	3	1	1	1	1	2	1
1696	64	married	8,000,000-9,000,000 JPY	yes	1	2	0	2	2	1	1	3	3	3	3	3	3	3	3	3	3	3	3	2	3	2	3	2	2
1697	76	married	4,000,000-5,000,000 JPY	yes	0	0	0	0	0	0	1	1	1	1	1	1	1	1	1	1	1	2	1	1	1	1	1	1	1
1698	56	married	12,000,000-15,000,000 JPY	yes	1	1	0	0	0	0	1	2	1	2	1	2	1	1	1	1	1	1	1	1	1	1	2	2	2
1699	57	married	10,000,000-12,000,000 JPY	yes	0	2	0	0	0	0	0	1	1	2	1	1	1	1	1	1	1	1	1	1	1	1	1	1	1
1700	44	unmarried	3,000,000-4,000,000 JPY	no	0	3	0	1	0	0	0	3	3	2	2	2	2	2	2	2	3	3	3	4	4	2	3	3	2
1701	73	married	4,000,000-5,000,000 JPY	yes	3	3	3	3	3	3	3	3	2	2	1	2	2	2	2	2	2	2	2	2	2	2	3	4	3
1702	66	married	4,000,000-5,000,000 JPY	yes	5	1	5	5	5	5	1	6	3	1	1	3	2	3	3	1	3	3	3	5	4	2	5	5	5
1703	52	married	7,000,000-8,000,000 JPY	yes	0	1	1	0	0	0	1	3	1	1	1	2	2	1	1	1	2	1	1	3	1	1	1	2	1
1704	44	unmarried	2,000,000-3,000,000 JPY	no	0	0	0	0	0	0	0	0	2	2	2	2	1	1	3	2	3	3	3	2	1	1	1	1	1
1705	64	married	3,000,000-4,000,000 JPY	yes	0	0	0	0	0	0	0	0	1	1	1	1	1	1	1	1	1	1	1	2	1	1	1	2	1
1706	48	married	9,000,000-10,000,000 JPY	yes	0	0	0	0	0	0	5	2	1	1	1	1	1	1	1	1	1	1	1	1	1	1	1	1	1
1707	61	married	1,000,000-2,000,000 JPY	yes	5	5	5	5	5	5	4	6	4	2	2	3	5	2	2	3	3	3	3	2	3	2	2	3	2
1708	44	unmarried	3,000,000-4,000,000 JPY	no	1	1	0	0	2	0	2	4	3	3	2	4	5	4	5	5	5	3	5	5	5	1	3	1	1
1709	40	unmarried	2,000,000-3,000,000 JPY	no	0	0	0	0	0	0	0	1	1	1	3	3	2	2	2	2	2	2	3	2	2	1	2	2	3
1710	69	unmarried	less than 1,000,000 JPY	no	0	4	0	0	0	0	0	3	2	1	3	2	3	1	3	2	3	3	2	5	1	1	1	2	1
1711	45	unmarried	1,000,000-2,000,000 JPY	no	1	1	1	0	0	0	0	3	1	2	1	1	2	2	1	1	2	1	1	1	1	1	2	2	2
1712	61	married	5,000,000-6,000,000 JPY	yes	0	1	0	0	0	0	4	5	1	1	1	4	1	1	1	1	2	2	1	2	1	1	1	3	2
1713	53	married	6,000,000-7,000,000 JPY	yes	0	1	0	0	5	3	1	3	1	2	1	2	1	2	2	2	3	4	2	5	3	4	4	4	3
1714	67	unmarried	5,000,000-6,000,000 JPY	no	1	0	0	0	0	0	2	5	1	1	1	1	1	1	1	1	1	2	1	3	1	1	3	3	3
1715	55	married	1,000,000-2,000,000 JPY	no	0	0	0	0	0	0	0	0	1	1	1	1	1	1	1	1	1	1	1	1	1	1	1	1	1
1716	42	married	5,000,000-6,000,000 JPY	yes	5	3	0	1	0	0	3	2	1	1	1	2	2	2	1	1	1	1	1	2	2	1	2	2	1
1717	64	married	5,000,000-6,000,000 JPY	yes	5	0	5	0	4	0	1	5	3	1	1	2	3	1	1	1	2	3	1	3	1	3	4	4	4
1718	62	married	5,000,000-6,000,000 JPY	yes	1	1	0	1	1	0	1	3	1	1	1	1	2	2	2	1	1	2	1	1	1	1	2	2	2
1719	57	married	5,000,000-6,000,000 JPY	yes	5	5	5	5	5	5	2	5	1	2	1	1	2	1	1	1	2	2	1	2	1	1	2	2	2
1720	63	married	3,000,000-4,000,000 JPY	no	0	1	0	1	0	0	1	4	1	1	1	1	2	2	1	1	1	2	2	2	1	1	1	5	2
1721	40	married	4,000,000-5,000,000 JPY	no	0	1	0	1	0	0	1	3	3	3	1	3	3	3	1	1	2	3	2	3	2	1	1	1	3
1722	62	married	9,000,000-10,000,000 JPY	yes	1	2	0	1	4	0	1	4	3	1	1	3	2	2	2	2	2	2	2	4	2	1	3	4	3
1723	65	married	less than 1,000,000 JPY	yes	0	0	0	0	3	0	1	3	2	2	3	2	2	2	2	1	1	2	1	2	1	1	2	2	2
1724	41	married	5,000,000-6,000,000 JPY	yes	0	0	0	0	0	0	1	1	1	1	1	1	1	1	1	1	1	1	1	1	1	1	1	1	1
1725	51	married	1,000,000-2,000,000 JPY	yes	5	4	3	2	1	0	3	4	5	2	1	4	5	1	1	1	4	3	2	5	5	1	3	2	2
1726	47	unmarried	3,000,000-4,000,000 JPY	no	0	1	0	1	1	0	0	0	1	2	2	2	1	2	3	1	3	4	2	1	1	1	1	3	1
1727	42	unmarried	4,000,000-5,000,000 JPY	no	0	0	0	0	0	0	0	0	1	1	1	1	1	1	1	1	1	1	1	1	1	1	1	1	1
1728	50	married	6,000,000-7,000,000 JPY	yes	0	0	0	0	0	0	3	1	1	1	1	1	1	1	1	1	1	1	1	1	1	1	1	1	1
1729	51	married	6,000,000-7,000,000 JPY	no	1	1	1	1	2	2	1	3	1	1	1	3	2	2	2	2	2	3	2	3	2	1	3	3	3
1730	52	married	4,000,000-5,000,000 JPY	yes	1	1	0	0	0	1	1	2	2	2	1	1	2	1	1	1	2	2	2	2	2	1	1	2	1
1731	59	unmarried	3,000,000-4,000,000 JPY	no	1	1	1	0	1	0	1	2	2	2	1	1	1	1	1	1	2	2	1	2	1	1	2	2	2
1732	47	married	10,000,000-12,000,000 JPY	yes	0	0	0	0	0	0	0	2	1	1	1	1	1	1	1	1	1	1	1	1	1	1	1	1	1
1733	62	married	9,000,000-10,000,000 JPY	yes	0	0	0	0	0	0	1	2	2	3	1	2	1	1	2	2	2	2	2	3	2	2	2	2	2
1734	62	married	6,000,000-7,000,000 JPY	yes	0	0	0	0	0	0	1	3	2	1	1	1	1	1	1	1	1	1	1	1	1	1	1	1	1
1735	60	married	10,000,000-12,000,000 JPY	no	0	0	0	0	0	0	0	1	1	1	1	1	1	1	1	1	1	1	1	1	1	1	1	1	1
1736	63	married	3,000,000-4,000,000 JPY	yes	1	1	1	0	1	1	1	4	2	3	1	3	2	1	1	2	1	2	1	2	1	1	2	2	3
1737	56	unmarried	3,000,000-4,000,000 JPY	no	2	3	0	3	3	0	3	3	1	1	1	3	3	1	1	1	3	3	1	3	1	1	3	3	3
1738	44	unmarried	10,000,000-12,000,000 JPY	no	0	0	0	0	0	0	0	3	3	4	1	3	2	2	2	1	1	1	1	1	1	1	1	1	1
1739	64	married	15,000,000-18,000,000 JPY	yes	0	0	0	0	0	0	3	5	1	3	1	2	2	2	1	1	1	1	1	1	1	1	3	3	2

1740	48	married	6,000,000-7,000,000 JPY	yes	5	5	0	0	0	0	0	3	3	3	3	3	3	3	3	3	3	3	3	3	3	3	3	3	3
1741	49	unmarried	2,000,000-3,000,000 JPY	no	1	1	0	0	0	1	1	2	2	2	1	3	2	2	2	2	2	3	1	3	1	1	3	3	2
1742	48	unmarried	less than 1,000,000 JPY	no	0	0	0	0	0	0	0	0	3	2	1	5	5	1	5	1	1	1	1	1	5	1	1	1	1
1743	50	married	1,000,000-2,000,000 JPY	yes	0	0	0	0	0	0	4	1	3	2	2	3	2	2	2	2	3	2	2	3	2	1	2	2	1
1744	59	unmarried	4,000,000-5,000,000 JPY	no	0	1	0	0	0	0	1	3	1	1	1	1	1	1	1	1	1	1	1	2	1	1	2	3	4
1745	42	unmarried	4,000,000-5,000,000 JPY	no	1	1	2	0	2	3	1	5	3	1	2	2	3	2	2	1	4	1	3	4	2	2	4	4	2
1746	48	unmarried	6,000,000-7,000,000 JPY	no	0	0	0	0	0	0	0	0	1	1	1	1	1	1	1	1	1	1	1	1	1	1	1	1	1
1747	48	married	9,000,000-10,000,000 JPY	yes	0	0	0	0	0	0	0	3	1	3	1	1	1	1	1	1	1	1	1	3	2	1	2	2	2
1748	60	unmarried	1,000,000-2,000,000 JPY	no	2	5	5	4	5	5	1	6	4	4	2	5	3	5	5	5	4	4	5	5	4	2	4	4	4
1749	64	unmarried	1,000,000-2,000,000 JPY	no	0	3	0	0	5	2	1	4	3	2	3	5	3	1	4	3	3	4	3	5	2	1	5	5	1
1750	43	unmarried	4,000,000-5,000,000 JPY	no	1	3	1	0	1	1	1	3	1	1	1	1	1	1	1	1	1	1	1	2	1	1	2	2	2
1751	68	unmarried	3,000,000-4,000,000 JPY	no	3	0	1	0	0	0	1	3	1	1	1	1	1	1	1	1	1	1	1	1	1	1	1	1	1
1752	58	married	5,000,000-6,000,000 JPY	yes	1	1	0	1	0	0	0	3	1	1	1	1	1	1	1	1	1	2	1	1	1	1	1	2	2
1753	43	married	7,000,000-8,000,000 JPY	no	0	0	0	0	0	0	1	3	2	1	1	3	3	4	3	4	1	1	1	1	3	1	3	1	1
1754	65	married	4,000,000-5,000,000 JPY	yes	5	5	0	0	5	5	1	3	1	1	1	1	2	2	1	1	2	1	1	4	1	1	3	3	4
1755	60	married	9,000,000-10,000,000 JPY	yes	0	0	0	0	0	0	1	3	1	2	1	1	2	1	1	1	2	2	1	3	1	1	1	1	1
1756	68	married	3,000,000-4,000,000 JPY	yes	1	1	1	1	3	2	1	4	2	2	1	2	2	1	1	1	2	4	1	2	1	1	3	3	2
1757	41	unmarried	1,000,000-2,000,000 JPY	no	1	1	0	0	1	0	0	5	3	1	2	4	4	2	2	4	4	4	2	5	5	1	4	5	4
1758	54	married	7,000,000-8,000,000 JPY	yes	4	3	5	4	3	2	5	5	2	2	2	5	2	2	3	1	3	3	3	5	4	1	4	5	4
1759	48	married	8,000,000-9,000,000 JPY	yes	2	2	1	2	3	3	2	1	3	3	2	2	4	3	2	4	3	2	2	2	4	3	2	2	2
1760	52	unmarried	12,000,000-15,000,000 JPY	no	0	1	0	0	1	0	0	1	3	3	1	1	2	1	1	1	2	3	1	3	1	1	2	3	2
1761	53	married	10,000,000-12,000,000 JPY	yes	1	1	0	1	3	0	0	3	2	2	2	2	2	2	1	1	2	2	1	1	1	1	1	1	2
1762	40	unmarried	less than 1,000,000 JPY	no	4	4	1	4	4	0	3	4	3	1	1	3	4	4	4	1	4	3	4	5	4	1	3	4	3
1763	63	married	2,000,000-3,000,000 JPY	yes	1	4	4	1	3	1	1	5	3	1	1	1	1	1	1	1	2	2	2	3	1	1	2	2	1
1764	40	unmarried	12,000,000-15,000,000 JPY	no	0	1	0	0	0	0	1	3	2	1	3	3	3	2	2	2	3	1	2	3	2	1	2	2	2
1765	43	married	6,000,000-7,000,000 JPY	yes	0	0	0	0	0	0	4	1	1	1	1	1	2	1	1	1	1	3	1	5	1	1	3	4	3
1766	53	married	5,000,000-6,000,000 JPY	yes	0	0	0	0	1	0	1	4	2	2	2	3	2	1	1	1	2	3	2	3	1	1	3	2	2
1767	65	married	3,000,000-4,000,000 JPY	yes	0	5	0	0	0	0	1	3	5	5	1	5	4	1	5	3	5	5	5	5	5	1	5	5	5
1768	54	married	12,000,000-15,000,000 JPY	yes	2	2	0	5	1	0	5	6	3	1	3	3	3	1	1	1	2	1	1	3	1	1	1	1	1
1769	44	married	10,000,000-12,000,000 JPY	yes	1	3	1	0	1	0	2	5	1	1	1	1	2	2	1	2	2	1	2	1	1	1	1	2	1
1770	59	married	4,000,000-5,000,000 JPY	yes	1	1	4	1	5	4	5	4	3	4	1	2	2	5	3	3	3	3	3	5	4	2	5	1	3
1771	53	married	3,000,000-4,000,000 JPY	yes	1	3	2	4	4	1	1	6	2	2	3	3	3	3	2	2	3	2	3	3	2	1	3	2	1
1772	49	married	9,000,000-10,000,000 JPY	yes	0	0	0	0	0	0	1	5	2	4	3	2	3	2	3	1	3	3	3	4	3	3	3	2	2
1773	49	unmarried	4,000,000-5,000,000 JPY	no	1	1	0	0	0	0	1	3	2	2	3	2	3	3	3	4	3	3	3	5	3	3	3	1	3
1774	66	married	3,000,000-4,000,000 JPY	yes	1	1	1	1	2	2	1	2	1	1	1	1	1	2	1	1	2	1	1	2	1	1	1	3	2
1775	52	married	8,000,000-9,000,000 JPY	yes	1	1	0	0	1	0	1	1	1	1	1	1	1	2	1	1	1	2	1	3	1	1	3	2	3
1776	46	unmarried	5,000,000-6,000,000 JPY	no	1	1	1	0	1	1	1	2	2	2	1	1	3	2	2	2	2	2	3	3	2	1	2	2	2
1777	41	unmarried	2,000,000-3,000,000 JPY	no	3	3	4	3	3	2	3	4	3	3	3	3	3	3	3	4	3	3	2	3	3	2	3	3	3
1778	51	unmarried	4,000,000-5,000,000 JPY	yes	0	2	0	0	0	0	1	2	1	1	1	1	1	1	1	1	1	2	1	2	1	1	2	2	2
1779	44	unmarried	1,000,000-2,000,000 JPY	no	0	0	0	0	0	0	0	3	1	1	1	1	1	1	1	1	1	1	1	1	1	1	1	1	1
1780	67	married	3,000,000-4,000,000 JPY	yes	0	5	0	5	5	5	3	5	1	1	1	1	1	1	1	1	1	1	1	1	1	1	2	2	1
1781	47	married	7,000,000-8,000,000 JPY	yes	0	0	0	0	0	0	0	0	2	1	3	1	2	1	1	1	1	1	1	3	1	1	1	1	1
1782	55	unmarried	4,000,000-5,000,000 JPY	no	2	2	0	1	0	0	1	3	2	2	2	3	3	3	2	2	2	2	2	2	2	2	2	2	2
1783	49	unmarried	less than 1,000,000 JPY	no	0	0	0	0	0	0	0	3	2	1	1	3	1	3	2	1	4	5	4	5	5	2	5	5	5
1784	58	unmarried	2,000,000-3,000,000 JPY	yes	1	0	0	0	1	0	1	3	2	2	2	1	2	1	1	2	3	3	2	3	2	1	2	3	2
1785	57	married	10,000,000-12,000,000 JPY	yes	1	4	1	0	4	1	3	5	3	3	1	1	2	2	1	1	2	3	1	3	1	1	1	1	1
1786	52	married	7,000,000-8,000,000 JPY	yes	1	0	0	0	1	0	3	3	1	2	1	1	1	1	1	1	1	2	1	1	1	1	2	1	1
1787	65	married	3,000,000-4,000,000 JPY	yes	0	0	0	1	0	0	1	3	1	1	1	1	1	2	2	1	1	1	1	3	1	1	2	2	1
1788	43	unmarried	2,000,000-3,000,000 JPY	no	1	0	0	0	0	0	0	0	1	1	1	1	1	1	1	1	1	1	1	1	1	1	1	1	1
1789	65	married	2,000,000-3,000,000 JPY	yes	0	0	0	0	0	0	1	1	1	2	1	1	1	1	1	1	1	1	1	1	1	1	1	1	1
1790	52	unmarried	4,000,000-5,000,000 JPY	no	1	4	1	2	1	0	1	6	1	1	1	2	2	1	1	1	2	2	1	2	1	1	2	2	1
1791	60	married	10,000,000-12,000,000 JPY	yes	3	1	3	1	3	3	2	3	4	1	2	3	1	1	1	1	3	3	1	5	1	1	5	5	5
1792	58	married	2,000,000-3,000,000 JPY	no	0	0	0	0	0	0	0	0	1	4	1	1	1	1	1	1	1	1	1	1	1	1	1	1	3
1793	41	unmarried	less than 1,000,000 JPY	no	0	0	0	0	0	0	1	1	1	1	1	2	1	2	2	1	2	2	2	1	2	2	2	2	2
1794	42	unmarried	8,000,000-9,000,000 JPY	no	3	1	0	0	0	0	1	3	2	3	2	3	2	2	2	1	2	2	1	2	1	1	1	1	1
1795	40	unmarried	4,000,000-5,000,000 JPY	no	0	0	0	0	0	0	0	0	1	1	1	1	1	1	1	1	1	1	1	1	1	1	1	1	1
1796	53	married	6,000,000-7,000,000 JPY	yes	5	2	0	0	1	0	0	2	2	2	1	1	3	2	2	1	3	3	1	3	1	1	3	4	4
1797	49	unmarried	5,000,000-6,000,000 JPY	no	0	1	0	0	0	0	4	0	1	1	1	2	1	2	1	1	2	2	1	2	2	1	2	3	2

1798	41	unmarried	6,000,000-7,000,000 JPY	no	0	0	0	0	0	0	4	3	1	1	1	1	1	1	1	2	1	1	2	2	2	1	1	1	1
1799	47	unmarried	4,000,000-5,000,000 JPY	no	1	2	3	4	5	5	2	2	2	3	3	3	2	3	3	4	2	3	4	3	4	4	2	3	4
1800	54	married	10,000,000-12,000,000 JPY	no	0	1	1	0	2	1	2	3	3	1	2	3	3	2	2	1	1	2	1	3	2	2	3	3	3
1801	42	unmarried	4,000,000-5,000,000 JPY	no	2	0	0	0	4	2	1	5	1	1	1	4	2	2	2	1	2	1	3	2	1	1	2	2	2
1802	62	married	18,000,000-20,000,000 JPY	yes	2	1	2	4	5	2	0	4	3	2	2	3	4	2	3	2	3	2	2	4	3	2	2	1	1
1803	55	married	9,000,000-10,000,000 JPY	yes	1	1	0	1	0	0	2	3	2	2	1	1	2	1	1	1	1	1	1	2	1	1	2	2	2
1804	40	married	8,000,000-9,000,000 JPY	yes	0	0	0	0	0	0	0	0	1	1	1	1	1	1	1	1	1	1	1	1	1	1	1	1	1
1805	69	married	18,000,000-20,000,000 JPY	yes	0	5	0	5	0	0	3	4	1	1	1	1	2	1	1	1	1	1	1	2	1	1	2	2	2
1806	47	unmarried	1,000,000-2,000,000 JPY	no	0	0	0	0	0	0	0	3	1	1	1	1	1	1	1	1	1	1	1	1	1	1	1	1	1
1807	40	unmarried	3,000,000-4,000,000 JPY	no	0	0	0	0	0	0	1	1	1	1	1	1	3	2	1	1	1	1	1	1	1	1	1	4	1
1808	63	unmarried	2,000,000-3,000,000 JPY	yes	1	1	0	3	3	0	0	3	3	4	2	4	4	3	3	3	4	4	4	5	4	1	5	5	5
1809	48	unmarried	20,000,000 or more JPY	no	0	0	0	0	0	0	1	3	2	2	1	2	3	3	1	1	3	3	1	4	3	1	3	5	4
1810	47	unmarried	6,000,000-7,000,000 JPY	no	0	0	0	0	0	0	1	3	1	1	1	1	1	1	1	1	1	1	3	1	1	1	1	1	1
1811	46	married	4,000,000-5,000,000 JPY	yes	3	3	5	4	4	4	1	6	4	4	4	4	4	4	4	4	4	4	4	4	4	4	3	3	4
1812	54	unmarried	7,000,000-8,000,000 JPY	no	1	0	0	0	0	0	1	3	3	3	3	3	3	3	3	3	3	3	3	3	3	3	3	3	3
1813	49	married	10,000,000-12,000,000 JPY	yes	0	0	0	0	0	0	0	1	1	1	1	1	1	1	1	1	1	1	1	1	1	1	1	1	1
1814	59	married	7,000,000-8,000,000 JPY	no	0	0	0	0	0	0	0	0	1	1	1	1	1	1	1	1	1	1	1	1	1	1	1	1	1
1815	55	married	5,000,000-6,000,000 JPY	yes	0	0	0	0	0	0	0	3	1	2	1	5	1	1	1	1	1	3	1	1	1	1	1	1	1
1816	58	married	less than 1,000,000 JPY	yes	0	1	0	1	1	1	1	3	4	1	1	1	2	2	1	1	2	2	1	2	1	1	1	2	1
1817	49	married	3,000,000-4,000,000 JPY	yes	0	0	0	0	0	0	0	2	1	1	1	1	1	1	1	1	1	1	1	1	1	1	1	1	1
1818	61	married	6,000,000-7,000,000 JPY	yes	1	1	0	1	1	0	5	3	1	1	1	1	2	1	1	1	1	2	2	2	1	1	1	1	1
1819	52	married	6,000,000-7,000,000 JPY	yes	4	1	0	1	0	0	0	3	1	1	1	1	1	2	1	1	2	4	1	1	1	1	3	1	2
1820	61	married	5,000,000-6,000,000 JPY	yes	1	2	1	1	1	0	1	4	2	3	2	4	3	3	2	2	2	3	2	2	1	1	2	2	3
1821	56	unmarried	3,000,000-4,000,000 JPY	no	2	0	3	0	5	3	1	4	3	4	4	4	3	2	2	2	4	4	1	4	2	1	4	5	3
1822	58	married	7,000,000-8,000,000 JPY	yes	0	0	0	0	0	0	1	3	1	2	1	1	1	2	1	1	1	1	1	1	1	1	2	3	2
1823	62	married	3,000,000-4,000,000 JPY	yes	0	1	1	0	1	1	1	2	1	2	1	1	2	1	1	1	1	1	1	3	1	1	3	3	3
1824	51	unmarried	3,000,000-4,000,000 JPY	no	0	1	0	0	1	0	1	1	2	1	1	3	2	3	2	2	2	2	2	2	1	1	2	3	2
1825	69	unmarried	less than 1,000,000 JPY	no	1	5	0	0	5	0	5	2	2	1	1	1	2	1	1	1	1	3	1	3	1	4	3	3	3
1826	59	married	6,000,000-7,000,000 JPY	no	0	0	0	0	0	0	1	3	1	3	2	2	1	1	1	1	1	2	1	2	1	2	1	1	1
1827	65	married	10,000,000-12,000,000 JPY	yes	3	1	4	0	3	3	2	5	2	1	1	3	1	1	1	1	2	2	2	3	1	1	4	2	4
1828	69	married	3,000,000-4,000,000 JPY	yes	0	1	0	1	1	0	0	1	1	1	1	1	1	1	1	1	1	1	1	2	1	1	1	1	1
1829	63	married	8,000,000-9,000,000 JPY	no	4	5	1	0	4	4	3	5	3	3	1	2	2	1	1	1	2	4	1	5	3	1	5	5	5
1830	54	unmarried	6,000,000-7,000,000 JPY	yes	0	1	0	0	1	0	0	2	1	2	2	1	1	1	1	1	1	1	1	1	1	1	2	3	1
1831	55	married	7,000,000-8,000,000 JPY	yes	0	0	0	0	0	0	0	0	2	2	1	2	2	3	3	4	2	2	2	2	2	1	2	3	2
1832	63	married	20,000,000 or more JPY	yes	1	1	0	0	0	0	0	3	2	1	1	1	2	1	1	1	2	3	2	3	3	2	3	3	3
1833	42	married	7,000,000-8,000,000 JPY	yes	0	0	0	0	0	0	0	1	2	1	1	2	1	1	1	1	1	1	1	1	1	1	4	4	3
1834	54	married	6,000,000-7,000,000 JPY	yes	0	0	0	0	0	0	1	3	1	1	1	1	1	1	1	1	1	1	1	1	1	1	1	1	1
1835	46	married	4,000,000-5,000,000 JPY	yes	3	4	2	1	0	0	5	3	1	1	3	2	2	2	2	2	2	2	2	2	1	1	1	2	1
1836	50	unmarried	1,000,000-2,000,000 JPY	no	5	5	4	5	5	5	0	6	5	5	5	5	5	5	5	1	5	5	1	5	5	1	4	3	2
1837	66	married	2,000,000-3,000,000 JPY	yes	0	0	0	0	0	0	0	1	1	1	1	1	1	1	1	1	1	1	1	1	1	1	2	1	2
1838	55	unmarried	9,000,000-10,000,000 JPY	no	0	0	0	0	0	0	1	1	1	1	1	1	1	1	1	1	1	2	1	2	1	1	1	1	1
1839	43	married	4,000,000-5,000,000 JPY	yes	1	1	1	1	1	1	1	2	2	2	1	2	2	2	2	1	1	2	1	4	1	1	3	2	3
1840	65	married	2,000,000-3,000,000 JPY	yes	5	1	1	0	5	1	2	5	2	2	1	2	2	2	1	1	3	3	2	3	1	1	1	3	2
1841	54	unmarried	less than 1,000,000 JPY	no	1	1	1	0	1	0	1	3	4	4	1	4	3	2	3	4	4	4	4	4	3	2	4	5	3
1842	59	married	12,000,000-15,000,000 JPY	yes	0	1	0	0	0	0	1	3	1	1	1	2	1	1	1	1	2	1	1	1	1	1	1	1	1
1843	49	married	4,000,000-5,000,000 JPY	yes	0	1	0	0	0	0	1	3	2	3	1	3	3	1	3	3	2	2	2	3	3	1	1	1	1
1844	48	married	7,000,000-8,000,000 JPY	no	0	1	0	0	0	0	0	0	1	1	1	1	1	1	1	1	1	1	1	1	1	1	1	1	1
1845	66	unmarried	1,000,000-2,000,000 JPY	yes	0	0	0	0	0	0	1	3	2	3	3	2	1	1	1	1	1	3	1	2	2	1	2	2	1
1846	45	married	3,000,000-4,000,000 JPY	yes	1	1	0	0	0	0	2	3	1	1	1	1	1	1	1	1	1	1	1	1	1	1	1	1	1
1847	60	married	12,000,000-15,000,000 JPY	yes	3	3	1	1	1	1	2	3	2	2	2	1	2	2	2	1	2	2	2	3	2	2	2	2	2
1848	62	married	1,000,000-2,000,000 JPY	yes	1	1	1	1	1	0	1	3	2	2	1	1	1	1	1	1	2	2	2	3	1	1	1	2	1
1849	48	unmarried	3,000,000-4,000,000 JPY	no	0	0	0	0	0	0	0	3	1	1	1	1	1	1	1	1	1	1	1	1	1	1	1	1	1
1850	69	unmarried	2,000,000-3,000,000 JPY	no	2	2	2	1	2	2	2	3	2	3	2	2	2	2	2	1	2	2	2	2	2	2	2	2	2
1851	47	married	5,000,000-6,000,000 JPY	yes	0	0	0	0	0	0	1	1	1	1	1	1	1	1	1	1	1	1	1	1	1	1	1	1	1
1852	46	unmarried	10,000,000-12,000,000 JPY	no	0	1	0	0	0	0	0	1	1	2	1	1	2	1	1	1	1	1	1	2	1	1	1	1	1
1853	47	unmarried	1,000,000-2,000,000 JPY	yes	3	2	2	1	2	1	2	4	3	3	1	2	2	2	2	2	3	2	3	3	2	2	2	2	3
1854	67	unmarried	2,000,000-3,000,000 JPY	no	0	0	0	0	0	0	0	0	2	1	2	2	3	1	1	1	1	2	2	2	1	1	2	3	2
1855	69	married	3,000,000-4,000,000 JPY	yes	0	1	1	0	1	0	3	4	2	2	1	1	1	1	1	1	2	2	1	3	2	1	2	3	3

1856	44	married	5,000,000-6,000,000 JPY	yes	0	0	0	0	0	0	0	2	1	1	1	1	1	1	1	1	1	1	1	1	1	1	1	1	1
1857	40	married	10,000,000-12,000,000 JPY	yes	0	0	0	0	0	0	0	3	1	3	1	1	2	2	1	1	1	1	1	2	1	1	1	1	1
1858	42	married	4,000,000-5,000,000 JPY	yes	1	1	0	0	0	0	1	4	1	1	1	2	2	2	1	1	2	1	1	1	1	1	1	1	2
1859	40	married	3,000,000-4,000,000 JPY	no	4	3	5	1	1	2	1	5	3	4	2	4	4	2	3	2	3	3	2	3	1	3	1	3	1
1860	47	unmarried	3,000,000-4,000,000 JPY	no	0	0	0	0	0	0	0	0	1	2	1	1	1	1	1	1	1	1	1	2	1	1	1	1	1
1861	61	married	3,000,000-4,000,000 JPY	yes	0	0	1	0	5	0	1	3	3	3	3	3	2	3	2	2	2	3	1	4	4	2	5	5	5
1862	63	married	less than 1,000,000 JPY	no	1	1	0	0	1	0	1	2	2	2	1	2	2	1	1	1	2	2	1	2	1	1	2	2	2
1863	66	married	3,000,000-4,000,000 JPY	yes	1	5	1	4	4	1	2	4	2	2	1	2	1	2	1	1	1	1	1	3	2	2	2	2	2
1864	47	married	6,000,000-7,000,000 JPY	yes	4	4	3	3	3	3	4	4	4	3	4	4	4	4	4	3	4	4	4	3	4	4	4	3	4
1865	48	unmarried	15,000,000-18,000,000 JPY	no	1	2	0	0	0	1	0	0	1	1	1	2	3	1	1	1	1	2	1	1	1	1	3	2	3
1866	69	married	6,000,000-7,000,000 JPY	yes	0	1	2	2	3	2	2	3	2	2	1	2	2	2	1	1	1	2	1	2	1	1	1	1	1
1867	64	married	7,000,000-8,000,000 JPY	yes	0	2	0	0	0	0	1	2	1	1	1	1	1	1	1	1	2	2	2	2	1	1	1	1	1
1868	49	married	8,000,000-9,000,000 JPY	yes	0	0	0	0	1	0	0	2	1	2	2	3	3	3	3	3	2	3	3	4	2	3	2	2	2
1869	65	unmarried	less than 1,000,000 JPY	no	0	3	0	0	0	0	1	3	1	2	2	3	3	2	2	2	2	2	3	2	1	1	1	1	1
1870	42	unmarried	7,000,000-8,000,000 JPY	no	0	0	0	0	0	0	0	0	1	1	1	2	1	1	1	1	1	1	1	1	1	1	1	1	1
1871	61	married	6,000,000-7,000,000 JPY	yes	0	1	0	0	1	1	1	3	1	2	1	1	1	2	1	1	2	2	1	3	1	1	2	3	2
1872	68	married	3,000,000-4,000,000 JPY	yes	0	1	0	1	1	0	1	4	2	1	1	1	1	1	1	1	1	2	1	2	1	1	2	2	1
1873	49	unmarried	1,000,000-2,000,000 JPY	no	0	0	0	0	0	0	0	3	1	1	1	1	1	1	1	1	1	1	1	1	1	1	1	1	1
1874	47	unmarried	4,000,000-5,000,000 JPY	no	0	1	0	0	1	0	0	2	1	3	2	2	2	2	1	1	2	3	2	2	1	1	1	1	1
1875	45	married	8,000,000-9,000,000 JPY	yes	5	1	0	0	0	0	1	3	3	2	3	3	3	2	3	2	3	4	2	4	2	1	1	1	1
1876	45	married	5,000,000-6,000,000 JPY	yes	0	1	0	0	0	0	0	2	1	2	1	4	3	2	3	2	3	2	2	3	1	1	2	4	1
1877	49	married	10,000,000-12,000,000 JPY	yes	4	1	0	2	0	5	2	4	1	2	1	3	2	3	3	2	2	1	2	1	1	1	3	3	3
1878	43	married	7,000,000-8,000,000 JPY	yes	0	0	0	0	0	0	1	2	1	1	1	1	1	1	1	1	1	1	1	1	1	1	1	1	1
1879	68	married	2,000,000-3,000,000 JPY	yes	0	1	0	1	1	0	1	2	3	3	1	1	2	2	1	1	1	3	1	2	1	1	3	3	3
1880	65	married	7,000,000-8,000,000 JPY	yes	2	2	3	1	2	3	1	3	2	3	2	3	2	1	3	2	3	3	2	2	1	2	1	3	3
1881	47	married	4,000,000-5,000,000 JPY	yes	0	1	0	0	0	0	0	0	1	1	1	1	1	1	1	1	1	1	1	1	1	1	1	1	1
1882	40	unmarried	3,000,000-4,000,000 JPY	no	0	0	0	0	0	0	0	3	2	2	1	1	1	1	1	1	1	1	1	3	2	1	2	2	2
1883	60	married	9,000,000-10,000,000 JPY	yes	1	1	1	0	4	1	0	3	2	2	1	2	2	2	1	1	2	2	1	2	1	1	1	1	1
1884	63	married	7,000,000-8,000,000 JPY	yes	0	0	0	0	0	0	0	1	1	1	1	1	1	1	1	1	1	2	1	3	1	1	2	4	2
1885	46	married	8,000,000-9,000,000 JPY	no	0	0	0	0	0	0	0	3	1	1	1	1	1	1	1	1	1	1	1	1	1	1	1	1	1
1886	62	married	15,000,000-18,000,000 JPY	yes	3	3	0	0	0	0	1	5	2	2	1	3	3	1	1	1	2	2	1	3	1	1	2	2	2
1887	49	unmarried	3,000,000-4,000,000 JPY	no	0	0	0	0	0	0	0	3	1	1	1	1	1	1	1	1	1	1	1	1	1	1	1	1	1
1888	44	unmarried	2,000,000-3,000,000 JPY	no	1	4	1	1	1	1	1	3	1	2	1	2	1	3	3	2	1	1	1	2	1	1	2	2	2
1889	46	unmarried	6,000,000-7,000,000 JPY	yes	1	1	2	1	2	0	2	4	2	3	4	3	3	3	4	2	2	3	3	4	2	3	4	3	2
1890	67	married	3,000,000-4,000,000 JPY	no	0	0	0	0	0	0	1	1	1	1	1	1	1	1	1	1	1	2	1	2	1	1	2	2	2
1891	49	unmarried	1,000,000-2,000,000 JPY	no	0	0	0	0	0	0	0	3	1	1	1	1	1	1	1	1	1	1	1	1	1	1	1	1	1
1892	40	married	5,000,000-6,000,000 JPY	yes	0	3	0	1	0	0	0	4	1	1	1	2	2	2	1	1	2	4	2	1	1	1	1	1	1
1893	66	married	7,000,000-8,000,000 JPY	yes	0	0	0	0	0	0	5	3	1	1	1	1	2	1	1	1	1	1	1	2	1	1	1	1	1
1894	69	married	10,000,000-12,000,000 JPY	yes	0	0	0	0	0	0	0	1	1	2	1	2	1	1	1	1	2	2	1	2	1	1	2	2	2
1895	64	unmarried	less than 1,000,000 JPY	no	4	5	4	4	2	2	5	3	3	4	3	2	3	3	4	2	4	4	3	4	3	3	3	3	3
1896	68	married	3,000,000-4,000,000 JPY	yes	1	1	0	0	0	0	1	4	3	1	1	1	1	1	1	1	3	1	1	1	1	1	3	3	3
1897	61	married	7,000,000-8,000,000 JPY	yes	1	1	1	0	1	0	0	1	1	1	1	2	2	2	2	1	1	1	1	1	1	1	1	1	1
1898	44	unmarried	4,000,000-5,000,000 JPY	no	0	0	0	0	0	0	0	0	1	2	1	1	1	1	1	1	1	1	1	1	1	1	1	1	1
1899	48	married	6,000,000-7,000,000 JPY	yes	0	0	0	0	0	0	0	0	1	4	1	2	1	2	1	1	1	3	1	3	1	1	4	2	3
1900	61	married	2,000,000-3,000,000 JPY	yes	1	1	1	0	1	0	0	4	1	1	1	1	1	1	1	1	1	2	1	3	1	1	3	4	5
1901	43	married	3,000,000-4,000,000 JPY	yes	4	4	4	4	4	4	2	0	3	4	4	3	3	3	3	3	2	2	2	3	3	2	3	3	3
1902	68	married	4,000,000-5,000,000 JPY	yes	0	0	0	0	0	0	0	1	1	1	1	1	1	1	1	1	1	1	1	1	1	1	4	4	4
1903	45	unmarried	5,000,000-6,000,000 JPY	no	0	3	0	0	0	0	0	3	1	1	1	1	1	1	1	1	1	1	1	1	1	1	1	1	1
1904	63	married	2,000,000-3,000,000 JPY	yes	1	1	1	1	2	2	0	2	1	1	1	2	2	2	1	1	1	3	1	5	1	1	2	3	2
1905	43	unmarried	4,000,000-5,000,000 JPY	no	0	0	0	0	0	0	1	3	1	1	1	1	1	1	1	1	1	1	1	1	1	1	1	1	1
1906	47	married	10,000,000-12,000,000 JPY	yes	0	1	1	0	1	0	2	4	2	3	1	3	3	4	3	2	3	3	3	3	1	1	1	1	1
1907	46	unmarried	8,000,000-9,000,000 JPY	no	0	0	0	0	0	0	1	3	3	1	1	5	3	3	4	5	4	3	4	2	3	1	5	5	3
1908	48	unmarried	less than 1,000,000 JPY	no	1	2	1	1	1	1	1	4	1	1	1	1	1	1	1	2	1	1	1	1	1	1	1	1	1
1909	69	married	2,000,000-3,000,000 JPY	yes	4	5	0	3	4	2	4	6	3	3	1	4	3	3	3	2	3	3	3	3	2	1	3	3	3
1910	62	unmarried	4,000,000-5,000,000 JPY	no	1	1	1	1	1	1	5	3	3	2	1	3	2	2	2	1	2	2	2	2	1	1	1	2	1
1911	68	married	5,000,000-6,000,000 JPY	yes	2	4	1	4	5	4	3	6	2	2	1	2	1	1	1	1	2	2	1	2	1	1	2	3	2
1912	44	unmarried	4,000,000-5,000,000 JPY	no	1	1	1	0	1	0	0	3	2	2	1	2	3	3	3	3	3	2	3	3	3	1	1	1	1
1913	60	married	9,000,000-10,000,000 JPY	yes	0	0	3	0	0	0	2	3	1	1	1	1	1	1	1	1	1	1	1	1	1	1	1	1	1

1914	62	unmarried	2,000,000-3,000,000 JPY	no	5	5	5	5	5	5	2	6	3	4	2	2	2	2	2	2	4	3	2	4	1	1	2	3	2
1915	61	married	10,000,000-12,000,000 JPY	yes	1	3	0	5	5	0	2	4	2	1	1	1	1	1	1	1	1	1	2	1	1	1	1	1	1
1916	60	married	6,000,000-7,000,000 JPY	yes	0	0	0	0	0	0	0	1	1	1	1	1	1	1	1	1	1	1	1	1	1	1	1	1	1
1917	68	unmarried	less than 1,000,000 JPY	no	0	0	0	0	0	0	2	1	2	2	1	1	1	3	2	2	2	1	3	2	2	1	1	1	1
1918	66	married	3,000,000-4,000,000 JPY	yes	2	1	2	1	2	2	0	5	2	3	3	2	2	3	2	1	2	2	2	3	1	1	2	2	2
1919	42	married	7,000,000-8,000,000 JPY	yes	0	0	0	0	0	0	5	0	1	1	1	1	1	1	3	3	3	3	3	5	1	3	3	1	1
1920	65	married	3,000,000-4,000,000 JPY	yes	0	0	0	0	0	0	1	2	1	1	1	1	1	1	1	1	1	1	1	1	1	1	1	1	1
1921	64	married	2,000,000-3,000,000 JPY	yes	0	1	0	0	0	0	0	2	1	1	1	1	1	1	1	1	1	1	1	2	1	1	2	1	1
1922	40	married	6,000,000-7,000,000 JPY	yes	0	0	0	0	0	0	0	3	1	1	1	1	1	1	1	1	1	1	1	1	1	1	1	1	1
1923	45	unmarried	4,000,000-5,000,000 JPY	no	5	5	3	5	1	5	1	6	3	3	4	4	3	3	3	2	2	3	4	4	3	1	4	1	4
1924	41	married	3,000,000-4,000,000 JPY	yes	4	4	4	4	4	0	5	5	2	1	1	2	2	1	1	1	1	1	1	2	2	1	2	2	2
1925	66	married	10,000,000-12,000,000 JPY	yes	0	2	0	0	5	0	2	3	2	1	1	1	1	2	1	1	2	2	1	4	1	1	3	1	3
1926	49	unmarried	1,000,000-2,000,000 JPY	no	1	5	5	5	0	5	2	3	1	2	1	3	3	2	2	1	3	3	2	3	3	1	2	2	2
1927	67	married	1,000,000-2,000,000 JPY	yes	5	0	0	0	0	0	0	4	1	3	1	2	1	2	1	1	1	3	1	2	2	1	2	3	1
1928	63	unmarried	8,000,000-9,000,000 JPY	no	1	1	1	0	0	0	3	2	3	3	3	3	3	2	4	2	1	3	5	4	2	4	3	3	2
1929	61	married	less than 1,000,000 JPY	yes	0	1	0	0	0	0	1	3	1	1	1	1	1	1	1	1	1	1	1	1	1	1	1	1	1
1930	68	unmarried	1,000,000-2,000,000 JPY	no	0	0	0	0	0	0	3	3	2	1	1	2	1	1	1	1	2	2	2	2	2	2	2	2	2
1931	40	married	6,000,000-7,000,000 JPY	yes	0	1	0	0	0	0	0	0	1	2	1	2	2	1	1	1	2	2	1	2	1	1	1	1	1
1932	42	unmarried	6,000,000-7,000,000 JPY	no	0	0	0	0	0	0	0	1	1	1	1	1	1	1	1	1	1	1	1	1	1	1	1	1	1
1933	47	unmarried	2,000,000-3,000,000 JPY	no	0	0	0	0	0	0	1	2	1	1	1	1	1	1	1	1	1	1	1	1	1	1	1	1	1
1934	47	married	7,000,000-8,000,000 JPY	yes	0	0	0	0	0	0	1	1	1	2	1	1	1	1	1	1	1	1	1	1	1	1	1	1	1
1935	45	married	12,000,000-15,000,000 JPY	no	1	1	0	3	0	0	1	3	2	3	3	2	3	1	2	2	3	4	2	4	4	1	4	4	2
1936	40	married	4,000,000-5,000,000 JPY	no	3	5	2	3	1	2	2	6	3	2	4	3	1	2	2	1	2	2	2	2	1	2	2	3	3
1937	42	married	10,000,000-12,000,000 JPY	no	0	0	0	0	0	0	0	1	1	1	1	1	1	1	1	1	1	1	1	1	1	1	1	1	1
1938	42	unmarried	7,000,000-8,000,000 JPY	no	0	1	1	0	1	1	5	1	1	1	1	1	2	2	1	1	1	1	1	1	1	1	1	1	1
1939	45	married	4,000,000-5,000,000 JPY	yes	1	5	0	0	1	0	3	3	4	4	1	3	4	2	3	2	4	4	3	5	4	1	2	3	3
1940	41	married	7,000,000-8,000,000 JPY	no	0	5	0	0	5	0	0	6	1	4	1	1	1	2	2	1	1	2	1	3	1	1	1	1	1
1941	62	married	3,000,000-4,000,000 JPY	no	5	5	5	5	5	5	1	1	1	1	1	1	1	1	1	1	1	1	1	1	1	1	2	2	2
1942	49	married	4,000,000-5,000,000 JPY	yes	0	2	0	0	0	0	1	3	2	3	2	2	2	1	2	1	1	1	1	1	1	1	2	2	1
1943	69	married	5,000,000-6,000,000 JPY	yes	0	1	0	0	0	0	1	4	3	3	1	1	1	1	1	1	1	2	1	3	1	1	2	4	4
1944	41	married	3,000,000-4,000,000 JPY	yes	0	0	0	0	1	1	0	1	2	2	2	1	2	2	2	2	3	2	3	3	2	1	2	3	1
1945	61	married	4,000,000-5,000,000 JPY	yes	1	1	1	0	5	0	2	5	2	2	1	2	2	1	1	1	2	2	2	4	1	1	2	3	2
1946	40	married	9,000,000-10,000,000 JPY	no	3	1	3	1	2	1	2	4	1	3	1	3	2	3	2	1	2	2	2	2	1	1	1	1	2
1947	47	married	5,000,000-6,000,000 JPY	yes	0	0	0	0	0	0	0	1	1	1	1	1	1	1	1	1	1	1	1	1	1	1	1	1	1
1948	63	married	7,000,000-8,000,000 JPY	yes	2	3	4	4	1	4	1	4	2	2	2	2	2	2	4	2	2	3	4	2	4	2	2	4	4
1949	43	married	7,000,000-8,000,000 JPY	yes	0	0	0	0	0	0	1	2	1	1	1	1	1	1	1	1	1	1	1	1	1	1	1	1	1
1950	66	married	2,000,000-3,000,000 JPY	yes	1	0	0	0	0	1	0	1	1	1	1	2	2	1	1	1	2	2	1	2	2	1	1	2	1
1951	48	married	20,000,000 or more JPY	yes	3	2	2	1	2	1	1	4	3	3	2	3	3	2	3	2	3	3	3	4	2	3	5	5	5
1952	66	married	2,000,000-3,000,000 JPY	yes	1	1	1	1	1	1	2	5	3	3	3	3	3	3	3	3	3	3	3	3	3	3	3	3	3
1953	61	married	less than 1,000,000 JPY	no	1	5	0	0	4	0	1	2	1	2	1	1	1	1	1	1	2	2	1	2	1	1	2	2	2
1954	43	unmarried	4,000,000-5,000,000 JPY	no	0	0	0	0	0	0	0	3	3	3	3	3	3	3	3	3	3	3	3	3	3	3	3	3	3
1955	68	married	6,000,000-7,000,000 JPY	yes	0	0	0	0	0	0	1	2	1	1	1	1	1	1	1	1	1	1	1	1	1	1	1	1	1
1956	67	married	4,000,000-5,000,000 JPY	yes	0	1	0	1	0	0	0	3	1	1	1	1	1	1	1	1	1	2	1	2	1	1	2	2	2
1957	61	married	5,000,000-6,000,000 JPY	yes	1	1	1	1	1	0	1	3	2	2	1	3	3	2	3	1	2	2	2	4	3	1	2	3	3
1958	62	married	1,000,000-2,000,000 JPY	yes	0	0	0	0	0	0	0	0	1	2	1	2	1	2	2	1	2	1	1	3	3	1	1	2	1
1959	67	unmarried	10,000,000-12,000,000 JPY	yes	1	1	3	0	3	3	0	4	3	2	2	3	3	1	2	2	3	3	3	4	3	2	4	4	4
1960	61	married	4,000,000-5,000,000 JPY	no	0	0	0	5	5	0	1	5	2	1	1	3	1	1	1	1	2	2	1	3	1	1	3	4	2
1961	61	married	15,000,000-18,000,000 JPY	yes	1	1	1	1	2	1	1	4	3	3	1	1	2	1	1	1	2	2	1	3	1	1	2	2	2
1962	68	married	less than 1,000,000 JPY	no	1	4	2	3	2	1	2	3	1	2	3	2	2	1	1	1	1	1	1	1	1	1	1	3	2
1963	64	married	6,000,000-7,000,000 JPY	yes	0	1	0	0	1	0	0	2	2	2	1	1	1	1	1	1	1	3	1	2	1	1	1	1	1
1964	64	married	10,000,000-12,000,000 JPY	yes	5	5	0	5	3	0	3	4	3	4	3	2	3	4	4	3	3	2	3	4	4	3	3	3	2
1965	60	married	12,000,000-15,000,000 JPY	yes	1	0	0	1	2	1	1	1	2	3	2	3	2	2	2	1	2	3	2	3	2	1	2	2	2
1966	66	married	1,000,000-2,000,000 JPY	yes	5	5	5	5	5	5	5	6	3	3	2	4	4	3	3	3	3	3	3	2	3	3	3	2	2
1967	63	married	2,000,000-3,000,000 JPY	yes	0	1	0	1	1	1	0	3	2	2	1	2	2	1	2	2	2	2	2	2	2	1	2	2	2
1968	67	unmarried	2,000,000-3,000,000 JPY	no	0	0	0	0	0	0	1	4	1	1	1	1	1	1	1	1	1	1	1	2	1	2	1	2	2
1969	61	married	5,000,000-6,000,000 JPY	yes	0	1	5	4	3	3	2	3	2	2	1	1	1	1	1	1	1	2	1	1	1	1	2	3	1
1970	63	married	5,000,000-6,000,000 JPY	yes	1	1	1	1	2	0	1	3	1	2	1	2	3	2	1	1	2	3	1	3	1	1	3	3	3
1971	63	married	7,000,000-8,000,000 JPY	no	1	1	0	0	1	0	1	3	2	1	1	1	2	2	1	1	2	2	1	2	1	1	2	2	1

1972	62	married	4,000,000-5,000,000 JPY	yes	1	1	0	0	0	0	1	1	1	1	1	1	1	1	1	1	1	1	1	1	1	1	1	1	1
1973	67	married	9,000,000-10,000,000 JPY	yes	2	2	3	3	4	4	2	5	2	2	1	2	2	1	1	1	1	2	1	4	4	1	4	4	3
1974	69	married	3,000,000-4,000,000 JPY	yes	0	1	1	1	1	1	0	4	3	3	2	4	2	3	3	3	3	3	3	3	3	2	3	3	3
1975	61	unmarried	2,000,000-3,000,000 JPY	no	0	1	0	0	0	0	0	1	1	1	1	2	2	1	1	1	1	2	1	3	1	1	2	4	2
1976	60	married	15,000,000-18,000,000 JPY	yes	0	0	0	0	0	0	0	1	2	1	1	1	1	1	1	1	1	1	1	1	1	1	1	1	1
1977	67	married	1,000,000-2,000,000 JPY	yes	0	1	0	0	1	1	2	2	2	2	3	2	2	2	2	1	2	2	1	3	1	2	2	1	2
1978	69	married	6,000,000-7,000,000 JPY	yes	1	1	0	3	5	0	1	4	1	1	1	3	2	1	1	1	1	2	1	3	1	2	4	1	1
1979	68	married	7,000,000-8,000,000 JPY	yes	0	0	0	0	0	0	0	0	1	1	1	1	1	1	1	1	1	1	1	1	1	1	1	1	1
1980	62	married	3,000,000-4,000,000 JPY	no	2	2	2	3	2	1	1	4	2	2	1	4	3	2	2	1	3	3	2	3	3	1	2	3	3
1981	60	married	1,000,000-2,000,000 JPY	yes	0	0	0	0	0	0	1	3	1	1	1	1	1	1	1	1	1	1	1	1	1	1	1	1	1
1982	60	married	3,000,000-4,000,000 JPY	yes	1	2	2	2	2	0	2	4	2	3	1	4	3	1	1	1	2	2	2	5	1	1	3	3	3
1983	63	married	4,000,000-5,000,000 JPY	yes	0	0	0	0	5	0	0	1	1	2	1	1	1	1	1	1	1	2	1	2	1	1	2	1	4
1984	64	married	7,000,000-8,000,000 JPY	no	5	1	5	5	1	5	2	2	3	3	2	2	3	2	1	1	2	3	1	3	2	1	2	2	2
1985	60	married	4,000,000-5,000,000 JPY	yes	1	2	1	1	4	0	1	5	3	4	2	4	3	4	4	4	4	4	4	4	3	3	4	4	4
1986	60	married	12,000,000-15,000,000 JPY	yes	0	2	1	1	0	0	1	3	2	2	1	2	3	2	2	1	2	2	2	3	2	1	2	2	2
1987	68	married	8,000,000-9,000,000 JPY	yes	0	0	0	0	0	0	0	0	1	1	1	1	1	1	1	1	1	2	1	1	1	1	2	2	1
1988	64	married	4,000,000-5,000,000 JPY	yes	5	5	5	5	5	0	1	4	3	3	1	1	2	3	2	1	2	2	2	4	2	2	4	4	4
1989	65	married	5,000,000-6,000,000 JPY	yes	0	0	0	0	0	0	1	0	1	2	1	1	1	2	2	2	1	3	2	2	1	1	4	4	4
1990	64	unmarried	less than 1,000,000 JPY	no	3	3	3	3	3	3	2	3	2	2	2	2	2	2	2	2	2	2	2	2	2	2	2	2	2
1991	63	married	1,000,000-2,000,000 JPY	yes	0	0	0	0	0	0	0	1	1	1	1	1	1	1	1	1	1	1	1	1	1	1	1	1	1
1992	64	married	4,000,000-5,000,000 JPY	yes	3	4	3	4	0	0	1	4	3	3	1	1	1	1	1	1	2	3	3	4	2	2	4	4	4
1993	68	married	2,000,000-3,000,000 JPY	yes	5	4	3	1	4	2	1	5	3	2	3	4	1	2	2	2	3	2	2	5	2	1	5	5	4
1994	64	married	6,000,000-7,000,000 JPY	yes	0	1	0	0	1	0	1	1	1	1	1	1	1	1	1	1	1	1	1	2	1	1	3	3	3
1995	61	married	3,000,000-4,000,000 JPY	yes	5	1	3	4	3	0	1	4	3	3	1	3	2	3	3	2	3	3	3	4	4	1	4	5	4
1996	64	married	4,000,000-5,000,000 JPY	no	0	1	0	0	2	0	0	3	1	2	2	1	1	1	1	1	3	4	1	3	1	4	4	4	4
1997	65	married	8,000,000-9,000,000 JPY	yes	2	2	5	1	5	1	2	5	3	3	2	4	2	2	2	2	2	3	2	3	2	1	3	2	3
1998	68	married	less than 1,000,000 JPY	no	1	2	0	1	1	0	1	3	2	3	2	3	2	2	3	2	3	2	4	4	2	1	3	3	2
1999	60	married	less than 1,000,000 JPY	yes	0	0	0	0	0	0	0	1	1	1	1	1	1	1	1	1	1	3	1	5	3	1	5	5	5
2000	61	married	4,000,000-5,000,000 JPY	yes	0	0	0	0	0	0	1	3	1	1	1	2	2	1	1	1	1	2	1	2	1	1	2	3	2
