# Supplementary figures and images for: Correlation between Higher Aging Males’ Symptoms Scores and a Higher Risk of Lower Urinary Tract Symptoms
Source: J Clin Med. 2023 Dec 6;12(24):7528. doi: 10.3390/jcm12247528 (PMC10744136; doi:10.3390/jcm12247528)

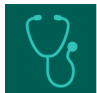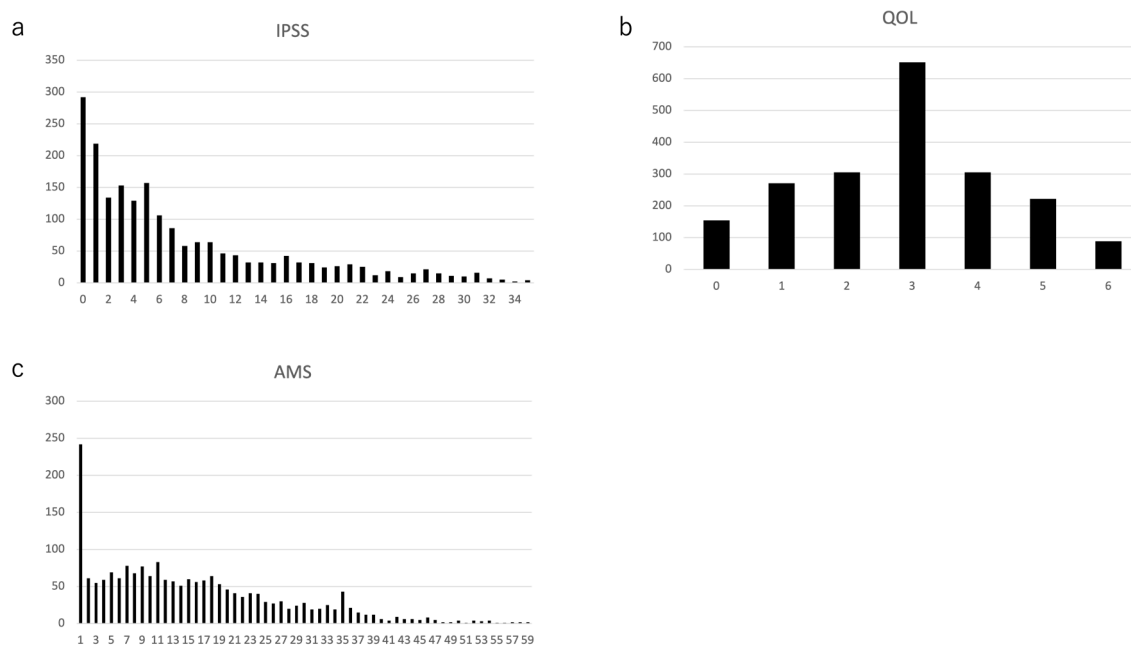

**Figure S1.** Distribution of (a) total IPSS score, (b) QOL score, and (c) AMS score.

Supplement: Supplementary file 1 [file jcm-12-07528-s001.zip › jcm-2706289-supplementary Figure S1.pdf]
